# Adverse events following SARS-CoV-2 mRNA vaccination in norwegian adolescents

**DOI:** 10.1038/s41598-026-45261-2

**Published:** 2026-03-27

**Authors:** Vilde Bergstad Larsen, Nina Gunnes, Jon Michael Gran, Jesper Dahl, Håkon Bøås, Sara Viksmoen Watle, Jacob Dag Berild, Margrethe Greve-Isdahl, Ketil Størdal, Hanne Løvdal Gulseth, Øystein Karlstad, Paz Lopez-Doriga Ruiz, German Tapia

**Affiliations:** 1 Division for Health Services, Norwegian Institute of Public Health, Oslo, Norway; 2 Division of Mental and Physical Health, Norwegian Institute of Public Health, Oslo, Norway; 3 Norwegian Research Centre for Women’s Health, Oslo University Hospital, Oslo, Norway; 4 Oslo Centre for Biostatistics and Epidemiology, Department of Biostatistics, University of Oslo, Oslo, Norway; 5 Department of Infection Control and Vaccines, Norwegian Institute of Public Health, Oslo, Norway; 6 Department of Pediatric Research, Institute of Clinical Medicine, University of Oslo, and Oslo University Hospital, Oslo, Norway; 7 Institute of Community Health and Global Medicine, University of Oslo, Oslo, Norway

**Keywords:** MRNA vaccine, Adolescents, Adverse events, Epidemiology, Diseases, Immunology, Medical research

## Abstract

The Norwegian COVID-19 vaccination campaign of adolescents from April 2021 necessitated surveillance of potential adverse events following immunization (AEFI). In this nationwide study of 496,432 adolescents, AEFI incidence rate ratios (IRRs) after first- and second-dose SARS-CoV-2 mRNA vaccination were compared to IRRs in unvaccinated subjects, and a self-controlled case series analysis was done as a secondary analysis. Seventeen pre-selected potential AEFIs were investigated: Anaphylactic reaction, Acute appendicitis, Lymphadenopathy, Arrhythmia, Cerebrovascular events, Death, Encephalomyelitis and meningitis, Epilepsy and convulsions, Facial nerve palsy, Herpes zoster, Idiopathic thrombocytopenic purpura, Myocarditis and pericarditis, Venous thromboembolic events, Arthropathy, Guillain-Barré syndrome, IgA vasculitis, and Multisystem inflammatory syndrome in children. Most AEFI were rare among adolescents, with few cases, and there was no statistically significant increase in the incidence of AEFIs after first-dose vaccination. Increased IRRs of anaphylactic reaction, lymphadenopathy, appendicitis, and myocarditis and pericarditis were observed following second-dose vaccination.

**Supplementary Information:**

The online version contains supplementary material available at 10.1038/s41598-026-45261-2.

## Introduction

Although severe acute respiratory syndrome coronavirus 2 (SARS-CoV-2) infection is milder in adolescents^1^, severe disease and post-infectious conditions may develop^2,3^. The Norwegian COVID-19 vaccination campaign offered healthy adolescents (born 2002–2009) vaccination from April 2021, while those with chronic conditions were vaccinated earlier. There was no strong recommendation to vaccinate healthy adolescents < 16 years, which might have resulted in a less selected unvaccinated population. Tozinameran (Comirnaty/BNT162b2, Pfizer-BioNTech) and elasomeran (Spikevax/mRNA-1273, Moderna) were administered in Norway, with adolescents predominantly receiving tozinameran.

Phase 3 clinical trials of mRNA vaccines reported increased risks of lymphadenopathy and Bell’s palsy^4–6^, and higher proportions of appendicitis, acute myocardial infarction, and cerebrovascular accidents in vaccinees^4^ Vaccine trials of tozinameran in children (5–11 years, *n* = 2,268), adolescents (12–15 years, *n* = 2,260) and elasomeran in adolescents (12–17 years, *n* = 3,732) reported no severe adverse events following immunization (AEFI)^7–9^.

Clinical trials are by necessity small and usually include healthier subjects. Larger cohort studies are needed for post-marketing surveillance of potential AEFIs. An adult study found tozinameran associated with myocarditis, lymphadenopathy, appendicitis, and herpes zoster^10^ Studies in children/adolescents have reported increased risk of myocarditis^11–13^and epilepsy^11^, but there are also null findings^14,15^ Investigations of potential AEFIs in adolescents are warranted to fill knowledge gaps and ensure trust in vaccines.

In this nationwide study, we aimed to assess the short- and mid-term SARS-CoV-2 mRNA vaccine safety in 12–19-year-olds by investigating potential preselected AEFIs using both cohort and self-controlled case series (SCCS) study designs.

## Results

**Fig. 1:**
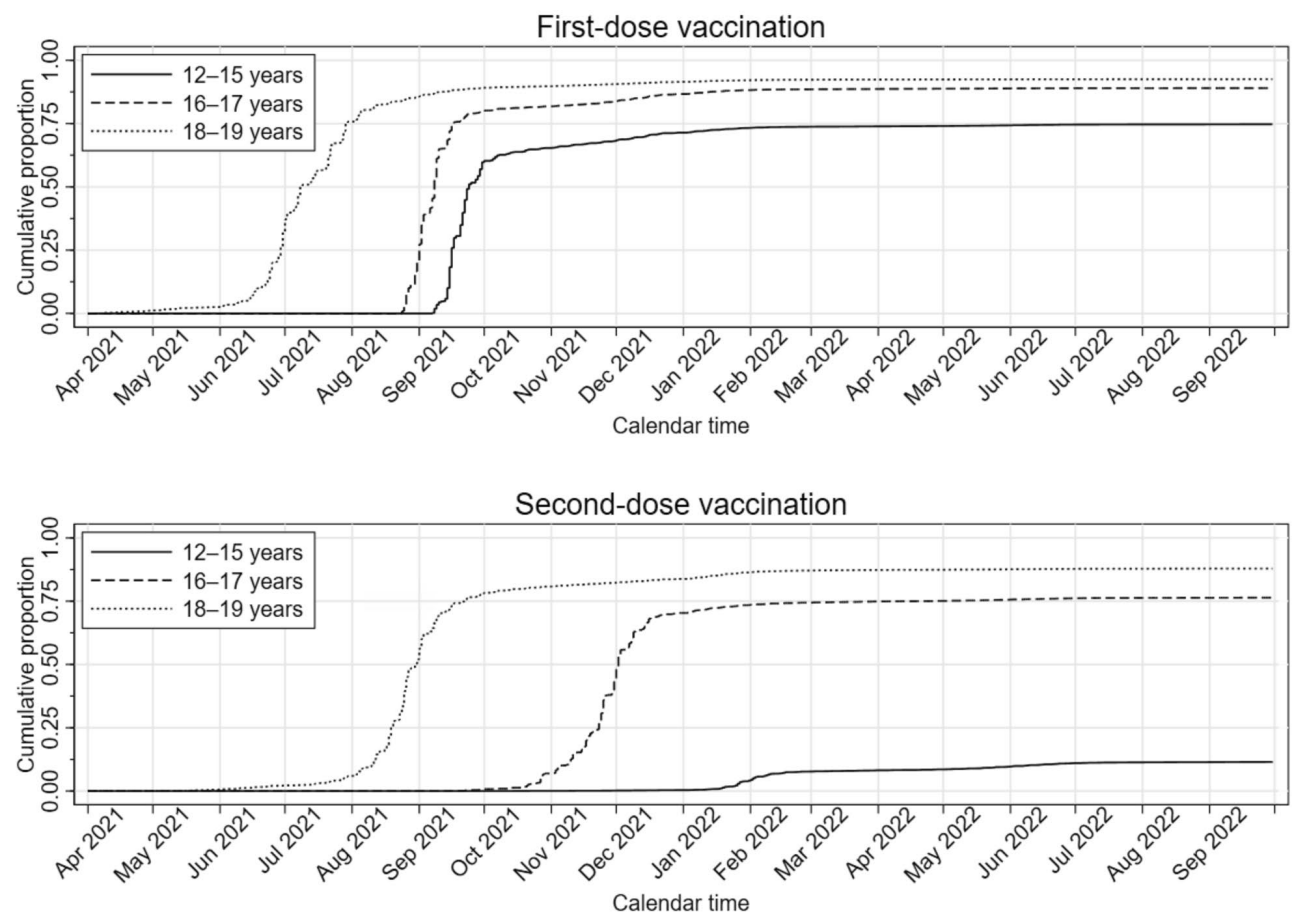
Kaplan–Meier estimate of the cumulative proportion of included study subjects vaccinated against SARS-CoV-2 with an mRNA vaccine, tozinameran (Comirnaty/BNT162b2, Pfizer-BioNTech) or elasomeran (Spikevax/mRNA-1273, Moderna), as a function of calendar time by age group for first-dose vaccination (upper panel) and second-dose vaccination (lower panel). Individuals are censored at the date of receiving the relevant vaccine dose.

Table 1 displays cohort characteristics. The proportions of 12–15-year-olds, 16–17-year-olds, and 18–19-year-olds were 51.1%, 24.4%, and 24.5%, respectively, with a slight male predominance (51.4%). There was a rapid increase in vaccination coverage the first months of the vaccination waves (Fig. 1).

**Table 1:** Characteristics of 496,432 adolescents born in 2002–2009 and still alive, residing in Norway (since January 1, 2017, or earlier), and unvaccinated against SARS-CoV-2 at the beginning of the wave of vaccination1 of their age group.

Variable	Total		Unvaccinated^2^		Vaccinated^2^					
					One dose		Two doses		More than two doses	
Total	496,432	(100%)	87,086	(100%)	181,556	(100%)	168,698	(100%)	59,092	(100%)
*Sex*										
Males	255,172	(51.4%)	46,743	(53.7%)	94,424	(52.0%)	86,932	(51.5%)	27,073	(45.8%)
Females	241,260	(48.6%)	40,343	(46.3%)	87,132	(48.0%)	81,766	(48.5%)	32,019	(54.2%)
Age group^3^										
12–15 years	253,669	(51.1%)	64,021	(73.5%)	160,440	(88.4%)	29,137	(17.3%)	71	(0.1%)
16–17 years	121,179	(24.4%)	13,303	(15.3%)	15,317	(8.4%)	90,778	(53.8%)	1,781	(3.0%)
18–19 years	121,584	(24.5%)	9,762	(11.2%)	5,799	(3.2%)	48,783	(28.9%)	57,240	(96.9%)
Health region^4^										
North	42,126	(8.5%)	6,243	(7.2%)	15,095	(8.3%)	16,630	(9.9%)	4,158	(7.0%)
Central	68,134	(13.7%)	8,644	(9.9%)	25,150	(13.9%)	24,769	(14.7%)	9,571	(16.2%)
West	107,955	(21.7%)	19,831	(22.8%)	39,777	(21.9%)	35,500	(21.0%)	12,847	(21.7%)
South-East	276,368	(55.7%)	51,268	(58.9%)	101,254	(55.8%)	91,509	(54.2%)	32,337	(54.7%)
Unknown	1,849	(0.4%)	1,100	(1.3%)	280	(0.2%)	290	(0.2%)	179	(0.3%)
Risk group^5^										
No	455,004	(91.7%)	81,189	(93.2%)	166,177	(91.5%)	154,014	(91.3%)	53,624	(90.7%)
Yes	41,428	(8.3%)	5,897	(6.8%)	15,379	(8.5%)	14,684	(8.7%)	5,468	(9.3%)
First-dose vaccine										
Tozinameran (Comirnaty/Pfizer-BioNTech)	396,141	(79.8%)	0	(0.0%)	180,065	(99.2%)	160,183	(95.0%)	55,893	(94.6%)
Elasomeran (Spikevax/Moderna)	13,069	(2.6%)	0	(0.0%)	1,415	(0.8%)	8,462	(5.0%)	3,192	(5.4%)
Other^6^	< 49	(0.0%)	0	(0.0%)	19	(0.0%)	25	(0.0%)	< 5	(0.0%)
Unknown	< 90	(0.0%)	0	(0.0%)	57	(0.0%)	28	(0.0%)	< 5	(0.0%)
First dose not received	87,086	(17.5%)	87,086	(100.0%)	0	(0.0%)	0	(0.0%)	0	(0.0%)
Second-dose vaccine										
Tozinameran (Comirnaty/Pfizer-BioNTech)	190,006	(38.3%)	0	(0.0%)	0	(0.0%)	150,778	(89.4%)	39,228	(66.4%)
Elasomeran (Spikevax/Moderna)	37,705	(7.6%)	0	(0.0%)	0	(0.0%)	17,846	(10.6%)	19,859	(33.6%)
Other^6^	< 24	(0.0%)	0	(0.0%)	0	(0.0%)	19	(0.0%)	< 5	(0.0%)
Unknown	< 60	(0.0%)	0	(0.0%)	0	(0.0%)	55	(0.0%)	< 5	(0.0%)
Second dose not received	268,642	(54.1%)	87,086	(100.0%)	181,556	(100.0%)	0	(0.0%)	0	(0.0%)

To ensure data privacy, numbers between 1 and 4 have been suppressed and are denoted by “ < 5”. As a result, some of the totals have been suppressed as well to avoid revealing small numbers.

^1^12–15-year-olds: September 6, 2021; 16–17-year-olds: August 23, 2021; 18–19-year-olds: April 5, 2021.

^2^Status by September 30, 2022.

^3^Based on attained aged on December 31, 2021.

^4^Based on county of residence.

^5^Based on the following conditions: asthma and other chronic cardiopulmonary conditions, cerebral palsy and other neuromuscular disorders, Down’s syndrome and other chromosomal conditions, cancer, transplantation, immunodeficiencies, liver/kidney disorders, and autoimmune disorders.

^6^Including the following vaccines: Janssen (Johnson & Johnson), Nuvaxovid (Novavax), Vaxzevria (Oxford/AstraZeneca), CoronaVac (Sinovac), and Sinopharm BIBP (Sinopharm).

By end-of-study (September 30, 2022), 82.5% had completed first-dose vaccination (Table 1). Furthermore, 11.5%, 76.4% and 87.2% of subjects aged 12–15, 16–17 and 18–19 years, respectively, had received a second dose.

### Cohort analysis

Main results are shown in Table 2, and excess events are shown in Supplementary Table 1. In the main analysis, there were no statistically significant increases in AEFI incidences after first-dose vaccination within the risk windows (Table 2). These results were consistent when restricting the analysis to subjects without reported infections (Supplemental Table 2). In age-stratified analyses (Supplementary Table 3), first-dose vaccination in 12–15-year-olds associated with increased incidence of acute appendicitis (adjusted IRR [aIRR]: 1.91; 95% CI: 1.12–3.27) and anaphylactic reaction (aIRR: 16.19; 95% CI: 1.29–202.98), which should be interpreted cautiously due to small numbers.

**Table 2:** Crude and adjusted incidence rate ratios of 17 different outcomes between vaccinated and unvaccinated subjects, with associated 95% confidence intervals, based on Poisson regression of 496,432 adolescents in Norway aged 12–19 years at the end of 2021 and unvaccinated against SARS-CoV-2 at the beginning of follow-up. Subjects were followed from the beginning of the wave of vaccination1 of their age group until the outcome in question, non-mRNA SARS-CoV-2 vaccination, third-dose SARS-CoV-2 vaccination, emigration, death, or end of study on September 30, 2022, whichever occurred first. To ensure data privacy, numbers between 1 and 4 have been suppressed and are denoted by “ < 5”. As a result, some of the totals have been suppressed as well to avoid revealing small numbers that have been suppressed.

Outcome	Risk window	Vaccination status	Subjects	Events	Incidence rate^2^	Crude analysis				Adjusted analysis^3^	
						IRR	95% CI	P value	IRR	95% CI	P value
Acute appendicitis	14 days	Overall	492,360	1,185	220.21						
		Unvaccinated (ref.)	491,589	267	181.88	1			1		
		1^st^ dose, inside risk window	405,689	36	231.53	1.27	0.90–1.80	0.174	1.29	0.89–1.86	0.176
		1^st^ dose, outside risk window	405,588	495	234.99	1.29	1.11–1.50	0.001	1.49	1.26–1.77	< 0.001
		2^nd^ dose, inside risk window	225,523	21	243.03	1.34	0.86–2.08	0.201	1.34	0.85–2.11	0.213
		2^nd^ dose, outside risk window	225,341	366	233.88	1.29	1.10–1.51	0.002	1.33	1.08–1.64	0.008
Anaphylactic reaction	2 days	Overall	496,226	< 61	10.31						
		Unvaccinated (ref.)	495,443	12	8.11	1			1		
		1^st^ dose, inside risk window	409,035	< 5	44.65	5.51	0.72–42.35	0.101	5.17	0.64–41.97	0.124
		1^st^ dose, outside risk window	409,021	26	11.51	1.42	0.72–2.81	0.316	2.13	0.94–4.82	0.068
		2^nd^ dose, inside risk window	227,541	< 5	80.26	9.90	1.29–76.14	0.028	10.05	1.22–82.74	0.032
		2^nd^ dose, outside risk window	227,507	16	9.67	1.19	0.56–2.52	0.645	1.08	0.40–2.91	0.872
Arrhythmia	28 days	Overall	495,835	135	24.88						
		Unvaccinated (ref.)	495,054	39	26.37	1			1		
		1^st^ dose, inside risk window	408,676	< 5	12.80	0.49	0.17–1.36	0.168	0.43	0.15–1.27	0.127
		1^st^ dose, outside risk window	397,465	43	21.85	0.83	0.54–1.28	0.395	1.02	0.63–1.65	0.933
		2^nd^ dose, inside risk window	227,291	< 5	5.74	0.22	0.03–1.59	0.132	0.16	0.02–1.21	0.076
		2^nd^ dose, outside risk window	226,911	48	32.18	1.22	0.80–1.86	0.356	0.95	0.55–1.66	0.863
Arthropathy	42 days	Overall	496,414	0	0.00						
		Unvaccinated (ref.)	495,631	0	0.00	1			1		
		1^st^ dose, inside risk window	409,206	0	0.00	ND	ND	ND	ND	ND	ND
		1^st^ dose, outside risk window	362,098	0	0.00	ND	ND	ND	ND	ND	ND
		2^nd^ dose, inside risk window	227,662	0	0.00	ND	ND	ND	ND	ND	ND
		2^nd^ dose, outside risk window	227,093	0	0.00	ND	ND	ND	ND	ND	ND
Cerebrovascular events	28 days	Overall	496,307	< 33	5.71						
		Unvaccinated (ref.)	495,524	10	6.76	1			1		
		1^st^ dose, inside risk window	409,109	< 5	9.59	1.42	0.39–5.16	0.595	1.52	0.35–6.52	0.575
		1^st^ dose, outside risk window	397,881	12	6.09	0.90	0.39–2.09	0.809	1.02	0.39–2.66	0.973
		2^nd^ dose, inside risk window	227,587	0	0.00	ND	ND	ND	ND	ND	ND
		2^nd^ dose, outside risk window	227,208	6	4.02	0.59	0.22–1.64	0.314	0.47	0.13–1.67	0.245
Death (all-cause mortality)	28 days	Overall	496,414	109	20.06						
		Unvaccinated (ref.)	495,631	41	27.69	1			1		
		1^st^ dose, inside risk window	409,206	7	22.37	0.81	0.36–1.80	0.602	0.75	0.32–1.79	0.523
		1^st^ dose, outside risk window	397,975	23	11.67	0.42	0.25–0.70	0.001	0.58	0.33–1.02	0.058
		2^nd^ dose, inside risk window	227,662	5	28.67	1.04	0.41–2.62	0.941	0.80	0.30–2.13	0.658
		2^nd^ dose, outside risk window	227,283	33	22.08	0.80	0.50–1.26	0.333	0.60	0.33–1.10	0.101
Encephalomyelitis and meningitis	28 days	Overall	496,349	14	2.58						
		Unvaccinated (ref.)	495,566	< 5	2.70	1			1		
		1^st^ dose, inside risk window	409,153	0	0.00	ND	ND	ND	ND	ND	ND
		1^st^ dose, outside risk window	397,924	5	2.54	0.94	0.25–3.50	0.926	0.90	0.23–3.47	0.878
		2^nd^ dose, inside risk window	227,629	< 5	5.74	2.12	0.24–18.99	0.502	1.58	0.15–16.23	0.701
		2^nd^ dose, outside risk window	227,250	< 5	2.68	0.99	0.25–3.96	0.990	0.35	0.08–1.58	0.171
Epilepsy and convulsions	28 days	Overall	491,327	617	114.82						
		Unvaccinated (ref.)	490,551	165	112.67	1			1		
		1^st^ dose, inside risk window	405,001	29	93.62	0.83	0.56–1.23	0.358	0.91	0.60–1.38	0.661
		1^st^ dose, outside risk window	393,882	225	115.43	1.02	0.84–1.25	0.814	1.00	0.80–1.24	0.972
		2^nd^ dose, inside risk window	225,243	30	173.89	1.54	1.05–2.28	0.029	1.49	0.99–2.26	0.056
		2^nd^ dose, outside risk window	224,841	168	113.69	1.01	0.81–1.25	0.935	0.90	0.68–1.19	0.442
Facial nerve palsy	28 days	Overall	496,027	91	16.77						
		Unvaccinated (ref.)	495,244	34	22.98	1			1		
		1^st^ dose, inside risk window	408,885	< 5	12.79	0.56	0.20–1.57	0.268	0.52	0.17–1.52	0.231
		1^st^ dose, outside risk window	397,659	20	10.16	0.44	0.25–0.77	0.004	0.44	0.24–0.78	0.006
		2^nd^ dose, inside risk window	227,469	< 5	11.48	0.50	0.12–2.08	0.340	0.42	0.10–1.82	0.246
		2^nd^ dose, outside risk window	227,088	31	20.77	0.90	0.56–1.47	0.683	0.79	0.41–1.52	0.484
Guillain-Barré syndrome	42 days	Overall	496,401	6	1.10						
		Unvaccinated (ref.)	495,618	0	0.00	1			1		
		1^st^ dose, inside risk window	409,193	0	0.00	ND	ND	ND	ND	ND	ND
		1^st^ dose, outside risk window	362,086	< 5	1.10	ND	ND	ND	ND	ND	ND
		2^nd^ dose, inside risk window	227,652	0	0.00	ND	ND	ND	ND	ND	ND
		2^nd^ dose, outside risk window	227,083	< 5	2.84	ND	ND	ND	ND	ND	ND
IgA vasculitis	42 days	Overall	496,343	< 17	2.58						
		Unvaccinated (ref.)	495,560	< 5	1.35	1			1		
		1^st^ dose, inside risk window	409,149	0	0.00	ND	ND	ND	ND	ND	ND
		1^st^ dose, outside risk window	362,048	7	3.84	2.84	0.59–13.69	0.192	3.07	0.51–18.36	0.219
		2^nd^ dose, inside risk window	227,633	0	0.00	ND	ND	ND	ND	ND	ND
		2^nd^ dose, outside risk window	227,064	5	3.55	2.63	0.51–13.56	0.248	2.38	0.28–20.33	0.429
Herpes zoster	28 days	Overall	496,181	80	14.73						
		Unvaccinated (ref.)	495,398	18	12.16	1			1		
		1^st^ dose, inside risk window	409,001	< 5	6.39	0.53	0.12–2.27	0.388	0.81	0.17–3.86	0.788
		1^st^ dose, outside risk window	397,775	29	14.73	1.21	0.67–2.18	0.524	0.96	0.51–1.81	0.894
		2^nd^ dose, inside risk window	227,530	< 5	5.74	0.47	0.06–3.53	0.465	0.57	0.07–4.44	0.593
		2^nd^ dose, outside risk window	227,151	30	20.09	1.65	0.92–2.96	0.092	1.09	0.52–2.30	0.814
Idiopathic thrombocytopenic	28 days	Overall	496,289	24	4.42						
purpura		Unvaccinated (ref.)	495,506	6	4.05	1			1		
		1^st^ dose, inside risk window	409,107	< 5	6.39	1.58	0.32–7.81	0.577	1.01	0.19–5.41	0.987
		1^st^ dose, outside risk window	397,881	8	4.06	1.00	0.35–2.89	0.997	1.42	0.41–4.88	0.581
		2^nd^ dose, inside risk window	227,605	< 5	5.74	1.41	0.17–11.75	0.748	1.34	0.14–12.93	0.799
		2^nd^ dose, outside risk window	227,227	7	4.69	1.16	0.39–3.44	0.795	2.78	0.61–12.75	0.188
Lymphadenopathy	14 days	Overall	494,138	651	120.47						
		Unvaccinated (ref.)	493,360	152	103.16	1			1		
		1^st^ dose, inside risk window	407,246	15	96.10	0.93	0.55–1.58	0.793	1.04	0.60–1.81	0.884
		1^st^ dose, outside risk window	407,166	249	117.71	1.14	0.93–1.40	0.200	1.16	0.93–1.45	0.194
		2^nd^ dose, inside risk window	226,468	22	253.55	2.46	1.57–3.84	0.000	2.33	1.46–3.72	< 0.001
		2^nd^ dose, outside risk window	226,284	213	135.47	1.31	1.07–1.62	0.010	1.17	0.89–1.54	0.257
Multisystem inflammatory	42 days	Overall	496,389	21	3.87						
syndrome in children		Unvaccinated (ref.)	495,606	6	4.05	1			1		
		1^st^ dose, inside risk window	409,187	< 5	6.51	1.61	0.40–6.43	0.502	1.14	0.27–4.87	0.862
		1^st^ dose, outside risk window	362,079	8	4.39	1.08	0.38–3.12	0.882	0.96	0.31–2.98	0.948
		2^nd^ dose, inside risk window	227,656	0	0.00	ND	ND	ND	ND	ND	ND
		2^nd^ dose, outside risk window	227,087	< 5	2.84	0.70	0.20–2.49	0.583	0.81	0.17–3.94	0.798
Myocarditis and pericarditis	28 days	Overall	496,359	< 68	12.15						
		Unvaccinated (ref.)	495,576	13	8.78	1			1		
		1^st^ dose, inside risk window	409,150	< 5	9.59	1.09	0.31–3.83	0.891	0.99	0.25–3.89	0.993
		1^st^ dose, outside risk window	397,921	17	8.63	0.98	0.48–2.02	0.962	2.38	0.96–5.92	0.062
		2^nd^ dose, inside risk window	227,609	11	63.09	7.19	3.22–16.04	0.000	5.27	1.98–14.05	0.001
		2^nd^ dose, outside risk window	227,219	22	14.73	1.68	0.84–3.33	0.139	1.84	0.68–4.94	0.228
Venous thromboembolic events	28 days	Overall	496,310	< 71	12.71						
		Unvaccinated (ref.)	495,527	11	7.43	1			1		
		1^st^ dose, inside risk window	409,104	5	15.98	2.15	0.75–6.19	0.156	2.17	0.65–7.18	0.206
		1^st^ dose, outside risk window	397,875	20	10.15	1.37	0.65–2.85	0.406	2.49	0.98–6.34	0.057
		2^nd^ dose, inside risk window	227,564	< 5	17.21	2.32	0.65–8.30	0.197	1.94	0.48–7.89	0.352
		2^nd^ dose, outside risk window	227,182	30	20.09	2.70	1.35–5.39	0.005	2.55	0.93–7.02	0.070

Abbreviations: CI – confidence interval; IRR – incidence rate ratio; mRNA – messenger RNA; ND – not determined.

^1^12–15-year-olds: September 6, 2021; 16–17-year-olds: August 23, 2021; 18–19-year-olds: April 5, 2021.

^2^Per 100,000 person-years.

^3^Adjustment for sex (male or female), attained age by the end of 2021 (12–15 years, 16–17 years, or 18–19 years), health region (North Norway, Central Norway, West Norway, or South-East Norway), and risk group (no or yes) as baseline covariates and three-month calendar period (April–June 2021, July–September 2021, October–December 2021, January–March 2022, April–June 2022, and July–September 2022) as a time-varying covariate.

Second-dose vaccination associated with increased incidence of anaphylactic reaction (aIRR: 10.05; 95% CI: 1.22–82.74, but few cases were observed), lymphadenopathy (aIRR 2.33; 95% CI: 1.46–3.72), and myocarditis and pericarditis in the main analysis (aIRR 5.27; 95% CI: 1.98–14.05). In the sensitivity analysis restricted to subjects without reported infections, results were consistent, except an increased incidence of epilepsy and convulsions following second-dose vaccination (aIRR: 1.65; 95% CI: 1.05–2.59). In the age-stratified sensitivity analyses, second-dose vaccination associated with myocarditis and pericarditis in 18–19-year-olds and 12–15-year-olds (aIRR: 10.25; 95% CI: 2.36–44.47, and aIRR: 37.07; 95% CI: 2.79–492.94, respectively), but there were few cases in the youngest age-group. 18–19-year-olds also had increased incidence of anaphylactic reactions (aIRR: 38.78; 95% CI: 3.46–434.39, but few cases were observed), epilepsy and convulsions (aIRR: 2.20; 95% CI: 1.05–4.61) and lymphadenopathy (aIRR: 2.37; 95% CI: 1.09–5.15) following second-dose vaccination. In 16–17-year-olds, second-dose vaccination associated with increased acute appendicitis incidence (aIRR: 2.81; 95% CI: 1.30–6.09).

After the risk windows, we observed statistically significant associations between vaccination and acute appendicitis and facial nerve palsy (Table 2). We also observed some statistically significant associations after the risk windows when restricting the analysis to subjects without reported infections (acute appendicitis, anaphylactic reaction, all-cause death, and myocarditis and pericarditis; Supplementary Table 2), and in the age-stratified sensitivity analysis (acute appendicitis, facial nerve palsy, all-cause death, anaphylaxis; Supplemental Table 3).

**Table 3:** Conditions and the corresponding ICD-10^1^ codes that define the outcomes of interest.

Condition	ICD-10^1^ codes	Risk window	Inpatient/outpatient
Acute appendicitis	K35	14 days	Inpatient
Anaphylactic reaction	R57.9, T78.2, T88.6	2 days	Inpatient
Arrhythmia	I44, I45, I46, I47, I48, I49	28 days	Inpatient
Arthropathy	M02.2, M02.9	42 days	In- and outpatient
Cerebrovascular events, combined	G45, I60, I61, I62, I63, I64	28 days	Inpatient
Death^2^	Any ICD-10 code	28 days	-
Encephalomyelitis and meningitis, combined	G03.0, G04.0, G04.8, G04.9, G36.9	28 days	Inpatient
Epilepsy and convulsions, combined	G40, G41, R56	28 days	In- and outpatient
Facial nerve palsy	G51.0, G51.9	28 days	In- and outpatient
Guillain-Barré syndrome	G61.0	42 days	Inpatient
IgA vasculitis	D69.0, M36.4, N08.2	42 days	Inpatient
Herpes zoster	B02, S70^3^	28 days	In- and outpatient
Idiopathic thrombocytopenic purpura	D69.3	28 days	In- and outpatient
Lymphadenopathy	I88.0, I88.8, I88.9, L04, R59	14 days	In- and outpatient
Multisystem inflammatory syndrome in children	B94.8, M35.8, U10.9	42 days	Inpatient
Myocarditis and pericarditis, combined	I30.0, I30.8, I30.9, I40.1, I40.8, I40.9, I51.4	28 days	Inpatient
Venous thromboembolic events, combined	I26, I63.6, I67.6, I80.1, I80.2, I80.3, I81, I82.0, I82.2, I82.3, I82.8, I82.9	28 days	In- and outpatient

^1^International statistical classification of diseases and related health problems, tenth revision (ICD-10).

^2^Date of death obtained from the Norwegian population register.

^3^Code based on the international classification of primary care, second edition (ICPC-2).

### Post-hoc analyses

We observed increased acute appendicitis incidence after the 14-day risk window following first- and second-dose vaccinations (main analysis, Table 2). Therefore, we conducted post-hoc analyses with longer risk windows (28, 42, and 56 days) to explore whether the disease manifestation might be longer than presumed.

We observed no statistically significant difference in acute appendicitis incidence for first- or second-dose vaccination inside the 28-day risk window. After 42 days there was increased incidence following both first (aIRR: 1.39; 95% CI: 1.09–1.78) and second-dose vaccination (aIRR: 1.43; 95% CI: 1.07–1.91). The same was observed using 56 days (aIRRs: 1.47; 95% CI: 1.17–1.83 and 1.44; 95% CI: 1.10–1.88, respectively). These results are data-driven, speculative and must be interpreted cautiously.

### Self-controlled case series analysis

Supplementary Table 4 displays the SCCS analysis results. We observed no statistically significant incidence increase following first-dose vaccination inside the risk windows. As in the cohort analysis, second-dose vaccination associated with increased incidence of lymphadenopathy (aIRR: 2.04; 95% CI: 1.24–3.35) and myocarditis and pericarditis (aIRR: 5.88; 95% CI: 2.11–16.40). We observed a statistically significant increase of herpes zoster incidence (aIRR: 4.12: 95%CI: 1.10–15.41) following second-dose vaccination after the second risk window. There was a tendency towards increased anaphylactic reaction risk following second-dose vaccination, based on few cases, and epilepsy and convulsions.

In the sensitivity analysis excluding a 14-day preexposure risk period, results remained mostly unchanged. Second-dose vaccination associations with lymphadenopathy, and myocarditis and pericarditis, were attenuated (aIRRs 1.78 [95% CI: 1.06–2.97] and 4.55 [95% CI: 1.62–12.68], respectively), whereas the herpes zoster association lost statistical significance.

## Discussion

This nationwide study is one of the few investigating a broad spectrum of preselected AEFIs in adolescents. We confirm the SARS-CoV-2 mRNA vaccine safety, with no association between AEFIs and first-dose vaccination in the main analysis. Second-dose vaccination associated with anaphylaxis, lymphadenopathy, and myocarditis/pericarditis. Increased incidence of acute appendicitis following vaccination was observed in age-stratified analyses.

The main strength was individual-level data from high-quality nationwide mandatory registers. Norwegian health care and SARS-CoV-2 vaccination is nominally free of charge, reducing socioeconomic confounding. By applying different designs, we could check the robustness of our results. We adjusted for some potential confounders in the cohort analysis, while time-invariant confounders were implicitly adjusted in the SCCS analysis^16,17^. High vaccination coverage means AEFIs should be rare or weakly associated to remain undetected. We excluded subjects vaccinated prior to their age-specific vaccination wave, as off-label use was reserved for high-risk groups. The 18-month study period was not limited to early vaccinees, who might constitute a selected population (e.g. underlying diseases, or high socioeconomic status). Adjusting for calendar time lessened the risk of temporal associations influencing results, mitigating effects of lifestyle changes, pandemic restrictions, or other unmeasured time-correlated factors.

Few comparable studies are currently published (for an overview, see Supplemental Material and Supplemental Table 5). One study investigated long-term AEFIs and report only protective associations^14^. Three studies report an association between mRNA vaccination and myocarditis^11–13^, and one also reports an epilepsy association^11^. Yet, differences in ages, risk windows, and diagnostic coding in addition to national differences in policy, infection and vaccination rates during the COVID-19 pandemic make comparisons complex.

In our main analysis, we found no significantly increased appendicitis incidence inside the risk windows, and no associations in the SCCS analysis. There was an association using longer risk windows, but Copland et al. did not observe this^11^. In age-stratified analyses, we observed increased incidence following first-dose vaccination in 12–15-year-olds and second-dose vaccination in 16–17-year-olds. Two studies report subgroup findings in 12–17-years-olds – Copland et al. only in an unadjusted matched cohort after first-dose vaccination^11^, while Dorajoo et al. report increased risk in males following first dose, and females following second dose in a SCCS analysis^13^. These subgroup findings are inconsistent and must be interpreted very cautiously, but should be investigated in 12–17-year-olds in other cohorts.

Anaphylactic reaction was rare, with < 5 cases after both doses. Copland et al. report increased risk following first-dose vaccination in a matched cohort analysis^11^, while Lai et al. report no increased risk^12^. We observed increased incidence following second-dose vaccination.

We found no statistically significant associations with all-cause mortality within 28 days. Events were very rare. No Norwegian adolescents were registered with vaccine-associated death (ICD-10 code U12.9) during follow-up.

Two studies found no statistically significant epilepsy association^12,15^. Copland et al. report increased incidence following second-dose vaccination^11^, as we do in uninfected subjects and 18–19-year-olds in the cohort analysis. Copland et al. argue their results are likely prevalent epilepsy^11^. Uninfected subjects could be overrepresented by subjects with increased epilepsy and convulsion risk, who presumably adhered more strictly to protective measures. Many cases are likely febrile seizures in our study. These results should be interpreted very cautiously.

Data from the Pfizer clinical trial reported four vaccinees vs zero controls with Bell’s palsy^4^, but other studies report no statistically significant association^10,18–22^, supporting our results.

Some studies report increased post-vaccination herpes zoster incidence in adults and adolescent males^10,23^, whereas others do not^20^. We found no consistent statistically significant association, with few cases observed.

Lymphadenopathy is a common post-vaccination event, reported as being more common in vaccinees in two multinational clinical trials and a nationwide Israeli study^6,8,10^, supporting our results.

A previous Nordic study reported increased myocarditis risk in ages ≥ 12 years^24^, as have adolescent studies^10–13,23,25^, particularly after the second dose^11,26^. This was not observed in clinical trials^5,7–9^, nor in two other studies^15,22^. Our results support increased myocarditis and pericarditis incidence after vaccination. Myocarditis may lead to arrhythmia, but we observed no vaccine-arrhythmia association. Other studies reporting increased post-vaccination myocarditis incidence similarly report no vaccine-arrhythmia association^10,12,27^, except a Malaysian study^22^. Post-vaccination myocarditis might be milder^25,28^, resulting in fewer arrhythmias.

Cerebrovascular events were grouped in the current study. While individually rare in adolescents, they could be different manifestations of a common pathology. Similar reasoning was used when grouping venous thromboembolic events. Adult studies report both increased incidence of venous thromboembolic events and hemorrhagic events following vaccination^22,29^, and no association^30–32^. A Nordic adult study found no consistent associations with cerebrovascular or coagulation disorders^33^, whereas an adolescent study reported no associations with thromboembolic or cerebrovascular events^12^. A study in 16–19-year-olds reported no associations between vaccination and deep vein thrombosis or pulmonary embolism^23^. We found no statistically significant associations between vaccination and cerebrovascular events or venous thromboembolic events. Estimates of venous thromboembolic events were elevated following first- and second-dose vaccinations, both inside and after risk windows in the cohort analysis (Table 2). These events should therefore be further investigated.

Although most outcomes studied were not significantly associated with vaccination, some (arrhythmia, arthropathy, cerebrovascular events, encephalomyelitis and meningitis, Guillain-Barré syndrome, IgA vasculitis, idiopathic thrombocytopenic purpura, and multisystem inflammatory syndrome in children [MIS-C]) cannot be ruled out due to rarity. We observed no arthropathy cases. Arthropathy is rare and may develop slowly in adolescents, necessitating longer follow-up. Post-vaccination IgA vasculitis has been proposed^34,35^, but we observed no events inside the risk windows in our study. Lai et al. reported no association and few or no cases of meningoencephalitis^12^, and idiopathic thrombocytopenia, Guillain-Barré syndrome and MIS-C^12^, as do Copland et al.^11^. MIS-C has been estimated at 1/1,000,000 vaccinees^36^, which makes our study underpowered. Other studies generally report few events and null associations^11,15,19–21,31,32,37,38^..

## Limitations

Our main limitation was rare outcomes leading to unreliable estimates. Results from small cell sizes should be interpreted with great caution. There might be confounding by indication, as vaccinated and unvaccinated subjects might differ in health status and health-seeking behavior, e.g., lower threshold for medical consultation among vaccinees. Conversely, there might be a healthy-vaccinee effect, where unwell subjects forego vaccination, or an opposite effect where families with healthy children refuse vaccination. Lifestyle-related and societal changes in physical activity, diet, and pandemic restrictions during follow-up might have been present, but adolescents were vaccinated when restrictions were few. Media attention might introduce vigilance/notoriety bias. Including only hospital diagnoses likely limited this. We used unvaccinated person-time as the reference, which could lead to IRR overestimation if the outcome rate was lower during pre-vaccination time. If this is suspected, the risk window IRR could be compared to the IRR post-risk window, assuming no association after the risk windows. No adjustment for multiple testing was done, but we investigated predefined, probable outcomes and our main results would be statistically significant after Bonferroni correction (with α = 0.0029). Unmeasured confounding, misclassifications, and measurement errors, such as undocumented SARS-CoV-2 infection, diagnosis date lagging after the “true” pathogenesis, or miscoding of diagnoses as these are unvalidated and can be unreliable cannot be disregarded. Such occurrences are rare and should be randomly distributed between cases and controls. Most results were congruent when comparing the two analytical methods. Estimates that reached statistical significance in one method, but not the other method, still had overlapping 95%CIs. Yet, these discrepancies could reflect methodological issues such as the underlying method assumptions not being met. The risk windows might be inappropriate, with some estimates increased post-risk window. This could indicate that risk windows were too brief, or a general IR increase over time. The SCCS preexposure risk period for second-dose vaccination might fall inside the first-dose risk window. E.g., if first and second doses were administered within 28 days and the risk window was 28 days, the second-dose 14-day preexposure period would shorten the first-dose risk window to 14 days. This was only relevant for 18–19-year-olds, with recommended 6–12 weeks minimum dose interval.

## Conclusions

The number of observed outcomes and statistically significant associations were generally low, with some exceptions. More adolescent studies are necessary to explore potential age-specific AEFIs, especially in relation to new mRNA vaccines or boosters.

## Methods

We used the Norwegian Emergency Preparedness Register for COVID-19 (BeredtC19)^39^. BeredtC19 includes nation-wide individual-level data on demographics, SARS-CoV-2 infections and vaccinations, and diagnostic codes for the outcomes studied from The Norwegian Registry of Primary Health Care and The Norwegian Patient Registry, covering primary and specialist health services, respectively, linkable by unique identity numbers. These data are linked to reimbursement with a generally high level of completeness^40^. For details, see Supplementary Materials.

The declaration of Helsinki was followed. Institutional board review was conducted by the BeredtC19 steering committee at the Norwegian Institute of Public Health. The study was approved by the Regional Committee for Medical and Health Research Ethics South-East Norway (REK Sør-Øst A/ref.122745), which granted exemption for individual consent. Individual consent was not applicable as the study was based on routinely collected, anonymized registry data.

### Outcomes

We identified 17 outcomes of interest following SARS-CoV-2 vaccination based on vaccine trials, Norwegian surveillance, and reported adverse events. Outcome-specific risk windows were based on recommendations from the World Health Organization, European Medicines Agency, and Brighton Collaboration^41–43^. Table 3 lists outcomes, diagnosis codes and risk windows. As AEFI identification is based on diagnostic codes in our study, spontaneous reports or medically unattended AEFIs are not included, only hospital-diagnosed AEFIs (except Herpes Zoster and death). Outcomes were not reviewed or validated in patient charts. Death was defined as all-cause mortality, including any ICD-10 code registered as underlying cause of death.

### Exposures

Exposures were first- and second-dose tozinameran or elasomeran vaccinations vs. unvaccinated. National vaccination recommendations differed across age groups; 12–15-year-olds (born 2006–2009) were offered the first dose September 2021 and the second dose January 2022, whereas 16–17-year-olds (born 2004–2005) and 18–19-year-olds (born 2002–2003) were recommended two doses with intervals of 8–12 and 6–12 weeks, respectively.

### Study sample

496,432 adolescents (born 2002–2009), residing in Norway (since January 1, 2017, or earlier), unvaccinated when age-specific vaccination waves started: September 6, 2021 (12–15 years), August 23, 2021 (16–17 years), and April 5, 2021 (18–19 years).

### Statistical analysis

We used two designs to compare outcome-specific incidence rates between vaccinated and unvaccinated subjects. The primary analysis was a cohort analyzed using Poisson regression. The secondary analysis was a SCCS method. Follow-up started on the first day of the respective age-group vaccination wave. In each analysis, we excluded subjects with the outcome in question in the four years before follow-up began (outcome data started January 1, 2017). Censoring events were non-mRNA vaccination, third-dose vaccination, emigration, death, or end-of-study (September 30, 2022). Censoring at death can introduce competing risk bias but we assumed minimal impact, due to short risk windows and few deaths in adolescents. We used two-sided tests. Analysis was done in Stata (Release 17) and R (version 4.2.0). Missing covariate data was coded into an own category (“unknown”).

**Fig. 2:**
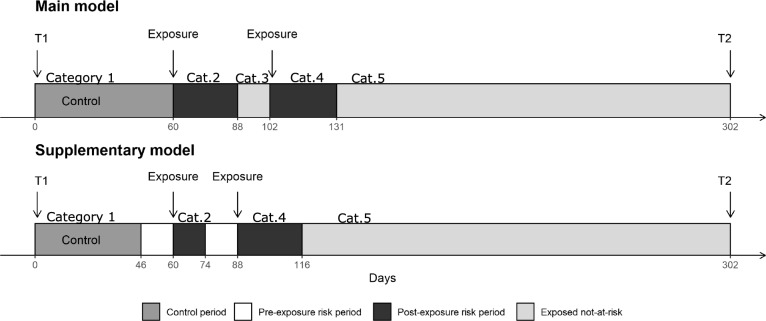
Hypothetical follow-up period in the self-controlled case series analysis based on the main model (upper panel) and the supplementary model with a 14-day preexposure risk period (lower panel). The postexposure risk period is defined by the outcome-specific risk windows. The different time categories used as exposures are marked as category 1 – category 5.

We defined a time-varying five-level categorical exposure variable for subjects’ current mRNA vaccination status: 1) unvaccinated (reference period/group, which includes both never-vaccinated and pre-vaccination person-time among vaccinees), 2) vaccinated with *first* dose *inside* the risk window, 3) vaccinated with *first* dose *after* the risk window, 4) vaccinated with *second* dose *inside* the risk window, and 5) vaccinated with *second* dose *after* the risk window (Fig. 2). Subjects receiving a second dose while still inside the first dose risk window were classified as category 4. Our primary interest was outcomes inside the risk windows (categories 2 and 4). Post-risk window time was not included in the reference/unvaccinated time period but were separate exposure periods to investigate potential long-term associations and if risk windows were too narrow.

### Poisson regression

In the cohort analysis, we applied Poisson regression to estimate incidence rates (IRs). IR ratios (IRRs), with 95% confidence intervals (CIs), of each outcome were estimated for subjects after first- and second-dose vaccinations (compared to unvaccinated). Subjects were followed until the studied outcome or censoring, whichever occurred first. We adjusted for sex, age (12–15, 16–17, or 18–19 years), health region (North, Central, West, or South-East Norway), and preexisting risk conditions (see Supplemental Table 6 for definitions, coded as none vs. any)^1^. Since infection rates and outcomes may exhibit seasonality, we adjusted for a time-varying categorical variable for calendar period (April–June 2021, July–September 2021, October–December 2021, January–March 2022, April–June 2022, and July–September 2022).

### Self-controlled case series analysis

For each outcome, we conducted an SCCS analysis to estimate IRRs, with 95% CIs, for time periods following first- and second-dose vaccinations compared to the unexposed control period, i.e., follow-up time before first-dose vaccination (Fig. 2), as described by Whitaker et al.^16,17^ The cases were followed until censoring. We adjusted for seasonality (January–March, April–June, July–September, or October–December), but not age, as it was considered time-invariant in the relatively short study period.

### Sensitivity analyses

Three sensitivity analyses were done to validate study results.To assess AEFI incidence in subjects without reported infection, we conducted a sensitivity analysis with SARS-CoV-2 infection as a censoring event in the Poisson regression. To explore potential age-group differences we ran the Poisson regression stratified by age-group (12–15, 16–17, or 18–19 years). To investigate potential healthy vaccinee effects in the SCCS analysis, we conducted outcome-specific sensitivity analyses where we excluded 14-day pre-vaccination periods from analysis^16^, and visually inspected exposure-centered plots to assess potential violation of the assumption of outcomes not affecting future vaccination. Lastly, to investigate if the risk window used for acute appendicitis was too brief, we did a post-hoc data-driven analysis using Poisson regression with risk windows of 28, 42 and 56 days.

## Supplementary Information

Supplementary Information.

## Data Availability

Due to the nature of the Emergency register, the authors do not have permission to share individual-level data. Data are only accessible to authorized researchers pending ethical approval and successful application to http:/www.helsedata.no, managed by the Norwegian Institute of Public Health, which can be contacted by a form on the website or at service@helsedata.no.

## Abbreviations

SARS-CoV-2: Severe acute respiratory syndrome coronavirus 2
SCCS: Self-controlled case series
IRR: Incidence rate ratio
ICD-10: International classification of diseases 10th revision
ICPC-2: International classification of primary care 2nd edition

## Code availability

Scripts used are available upon reasonable request to the corresponding author.
