# Supplementary material for: Adverse events following SARS-CoV-2 mRNA vaccination in norwegian adolescents
Source: Sci Rep. 2026 Mar 27;16:10878. doi: 10.1038/s41598-026-45261-2 (PMC13039709; doi:10.1038/s41598-026-45261-2)
Supplement: Supplementary file 1 — Supplementary Information. [file 41598_2026_45261_MOESM1_ESM.docx]

Supplemental Material to:

**Adverse Events Following SARS-CoV-2 mRNA Vaccination in Adolescents:**

**A Norwegian Nationwide Study**

Vilde Bergstad Larsen^1^, M.Sc; Nina Gunnes^2,3^, Ph.D; Jon Michael Gran^4, 5^,Ph.D; Jesper Dahl^5^, MD, Ph.D; Håkon Bøås^5^, Ph.D; Sara Viksmoen Watle^5^, MD, Ph.D; Jacob Dag Berild^5^, MD; Margrethe Greve-Isdahl^5^, MD; Ketil Størdal^6^,MD, Ph.D; Hanne Løvdal Gulseth^2^, MD, Ph.D; Øystein Karlstad^2^,Ph.D; Paz Lopez-Doriga Ruiz^2,7^, MD, Ph.D; German Tapia^2^,Ph.D

^1^Division for Health Services, Norwegian Institute of Public Health, Oslo, Norway

^2^Division of Mental and Physical Health, Norwegian Institute of Public Health, Oslo, Norway

^3^Norwegian Research Centre for Women’s Health, Oslo University Hospital, Oslo, Norway

^4^Oslo Centre for Biostatistics and Epidemiology, Department of Biostatistics, University of Oslo, Oslo, Norway

^5^Department of Infection Control and Vaccines, Norwegian Institute of Public Health, Oslo, Norway

^6^Department of Pediatric Research, Institute of Clinical Medicine, University of Oslo, and Oslo University Hospital, Oslo, Norway

^7^Institute of Community Health and Global Medicine, University of Oslo, Oslo, Norway

Corresponding author: German Tapia, Norwegian Institute of Public Health, Marcus Thranes gate 6, 0473 Oslo, Norway; [german.tapia@fhi.no](mailto:german.tapia@fhi.no)

Contents

[**Supplementary Materials** 3](#_Toc220084527)

[***Short description of Norwegian Healthcare*** 3](#_Toc220084528)

[**Data Sources in Beredt C19** 4](#_Toc220084529)

[**National Population Register** 4](#_Toc220084530)

[**Norwegian Surveillance System for Communicable Diseases** 4](#_Toc220084531)

[**Norwegian Immunisation Registry** 4](#_Toc220084532)

[**Norwegian Registry of Primary Health Care** 5](#_Toc220084533)

[**Norwegian Patient Registry** 5](#_Toc220084534)

[**Norwegian Cause of Death Registry** 5](#_Toc220084535)

[*Supplementary Table 1: Expected and excess events* 11](#_Toc220084536)

[*Supplementary Table 2: Analysis with SARS-CoV-2 infection as censoring event.* 14](#_Toc220084537)

[*Supplementary Table 3: Age-stratified results.* 19](#_Toc220084538)

[*Supplementary Table 4: SCCS results.* 41](#_Toc220084539)

[*Supplementary Table 5: Overview of earlier studies.* 51](#_Toc220084540)

[*Supplementary Table 6: Preexisting risk conditions codes used for adjustment.* 93](#_Toc220084541)

# **Supplementary Materials**

This section includes additional information about the current study.

# ***Short description of Norwegian Healthcare***

Norway has publicly funded universal health care, with almost all hospitals being state-owned and funded, and the few private hospitals also publicly funded. Children below 17 years of age and some selected groups are provided free healthcare, while other citizens pay small user fees (for e.g consultations and medications). Still, hospitalizations (and hospitalization-related costs) are free of charge for all citizens, and all immediate healthcare costs for emergency room admissions are publicly covered.

The central government oversees and finance specialized health services through four regional health trusts (South-east, Central, West and North), which cover their respective regional health regions. Briefly, the South-East health region has the largest population and most urban centers, followed by the West, the Central and lastly the North health region which has the smallest population/urban centers. One of the purposes of state ownership of the regional health trusts is to achieve good and equal specialist care to all citizens at need, regardless of age, gender, place of residence, economical status and ethnic background. Data from these health trusts are registered in the Norwegian Patient Registry. Primary care services, such as general practitioners and primary care emergency clinics, are organized and financed by municipal authorities with reimbursements from these registered in the Norwegian Registry of Primary Health Care (described below). Registering claims in the Norwegian Registry of Primary Health Care or Norwegian Patient Registry is mandatory, and necessary to receive reimbursement/funding.

## **Data Sources in Beredt C19**

Beredt C19 was established in April 2020 by the Norwegian Institute of Public Health to monitor infections, vaccinations, and use of health services in Norway during the COVID-19 pandemic (<https://www.fhi.no/en/id/infectious-diseases/coronavirus/emergency-preparedness-register-for-covid-19/>). The register consists of historical and real-time data from various nationwide electronic health registers and administrative databases to which all reporting is mandatory, thereby covering the entire population in Norway. Data sources in Beredt C19 relevant for the current study are described below.

### **National Population Register**

The National Population Register (<https://www.skatteetaten.no/en/person/national-registry/>) provided information on date and place of birth, sex, date of immigration, county of residence, date of emigration, date of death, and residential status (resident, emigrated, or deceased). The register is updated weekly in Beredt C19.

### **Norwegian Surveillance System for Communicable Diseases**

Information on laboratory-confirmed SARS-CoV-2 infection dates was provided by the Norwegian Surveillance System for Communicable Diseases (MSIS), which is run by the Norwegian Institute of Public Health. All laboratories are legally required to report date of testing and test results to the MSIS. The register was updated daily in Beredt C19.

### **Norwegian Immunisation Registry**

The Norwegian Immunisation Registry (SYSVAK) provided information on SARS-CoV-2 vaccination dates and vaccine types. SYSVAK is run by the Norwegian Institute of Health and updated daily in Beredt C19.

### **Norwegian Registry of Primary Health Care**

The Norwegian Registry of Primary Health Care is based on government reimbursement claims from the primary health services in Norway, including publicly funded general practitioners and primary care emergency clinics. Diagnoses are coded using the International Classification of Primary Care, Second Edition (ICPC-2). The register is updated daily in Beredt C19. In the current study, it provided information on date of diagnosis of herpes zoster (ICPC-2 code S70).

### **Norwegian Patient Registry**

The Norwegian Patient Registry (NPR) collects data from the specialist health services in Norway, which includes all government-owned hospitals and outpatient clinics. Diagnoses are coded using the International Statistical Classification of Diseases and Related Health Problems, Tenth Revision (ICD-10). Reporting to the NPR forms the basis for government reimbursements to the specialist health services in Norway. Together with the Norwegian Registry of Primary Health Care, the NPR covers all government-funded health care in Norway. The register is updated daily in Beredt C19. It provided information on dates of all hospital admissions/discharges and outpatient visits and the corresponding diagnoses. The completeness of unique national identity numbers has been reported to be 99.6% in 2018, and the data from NPR are regularly validated/compared against medical quality registers, with accordance for most conditions ranging from 81%-97%. ^1^

### **Norwegian Cause of Death Registry**

Information on county of residence for deceased subjects was retrieved from the Norwegian Cause of Death Registry, as home address is deleted from the National Population Register upon death. We also used the Norwegian Cause of Death Registry to ascertain whether any subjects were registered with vaccine-associated death (ICD-10 code U12.9). The register is run by the Norwegian Institute of Public Health and updated weekly in Beredt C19.

**References**:
1. Bakken IJ, Ariansen AMS, Knudsen GP, Johansen KI, Vollset SE. The Norwegian Patient Registry and the Norwegian Registry for Primary Health Care: Research potential of two nationwide health-care registries. *Scand J Public Health*. 2020;48(1):49-55.

**Short overview on existing literature.**

There is a seemingly overwhelming number of studies evaluating adverse effects of mRNA COVID-19 vaccines, particularly on myocarditis and pericarditis, but there are few papers truly relevant for other adverse events in adolescents following mRNA vaccination in the general population. By doing a literature search (not a systematic review) using the query “mrna AND vaccine AND covid AND (adolescent OR adolescents) and (safety OR ((adverse OR side) AND (effect OR effects)))” in PubMed (https://pubmed.ncbi.nlm.nih.gov/), we found 422 papers (by 23 January 2025) that were scoped and broadly grouped (presented as a table in Supplemental Table 5) into twelve groups:

1. Other topics (e.g Position statements, Editorials, cost-benefit analysis, qualitative studies, studies investigating other vaccines, n=94)
2. Vaccine effectiveness studies (n=23)
3. Descriptive studies reporting i.e demographics or clinical characteristics, lab-values (e.g antibody titers, MRI data, demographics), but not reporting risk of adverse events (n=74)
4. Studies in wrong age groups (e.g 16-24 years, 0-40 years, ≥18 years, n=78)
5. Studies or safety reports without suitable/comparable comparator, lacking individual-level data or between-group comparisons (e.g male vs female, vector vs mRNA vaccines, n=26)
6. Case report or case series (n=41)
7. Study in small, selected group (e.g cancer patients, people with inborn errors of immunity, n=24)
8. Reviews and meta-analyses (n=37)
9. Studies mainly reporting local and systemic reactions (e.g injection site pain, nausea, fever, chills, n=4)
10. Clinical Trials (n=9)
11. Studies investigating one outcome (n=8)
12. Large cohort studies with individual-level data investigating several outcomes (n=5).

We consider groups 8, 10 ,11 and 12 the most relevant for the present topic. Groups 1-3 are not comparable with the present study as they are not reporting risks of AEFI following mRNA vaccination (or are on vaccines not used in our study). Group 4 is not comparable with the present study as we investigate AEFIs in adolescents (12-19-year-olds) and these are mainly in adult participants. Group 5 could be comparable to a small degree, but are usually not, due to lack of a relevant comparator. Between-group comparisons are often not relevant for the research question we have tried to answer. E.g if mRNA vaccines are associated with higher heart disease risk compared to other medications, have more adverse events reported than influenza vaccines, that vaccinated men have higher incidence of myocarditis compared to vaccinated women, or that younger vaccinated individuals have lower risk for heart disease compared to older vaccinated individuals – do not really answer if a suspected adverse event is associated with vaccination, only if there are differences between the groups studied. There is also often a strong selection at play, with different populations given different vaccines (e.g frail elderly are administered mRNA, young healthcare workers get vector vaccines) or administration at different time-points. Some studies simply divide number of adverse events with number of administered doses, use historical controls (which has a strong assumption that there has been no change, except vaccination, from the historical period), or lack comparator at all and only present the number of reported events. Group 6 are often the earliest reports that motivate and are the rationale behind larger cohort studies, but there will always be case reports/series as outcomes can randomly cluster with vaccination, and these are not considered strong evidence. Group 7 are not comparable when studying the general population and are usually greatly underpowered when investigating AEFIs outside local symptoms and systemic reactions, with all but one of these studies in Supplemental Table 5 having less than 4600 participants (median 94, interquartile range 38-454 participants). Group 9 is not comparable as they are often self-reported and our study investigates different outcomes diagnosed at hospital, albeit anaphylaxis and allergic reactions are sometimes included in these studies. Of the single-outcome studies in group 10, there were four on myocarditis (two investigating booster vaccination), one on myocarditis and anaphylaxis (using self-reported data), one on mortality, one on thyroid disease and one on gynecological well-being. The five (of 422) studies in group 12 are Lai et al. (PMID 35254219), Nordström et al. (PMID 36802397), Dorajoo et al. (PMID 38130887), Rudan et al. (PMID 36189425) and Copland et al. (PMID 38802362). Nordström et al. has studied 30 hospitalization outcomes in Sweden (all-cause hospitalization, gastroenteritis, sepsis, erysipelas, bacterial infection unspecified, mononucleosis, virus infection unspecified, chronic lymphocytic leukemia, iron deficiency anemia, thrombocytopenia, agranulocytosis, alcohol dependency, depressive episode, anxiety state unspecified, allergy or anaphylactic shock, anorexia nervosa, epilepsy, otitis media, myocarditis, pericarditis, sinusitis, tonsilitis, chronic tonsillitis, upper respiratory infection, pneumonia, peritonsillar abscess, appendicitis, Crohn’s disease, cutaneous abscess, nephritis, traumatic brain injury) and report lower risk of hospitalization for sepsis, thrombocytopenia, alcohol dependency, peritonsillar abscess and Crohn`s following mRNA vaccination. Nordström et al. does not use risk windows, instead following vaccinated individuals from vaccination until censoring or study end, which makes their results not comparable with ours as the present study investigates adverse events in relatively short time windows after vaccination. Rudan et al., Dorajoo et al., Lai et al. and Copland et al. are more directly comparable, and their results are discussed in more detail in the main manuscript. Still, differences in age-distributions, risk windows, statistical analysis and diagnostic coding in addition to national differences in policy, vaccine coverage, vaccine dose intervals, infection and vaccination rates during the COVID-19 pandemic make comparisons complex.

*Supplementary Table 1: Expected and excess events (with associated 95% confidence interval) of 17 different outcomes based on Poisson regression of 477,097 adolescents in Norway aged 12–19 years at the end of 2021 and unvaccinated against SARS-CoV-2 and previously uninfected with SARS-CoV-2 at the beginning of follow-up. Subjects were followed from the beginning of the wave of vaccination^1^ of their age group until the outcome in question, non-mRNA SARS-CoV-2 vaccination, third-dose SARS-CoV-2 vaccination, SARS-CoV-2 infection, emigration, death, or end of study on September 30, 2022, whichever occurred first. To ensure data privacy, numbers between 1 and 4 have been suppressed and are denoted by “< 5”. As a result, some of the totals have been suppressed as well to avoid revealing small numbers that have been suppressed.*

| Outcome | Risk window | Vaccination status | Subjects | Incidence rate | Events | Expected Events | Excess events (95%CI) |
| --- | --- | --- | --- | --- | --- | --- | --- |
| Acute appendicitis | 14 days | Overall | 492,360 | 220.2 | 1,185 |  |  |
|  |  | Unvaccinated | 491,589 | 181.9 | 267 | 267 | 0.00 (-45.29 to 45.29) |
|  |  | Vaccinated with 1st dose, inside risk window | 405,689 | 231.5 | 36 | 28.28 | 7.72 (-7.99 to 23.44) |
|  |  | Vaccinated with 1st dose, outside risk window | 405,588 | 235.0 | 495 | 383.1 | 111.89 (53.81 to 169.97) |
|  |  | Vaccinated with 2nd dose, inside risk window | 225,523 | 243.0 | 21 | 15.72 | 5.28 (-6.59 to 17.16) |
|  |  | Vaccinated with 2nd dose, outside risk window | 225,341 | 233.9 | 366 | 284.6 | 81.38 (31.39 to 131.37) |
| Anaphylactic reaction | 2 days | Overall | 496,226 | 10.31 | < 61 |  |  |
|  |  | Unvaccinated | 495,443 | 8.107 | 12 | 12 | 0.00 (-9.60 to 9.60) |
|  |  | Vaccinated with 1st dose, inside risk window | 409,035 | 44.65 | < 5 | ND | ND |
|  |  | Vaccinated with 1st dose, outside risk window | 409,021 | 11.51 | 26 | 18.32 | 7.68 (-5.37 to 20.73) |
|  |  | Vaccinated with 2nd dose, inside risk window | 227,541 | 80.26 | < 5 | ND | ND |
|  |  | Vaccinated with 2nd dose, outside risk window | 227,507 | 9.666 | 16 | 13.42 | 2.58 (-8.05 to 13.21) |
| Arrhythmia | 28 days | Overall | 495,835 | 24.88 | 135 |  |  |
|  |  | Unvaccinated | 495,054 | 26.37 | 39 | 39 | 0.00 (-17.31 to 17.31) |
|  |  | Vaccinated with 1st dose, inside risk window | 408676 | 12.80 | < 5 | ND | ND |
|  |  | Vaccinated with 1st dose, outside risk window | 397465 | 21.85 | 43 | 51.90 | -8.90 (-27.99 to 10.20) |
|  |  | Vaccinated with 2nd dose, inside risk window | 227291 | 5.744 | < 5 | ND | ND |
|  |  | Vaccinated with 2nd dose, outside risk window | 226911 | 32.18 | 48 | 39.34 | 8.66 (-9.65 to 26.98) |
| Arthropathy | 42 days | Overall | 496414 | 0 | 0 |  |  |
|  |  | Unvaccinated | 495631 | 0 | 0 | 0 | - |
|  |  | Vaccinated with 1st dose, inside risk window | 409206 | 0 | 0 | 0 | - |
|  |  | Vaccinated with 1st dose, outside risk window | 362098 | 0 | 0 | 0 | - |
|  |  | Vaccinated with 2nd dose, inside risk window | 227662 | 0 | 0 | 0 | - |
|  |  | Vaccinated with 2nd dose, outside risk window | 227093 | 0 | 0 | 0 | - |
| Cerebrovascular events | 28 days | Overall | 496307 | 5.708 | < 33 |  |  |
|  |  | Unvaccinated | 495524 | 6.755 | 10 | 10 | 0.00 (-8.77 to 8.77) |
|  |  | Vaccinated with 1st dose, inside risk window | 409109 | 9.588 | < 5 | ND | ND |
|  |  | Vaccinated with 1st dose, outside risk window | 397881 | 6.092 | 12 | 13.31 | -1.31 (-11.17 to 8.55) |
|  |  | Vaccinated with 2nd dose, inside risk window | 227587 | 0 | 0 | 1.180 | -1.18 (-3.30 to 0.95) |
|  |  | Vaccinated with 2nd dose, outside risk window | 227208 | 4.017 | 6 | 10.09 | -4.09 (-11.95 to 3.77) |
| Death (all-cause mortality) | 28 days | Overall | 496414 | 20.06 | 109 |  |  |
|  |  | Unvaccinated | 495631 | 27.69 | 41 | 41 | 0.00 (-17.75 to 17.75) |
|  |  | Vaccinated with 1st dose, inside risk window | 409206 | 22.37 | 7 | 8.670 | -1.67 (-9.42 to 6.09) |
|  |  | Vaccinated with 1st dose, outside risk window | 397975 | 11.67 | 23 | 54.55 | -31.55 (-48.81 to -14.29) |
|  |  | Vaccinated with 2nd dose, inside risk window | 227662 | 28.67 | < 5 | ND | ND |
|  |  | Vaccinated with 2nd dose, outside risk window | 227283 | 22.08 | 33 | 41.38 | -8.38 (-25.28 to 8.53) |
| Encephalomyelitis and meningitis | 28 days | Overall | 496349 | 2.577 | 14 |  |  |
|  |  | Unvaccinated | 495566 | 2.702 | < 5 | ND | ND |
|  |  | Vaccinated with 1st dose, inside risk window | 409153 | 0 | 0 | 0.850 | -0.85 (-2.65 to 0.96) |
|  |  | Vaccinated with 1st dose, outside risk window | 397924 | 2.538 | < 5 | ND | ND |
|  |  | Vaccinated with 2nd dose, inside risk window | 227629 | 5.735 | < 5 | ND | ND |
|  |  | Vaccinated with 2nd dose, outside risk window | 227250 | 2.677 | < 5 | ND | ND |
| Epilepsy and convulsions | 28 days | Overall | 491327 | 114.8 | 617 |  |  |
|  |  | Unvaccinated | 490551 | 112.7 | 165 | 165 | 0.00 (-35.61 to 35.61) |
|  |  | Vaccinated with 1st dose, inside risk window | 405001 | 93.62 | 29 | 34.90 | -5.90 (-21.57 to 9.77) |
|  |  | Vaccinated with 1st dose, outside risk window | 393882 | 115.4 | 225 | 219.6 | 5.37 (-35.96 to 46.70) |
|  |  | Vaccinated with 2nd dose, inside risk window | 225243 | 173.9 | 30 | 19.44 | 10.56 (-3.22 to 24.34) |
|  |  | Vaccinated with 2nd dose, outside risk window | 224841 | 113.7 | 168 | 166.5 | 1.50 (-34.35 to 37.35) |
| Facial nerve palsy | 28 days | Overall | 496027 | 16.77 | 91 |  |  |
|  |  | Unvaccinated | 495244 | 22.98 | 34 | 34 | 0.00 (-16.16 to 16.16) |
|  |  | Vaccinated with 1st dose, inside risk window | 408885 | 12.79 | < 5 | ND | ND |
|  |  | Vaccinated with 1st dose, outside risk window | 397659 | 10.16 | 20 | 45.24 | -25.24 (-41.08 to -9.41) |
|  |  | Vaccinated with 2nd dose, inside risk window | 227469 | 11.48 | < 5 | ND | ND |
|  |  | Vaccinated with 2nd dose, outside risk window | 227088 | 20.77 | 31 | 34.31 | -3.31 (-19.15 to 12.53) |
| Guillain-Barré syndrome | 42 days | Overall | 496401 | 1.105 | 6 |  |  |
|  |  | Unvaccinated | 495618 | 0 | 0 | 0 | - |
|  |  | Vaccinated with 1st dose, inside risk window | 409193 | 0 | 0 | 0 | - |
|  |  | Vaccinated with 1st dose, outside risk window | 362086 | 1.097 | <5 | ND | ND |
|  |  | Vaccinated with 2nd dose, inside risk window | 227652 | 0 | 0 | 0 | - |
|  |  | Vaccinated with 2nd dose, outside risk window | 227083 | 2.843 | <5 | ND | ND |
| IgA vasculitis | 42 days | Overall | 496343 | 2.578 | < 17 |  |  |
|  |  | Unvaccinated | 495560 | 1.351 | < 5 | ND | ND |
|  |  | Vaccinated with 1st dose, inside risk window | 409149 | 0 | 0 | 0.620 | -0.62 (-2.17 to 0.92) |
|  |  | Vaccinated with 1st dose, outside risk window | 362048 | 3.842 | 7 | 2.460 | 4.54 (-1.49 to 10.57) |
|  |  | Vaccinated with 2nd dose, inside risk window | 227633 | 0 | 0 | 0.350 | -0.35 (-1.52 to 0.81) |
|  |  | Vaccinated with 2nd dose, outside risk window | 227064 | 3.554 | < 5 | ND | ND |
| Herpes zoster | 28 days | Overall | 496181 | 14.73 | 80 |  |  |
|  |  | Unvaccinated | 495398 | 12.16 | 18 | 18 | 0.00 (-11.76 to 11.76) |
|  |  | Vaccinated with 1st dose, inside risk window | 409001 | 6.393 | < 5 | ND | ND |
|  |  | Vaccinated with 1st dose, outside risk window | 397775 | 14.73 | 29 | 23.95 | 5.05 (-9.21 to 19.31) |
|  |  | Vaccinated with 2nd dose, inside risk window | 227530 | 5.738 | < 5 | ND | ND |
|  |  | Vaccinated with 2nd dose, outside risk window | 227151 | 20.09 | 30 | 18.16 | 11.84 (-1.76 to 25.44) |
| Idiopathic thrombocytopenic purpura | 28 days | Overall | 496289 | 4.419 | 24 |  |  |
|  |  | Unvaccinated | 495506 | 4.054 | 6 | 6 | -0.00 (-6.79 to 6.79) |
|  |  | Vaccinated with 1st dose, inside risk window | 409107 | 6.392 | < 5 | ND | ND |
|  |  | Vaccinated with 1st dose, outside risk window | 397881 | 4.062 | 8 | 7.980 | 0.02 (-7.82 to 7.85) |
|  |  | Vaccinated with 2nd dose, inside risk window | 227605 | 5.736 | < 5 | ND | ND |
|  |  | Vaccinated with 2nd dose, outside risk window | 227227 | 4.686 | 7 | 6.060 | 0.94 (-6.14 to 8.03) |
| Lymphadenopathy | 14 days | Overall | 494138 | 120.5 | 651 |  |  |
|  |  | Unvaccinated | 493360 | 103.2 | 152 | 152 | 0.00 (-34.17 to 34.17) |
|  |  | Vaccinated with 1st dose, inside risk window | 407246 | 96.10 | 15 | 16.10 | -1.10 (-12.03 to 9.83) |
|  |  | Vaccinated with 1st dose, outside risk window | 407166 | 117.7 | 249 | 218.2 | 30.77 (-11.60 to 73.13) |
|  |  | Vaccinated with 2nd dose, inside risk window | 226468 | 253.5 | 22 | 8.950 | 13.05 (2.14 to 23.95) |
|  |  | Vaccinated with 2nd dose, outside risk window | 226284 | 135.5 | 213 | 162.2 | 50.79 (12.83 to 88.76) |
| MIS-C | 42 days | Overall | 496389 | 3.866 | 21 |  |  |
|  |  | Unvaccinated | 495606 | 4.053 | 6 | 6 | 0.00 (-6.79 to 6.79) |
|  |  | Vaccinated with 1st dose, inside risk window | 409187 | 6.513 | < 5 | ND | ND |
|  |  | Vaccinated with 1st dose, outside risk window | 362079 | 4.390 | 8 | 7.390 | 0.61 (-7.07 to 8.30) |
|  |  | Vaccinated with 2nd dose, inside risk window | 227656 | 0 | 0 | 1.060 | -1.06 (-3.08 to 0.96) |
|  |  | Vaccinated with 2nd dose, outside risk window | 227087 | 2.843 | < 5 | ND | ND |
| Myocarditis and pericarditis | 28 days | Overall | 496359 | 12.15 | < 68 |  |  |
|  |  | Unvaccinated | 495576 | 8.781 | 13 | 13 | 0.00 (-9.99 to 9.99) |
|  |  | Vaccinated with 1st dose, inside risk window | 409150 | 9.587 | < 5 | ND | ND |
|  |  | Vaccinated with 1st dose, outside risk window | 397921 | 8.630 | 17 | 17.30 | -0.30 (-11.78 to 11.18) |
|  |  | Vaccinated with 2nd dose, inside risk window | 227609 | 63.09 | 11 | 1.530 | 9.47 (2.53 to 16.41) |
|  |  | Vaccinated with 2nd dose, outside risk window | 227219 | 14.73 | 22 | 13.12 | 8.88 (-2.73 to 20.50) |
| Venous thromboembolic events | 28 days | Overall | 496310 | 12.71 | < 71 |  |  |
|  |  | Unvaccinated | 495527 | 7.431 | 11 | 11 | 0.00 (-9.19 to 9.19) |
|  |  | Vaccinated with 1st dose, inside risk window | 409104 | 15.98 | < 5 | ND | ND |
|  |  | Vaccinated with 1st dose, outside risk window | 397875 | 10.15 | 20 | 14.64 | 5.36 (-6.17 to 16.90) |
|  |  | Vaccinated with 2nd dose, inside risk window | 227564 | 17.21 | < 5 | ND | ND |
|  |  | Vaccinated with 2nd dose, outside risk window | 227182 | 20.09 | 30 | 11.10 | 18.90 (6.34 to 31.47) |

Abbreviations: CI – confidence interval; IRR – incidence rate ratio; mRNA – messenger RNA; ND – not determined; MIS-C – Multisystem Inflammatory Syndrome in Children

^1^12–15-year-olds: September 6, 2021; 16–17-year-olds: August 23, 2021; 18–19-year-olds: April 5, 2021

^2^Per 100,000 person-years

*Supplementary Table 2: Analysis with SARS-CoV-2 infection as censoring event. Crude and adjusted incidence rate ratios of 17 different outcomes between vaccinated and unvaccinated subjects, with associated 95% confidence intervals, based on Poisson regression of 477,097 adolescents in Norway aged 12–19 years at the end of 2021 and unvaccinated against SARS-CoV-2 and previously uninfected with SARS-CoV-2 at the beginning of follow-up. Subjects were followed from the beginning of the wave of vaccination^1^ of their age group until the outcome in question, non-mRNA SARS-CoV-2 vaccination, third-dose SARS-CoV-2 vaccination, SARS-CoV-2 infection, emigration, death, or end of study on September 30, 2022, whichever occurred first. To ensure data privacy, numbers between 1 and 4 have been suppressed and are denoted by “< 5”. As a result, some of the totals have been suppressed as well to avoid revealing small numbers that have been suppressed.*

| Outcome | Risk window | Vaccination status | Number of subjects | Number of events | Incidence rate^2^ | Crude analysis | | |  | | Adjusted analysis^3^ | |  |
| --- | --- | --- | --- | --- | --- | --- | --- | --- | --- | --- | --- | --- | --- |
|  |  |  |  |  |  | IRR | 95% CI | P value | | IRR | | 95% CI | P value |
| Acute appendicitis | 14 days | *Overall* | 472,625 | 802 | 217.77 |  |  |  | |  | |  |  |
|  |  | Unvaccinated (ref.) | 471,868 | 184 | 184.88 | 1 |  |  | | 1 | |  |  |
|  |  | Vaccinated with 1^st^ dose, inside risk window | 388,948 | 34 | 229.38 | 1.24 | 0.86–1.79 | 0.248 | | 1.33 | | 0.90–1.96 | 0.147 |
|  |  | Vaccinated with 1^st^ dose, outside risk window | 384,298 | 300 | 229.31 | 1.24 | 1.03–1.49 | 0.021 | | 1.56 | | 1.25–1.94 | 0.000 |
|  |  | Vaccinated with 2^nd^ dose, inside risk window | 213,406 | 20 | 246.64 | 1.33 | 0.84–2.12 | 0.221 | | 1.42 | | 0.87–2.30 | 0.159 |
|  |  | Vaccinated with 2^nd^ dose, outside risk window | 210,127 | 264 | 229.58 | 1.24 | 1.03–1.50 | 0.024 | | 1.45 | | 1.10–1.90 | 0.008 |
| Anaphylactic reaction | 2 days | *Overall* | 476,333 | 41 | 11.04 |  |  |  | |  | |  |  |
|  |  | Unvaccinated (ref.) | 475,564 | 9 | 8.97 | 1 |  |  | | 1 | |  |  |
|  |  | Vaccinated with 1^st^ dose, inside risk window | 392,153 | < 5 | 46.58 | 5.19 | 0.66–40.98 | 0.118 | | 6.84 | | 0.81–57.81 | 0.077 |
|  |  | Vaccinated with 1^st^ dose, outside risk window | 391,666 | 19 | 13.13 | 1.46 | 0.66–3.23 | 0.347 | | 2.75 | | 1.00–7.56 | 0.049 |
|  |  | Vaccinated with 2^nd^ dose, inside risk window | 215,309 | < 5 | 84.86 | 9.46 | 1.20–74.65 | 0.033 | | 11.02 | | 1.26–96.68 | 0.030 |
|  |  | Vaccinated with 2^nd^ dose, outside risk window | 214,849 | 11 | 8.94 | 1.00 | 0.41–2.40 | 0.993 | | 0.88 | | 0.26–2.95 | 0.830 |
| Arrhythmia | 28 days | *Overall* | 475,961 | 90 | 24.25 |  |  |  | |  | |  |  |
|  |  | Unvaccinated (ref.) | 475,194 | 26 | 25.94 | 1 |  |  | | 1 | |  |  |
|  |  | Vaccinated with 1^st^ dose, inside risk window | 391,814 | < 5 | 10.11 | 0.39 | 0.12–1.29 | 0.122 | | 0.38 | | 0.11–1.31 | 0.126 |
|  |  | Vaccinated with 1^st^ dose, outside risk window | 374,429 | 24 | 20.49 | 0.79 | 0.45–1.38 | 0.405 | | 1.12 | | 0.58–2.16 | 0.726 |
|  |  | Vaccinated with 2^nd^ dose, inside risk window | 215,069 | < 5 | 6.15 | 0.24 | 0.03–1.75 | 0.158 | | 0.20 | | 0.03–1.56 | 0.126 |
|  |  | Vaccinated with 2^nd^ dose, outside risk window | 210,483 | 36 | 33.37 | 1.29 | 0.78–2.13 | 0.328 | | 1.34 | | 0.64–2.82 | 0.438 |
| Arthropathy | 42 days | *Overall* | 476,515 | 0 | 0.00 |  |  |  | |  | |  |  |
|  |  | Unvaccinated (ref.) | 475,746 | 0 | 0.00 | 1 |  |  | | 1 | |  |  |
|  |  | Vaccinated with 1^st^ dose, inside risk window | 392,319 | 0 | 0.00 | ND | ND | ND | | ND | | ND | ND |
|  |  | Vaccinated with 1^st^ dose, outside risk window | 336,587 | 0 | 0.00 | ND | ND | ND | | ND | | ND | ND |
|  |  | Vaccinated with 2^nd^ dose, inside risk window | 215,425 | 0 | 0.00 | ND | ND | ND | | ND | | ND | ND |
|  |  | Vaccinated with 2^nd^ dose, outside risk window | 208,863 | 0 | 0.00 | ND | ND | ND | | ND | | ND | ND |
| Cerebrovascular events | 28 days | *Overall* | 476,409 | 22 | 5.92 |  |  |  | |  | |  |  |
|  |  | Unvaccinated (ref.) | 475,640 | 8 | 7.97 | 1 |  |  | | 1 | |  |  |
|  |  | Vaccinated with 1^st^ dose, inside risk window | 392,224 | < 5 | 10.10 | 1.27 | 0.34–4.77 | 0.727 | | 1.26 | | 0.28–5.58 | 0.764 |
|  |  | Vaccinated with 1^st^ dose, outside risk window | 374,812 | 7 | 5.97 | 0.75 | 0.27–2.07 | 0.577 | | 0.88 | | 0.26–2.98 | 0.838 |
|  |  | Vaccinated with 2^nd^ dose, inside risk window | 215,353 | 0 | 0.00 | ND | ND | ND | | ND | | ND | ND |
|  |  | Vaccinated with 2^nd^ dose, outside risk window | 210,758 | < 5 | 3.70 | 0.46 | 0.14–1.54 | 0.210 | | 0.27 | | 0.06–1.28 | 0.100 |
| Death (all-cause mortality) | 28 days | *Overall* | 476,515 | 90 | 24.22 |  |  |  | |  | |  |  |
|  |  | Unvaccinated (ref.) | 475,746 | 36 | 35.87 | 1 |  |  | | 1 | |  |  |
|  |  | Vaccinated with 1^st^ dose, inside risk window | 392,319 | 7 | 23.56 | 0.66 | 0.29–1.48 | 0.309 | | 0.71 | | 0.29–1.69 | 0.433 |
|  |  | Vaccinated with 1^st^ dose, outside risk window | 374,903 | 18 | 15.35 | 0.43 | 0.24–0.75 | 0.003 | | 0.59 | | 0.31–1.13 | 0.110 |
|  |  | Vaccinated with 2^nd^ dose, inside risk window | 215,425 | 5 | 30.68 | 0.86 | 0.34–2.18 | 0.743 | | 0.69 | | 0.26–1.87 | 0.471 |
|  |  | Vaccinated with 2^nd^ dose, outside risk window | 210,830 | 24 | 22.21 | 0.62 | 0.37–1.04 | 0.069 | | 0.40 | | 0.20–0.78 | 0.007 |
| Encephalomyelitis and meningitis | 28 days | *Overall* | 476,453 | 7 | 1.88 |  |  |  | |  | |  |  |
|  |  | Unvaccinated (ref.) | 475,684 | < 5 | 1.00 | 1 |  |  | | 1 | |  |  |
|  |  | Vaccinated with 1^st^ dose, inside risk window | 392,267 | 0 | 0.00 | ND | ND | ND | | ND | | ND | ND |
|  |  | Vaccinated with 1^st^ dose, outside risk window | 374,854 | < 5 | 1.71 | 1.71 | 0.16–18.88 | 0.661 | | 1.18 | | 0.10–13.79 | 0.895 |
|  |  | Vaccinated with 2^nd^ dose, inside risk window | 215,394 | < 5 | 6.14 | 6.16 | 0.39–98.45 | 0.199 | | 5.98 | | 0.28–126.35 | 0.251 |
|  |  | Vaccinated with 2^nd^ dose, outside risk window | 210,799 | < 5 | 2.78 | 2.79 | 0.29–26.78 | 0.375 | | 1.69 | | 0.11–26.81 | 0.709 |
| Epilepsy and convulsions | 28 days | *Overall* | 471,600 | 454 | 123.56 |  |  |  | |  | |  |  |
|  |  | Unvaccinated (ref.) | 470,838 | 107 | 107.85 | 1 |  |  | | 1 | |  |  |
|  |  | Vaccinated with 1^st^ dose, inside risk window | 388,243 | 29 | 98.63 | 0.91 | 0.61–1.38 | 0.670 | | 1.07 | | 0.69–1.67 | 0.749 |
|  |  | Vaccinated with 1^st^ dose, outside risk window | 370,996 | 157 | 135.46 | 1.26 | 0.98–1.61 | 0.069 | | 1.21 | | 0.91–1.61 | 0.187 |
|  |  | Vaccinated with 2^nd^ dose, inside risk window | 213,099 | 28 | 173.71 | 1.61 | 1.06–2.44 | 0.025 | | 1.65 | | 1.05–2.59 | 0.028 |
|  |  | Vaccinated with 2^nd^ dose, outside risk window | 208,521 | 133 | 124.53 | 1.15 | 0.90–1.49 | 0.268 | | 1.01 | | 0.71–1.44 | 0.944 |
| Facial nerve palsy | 28 days | *Overall* | 476,148 | 57 | 15.35 |  |  |  | |  | |  |  |
|  |  | Unvaccinated (ref.) | 475,379 | 19 | 18.95 | 1 |  |  | | 1 | |  |  |
|  |  | Vaccinated with 1^st^ dose, inside risk window | 392,016 | < 5 | 13.47 | 0.71 | 0.24–2.09 | 0.535 | | 0.65 | | 0.21–2.02 | 0.454 |
|  |  | Vaccinated with 1^st^ dose, outside risk window | 374,613 | 10 | 8.54 | 0.45 | 0.21–0.97 | 0.041 | | 0.47 | | 0.20–1.11 | 0.085 |
|  |  | Vaccinated with 2^nd^ dose, inside risk window | 215,248 | < 5 | 12.28 | 0.65 | 0.15–2.78 | 0.560 | | 0.49 | | 0.11–2.26 | 0.359 |
|  |  | Vaccinated with 2^nd^ dose, outside risk window | 210,657 | 22 | 20.38 | 1.08 | 0.58–1.99 | 0.816 | | 1.02 | | 0.41–2.53 | 0.964 |
| Guillain-Barré syndrome | 42 days | *Overall* | 476,503 | < 5 | 0.81 |  |  |  | |  | |  |  |
|  |  | Unvaccinated (ref.) | 475,734 | 0 | 0.00 | 1 |  |  | | 1 | |  |  |
|  |  | Vaccinated with 1^st^ dose, inside risk window | 392,307 | 0 | 0.00 | ND | ND | ND | | ND | | ND | ND |
|  |  | Vaccinated with 1^st^ dose, outside risk window | 336,577 | 0 | 0.00 | ND | ND | ND | | ND | | ND | ND |
|  |  | Vaccinated with 2^nd^ dose, inside risk window | 215,416 | 0 | 0.00 | ND | ND | ND | | ND | | ND | ND |
|  |  | Vaccinated with 2^nd^ dose, outside risk window | 208,855 | < 5 | 3.00 | ND | ND | ND | | ND | | ND | ND |
| IgA vasculitis | 42 days | *Overall* | 476,444 | 7 | 1.88 |  |  |  | |  | |  |  |
|  |  | Unvaccinated (ref.) | 475,675 | < 5 | 1.00 | 1 |  |  | | 1 | |  |  |
|  |  | Vaccinated with 1^st^ dose, inside risk window | 392,263 | 0 | 0.00 | ND | ND | ND | | ND | | ND | ND |
|  |  | Vaccinated with 1^st^ dose, outside risk window | 336,538 | 5 | 4.84 | 4.85 | 0.57–41.52 | 0.149 | | ND | | ND | ND |
|  |  | Vaccinated with 2^nd^ dose, inside risk window | 215,397 | 0 | 0.00 | ND | ND | ND | | ND | | ND | ND |
|  |  | Vaccinated with 2^nd^ dose, outside risk window | 208,835 | < 5 | 1.00 | 1.00 | 0.06–16.04 | 0.998 | | ND | | ND | ND |
| Herpes zoster | 28 days | *Overall* | 476,297 | 47 | 12.65 |  |  |  | |  | |  |  |
|  |  | Unvaccinated (ref.) | 475,528 | 9 | 8.97 | 1 |  |  | | 1 | |  |  |
|  |  | Vaccinated with 1^st^ dose, inside risk window | 392,127 | < 5 | 6.73 | 0.75 | 0.16–3.47 | 0.714 | | 1.00 | | 0.20–5.09 | 0.997 |
|  |  | Vaccinated with 1^st^ dose, outside risk window | 374,721 | 15 | 12.80 | 1.43 | 0.62–3.26 | 0.399 | | 1.30 | | 0.49–3.42 | 0.598 |
|  |  | Vaccinated with 2^nd^ dose, inside risk window | 215,299 | < 5 | 6.14 | 0.68 | 0.09–5.40 | 0.719 | | 0.82 | | 0.10–6.85 | 0.853 |
|  |  | Vaccinated with 2^nd^ dose, outside risk window | 210,710 | 20 | 18.52 | 2.06 | 0.94–4.53 | 0.071 | | 1.86 | | 0.62–5.56 | 0.265 |
| Idiopathic thrombocytopenic | 28 days | *Overall* | 476,398 | 18 | 4.84 |  |  |  | |  | |  |  |
| purpura |  | Unvaccinated (ref.) | 475,629 | 5 | 4.98 | 1 |  |  | | 1 | |  |  |
|  |  | Vaccinated with 1^st^ dose, inside risk window | 392,227 | < 5 | 6.73 | 1.35 | 0.26–6.96 | 0.719 | | 0.98 | | 0.18–5.44 | 0.983 |
|  |  | Vaccinated with 1^st^ dose, outside risk window | 374,817 | 5 | 4.27 | 0.86 | 0.25–2.96 | 0.806 | | 1.28 | | 0.29–5.71 | 0.748 |
|  |  | Vaccinated with 2^nd^ dose, inside risk window | 215,372 | < 5 | 6.14 | 1.23 | 0.14–10.54 | 0.849 | | 1.16 | | 0.11–11.87 | 0.901 |
|  |  | Vaccinated with 2^nd^ dose, outside risk window | 210,779 | 5 | 4.63 | 0.93 | 0.27–3.21 | 0.907 | | 2.18 | | 0.37–12.99 | 0.391 |
| Lymphadenopathy | 14 days | *Overall* | 474,345 | 438 | 118.44 |  |  |  | |  | |  |  |
|  |  | Unvaccinated (ref.) | 473,581 | 97 | 97.10 | 1 |  |  | | 1 | |  |  |
|  |  | Vaccinated with 1^st^ dose, inside risk window | 390,457 | 15 | 100.80 | 1.04 | 0.60–1.79 | 0.893 | | 1.23 | | 0.70–2.18 | 0.474 |
|  |  | Vaccinated with 1^st^ dose, outside risk window | 385,807 | 157 | 119.52 | 1.23 | 0.96–1.59 | 0.108 | | 1.29 | | 0.96–1.73 | 0.094 |
|  |  | Vaccinated with 2^nd^ dose, inside risk window | 214,303 | 20 | 245.61 | 2.53 | 1.56–4.09 | 0.000 | | 2.73 | | 1.63–4.58 | 0.000 |
|  |  | Vaccinated with 2^nd^ dose, outside risk window | 211,014 | 149 | 128.98 | 1.33 | 1.03–1.72 | 0.030 | | 1.40 | | 0.98–2.01 | 0.068 |
| Multisystem inflammatory | 42 days | *Overall* | 476,500 | 6 | 1.61 |  |  |  | |  | |  |  |
| syndrome in children |  | Unvaccinated (ref.) | 475,731 | < 5 | 1.00 | 1 |  |  | | 1 | |  |  |
|  |  | Vaccinated with 1^st^ dose, inside risk window | 392,305 | < 5 | 2.30 | 2.30 | 0.14–36.83 | 0.555 | | 1.61 | | 0.09–28.39 | 0.744 |
|  |  | Vaccinated with 1^st^ dose, outside risk window | 336,576 | < 5 | 1.93 | 1.94 | 0.18–21.41 | 0.588 | | 3.26 | | 0.11–93.03 | 0.490 |
|  |  | Vaccinated with 2^nd^ dose, inside risk window | 215,420 | 0 | 0.00 | ND | ND | ND | | ND | | ND | ND |
|  |  | Vaccinated with 2^nd^ dose, outside risk window | 208,858 | < 5 | 2.00 | 2.01 | 0.18–22.13 | 0.570 | | 3.73 | | 0.08–169.70 | 0.499 |
| Myocarditis and pericarditis | 28 days | *Overall* | 476,463 | < 53 | 13.73 |  |  |  | |  | |  |  |
|  |  | Unvaccinated (ref.) | 475,694 | 9 | 8.97 | 1 |  |  | | 1 | |  |  |
|  |  | Vaccinated with 1^st^ dose, inside risk window | 392,268 | < 5 | 10.10 | 1.13 | 0.30–4.16 | 0.859 | | 1.57 | | 0.35–7.04 | 0.557 |
|  |  | Vaccinated with 1^st^ dose, outside risk window | 374,855 | 13 | 11.09 | 1.24 | 0.53–2.89 | 0.625 | | 4.84 | | 1.42–16.47 | 0.012 |
|  |  | Vaccinated with 2^nd^ dose, inside risk window | 215,375 | 11 | 67.52 | 7.53 | 3.12–18.17 | 0.000 | | 9.19 | | 2.69–31.45 | 0.000 |
|  |  | Vaccinated with 2^nd^ dose, outside risk window | 210,769 | 15 | 13.89 | 1.55 | 0.68–3.54 | 0.300 | | 3.16 | | 0.79–12.64 | 0.104 |
| Venous thromboembolic events | 28 days | *Overall* | 476,415 | < 54 | 14.00 |  |  |  | |  | |  |  |
|  |  | Unvaccinated (ref.) | 475,646 | 9 | 8.97 | 1 |  |  | | 1 | |  |  |
|  |  | Vaccinated with 1^st^ dose, inside risk window | 392,221 | 5 | 16.83 | 1.88 | 0.63–5.60 | 0.259 | | 2.40 | | 0.67–8.62 | 0.180 |
|  |  | Vaccinated with 1^st^ dose, outside risk window | 374,811 | 13 | 11.09 | 1.24 | 0.53–2.89 | 0.625 | | 2.67 | | 0.85–8.41 | 0.094 |
|  |  | Vaccinated with 2^nd^ dose, inside risk window | 215,335 | < 5 | 18.42 | 2.05 | 0.56–7.58 | 0.281 | | 2.17 | | 0.48–9.73 | 0.313 |
|  |  | Vaccinated with 2^nd^ dose, outside risk window | 210,737 | 22 | 20.37 | 2.27 | 1.05–4.93 | 0.038 | | 2.53 | | 0.72–8.85 | 0.147 |

Abbreviations: CI – confidence interval; IRR – incidence rate ratio; mRNA – messenger RNA; ND – not determined

^1^12–15-year-olds: September 6, 2021; 16–17-year-olds: August 23, 2021; 18–19-year-olds: April 5, 2021

^2^Per 100,000 person-years

^3^Adjustment for sex (male or female), attained age by the end of 2021 (12–15 years, 16–17 years, or 18–19 years), health region (North Norway, Central Norway, West Norway, or South-East Norway), and risk group (no or yes) as baseline covariates and three-month calendar period (April–June 2021, July–September 2021, October–December 2021, January–March 2022, April–June 2022, and July–September 2022) as a time-varying covariate

Supplementary Table 3: Age-stratified results. Crude and adjusted incidence rate ratios of 17 different outcomes between vaccinated and unvaccinated subjects by age-groups(12-15, 16-17, 18-19 years), with associated 95% confidence intervals, based on Poisson regression of adolescents in Norway unvaccinated against SARS-CoV-2 at the beginning of follow-up. Subjects were followed from the beginning of the wave of vaccination^1^ of their age group until the outcome in question, non-mRNA SARS-CoV-2 vaccination, third-dose SARS-CoV-2 vaccination, emigration, death, or end of study on September 30, 2022, whichever occurred first. To ensure data privacy, numbers between 1 and 4 have been suppressed and are denoted by “< 5”. As a result, some of the totals have been suppressed as well to avoid revealing small numbers that have been suppressed.

| **Outcome** | **Risk window** | **Vaccination status** | **Number of subjects** | **Number of events** | **Incidence rate^2^** | **IRR** | **95% CI** | **P value** | **aIRR^3^** | **95% CI^3^** | **P value^3^** |  |
| --- | --- | --- | --- | --- | --- | --- | --- | --- | --- | --- | --- | --- |
| *In 12–15-year-olds (N = 253,669)* |  |  |  |  |  |  |  |  |  |  |  |  |
| Acute appendicitis | 14 days | *Overall* | 251,681 | < 539 | 200.50 |  |  |  |  |  |  |  |
|  |  | Unvaccinated (ref.) | 250,982 | 123 | 151.17 | 1 |  |  | 1 |  |  |  |
|  |  | Vaccinated with 1^st^ dose, inside risk window | 188,097 | 18 | 249.69 | 1.65 | 1.01–2.71 | 0.047 | 1.91 | 1.12–3.27 | 0.017 |  |
|  |  | Vaccinated with 1^st^ dose, outside risk window | 188,054 | 367 | 225.83 | 1.49 | 1.22–1.83 | 0.000 | 1.40 | 1.14–1.74 | 0.002 |  |
|  |  | Vaccinated with 2^nd^ dose, inside risk window | 28,920 | < 5 | 180.78 | 1.20 | 0.30–4.84 | 0.802 | 1.13 | 0.28–4.61 | 0.860 |  |
|  |  | Vaccinated with 2^nd^ dose, outside risk window | 28,813 | 26 | 171.73 | 1.14 | 0.74–1.73 | 0.555 | 1.06 | 0.68–1.63 | 0.806 |  |
| Anaphylactic reaction | 2 days | *Overall* | 253,579 | 21 | 7.79 |  |  |  |  |  |  |  |
|  |  | Unvaccinated (ref.) | 252,869 | 5 | 6.10 | 1 |  |  | 1 |  |  |  |
|  |  | Vaccinated with 1^st^ dose, inside risk window | 189,544 | < 5 | 96.35 | 15.81 | 1.85–135.31 | 0.012 | 16.19 | 1.29–202.98 | 0.031 |  |
|  |  | Vaccinated with 1^st^ dose, outside risk window | 189,535 | 13 | 7.64 | 1.25 | 0.45–3.52 | 0.668 | 1.16 | 0.40–3.38 | 0.779 |  |
|  |  | Vaccinated with 2^nd^ dose, inside risk window | 29,154 | 0 | 0.00 | ND | ND | ND | ND | ND | ND |  |
|  |  | Vaccinated with 2^nd^ dose, outside risk window | 29,124 | < 5 | 12.32 | 2.02 | 0.39–10.42 | 0.401 | 1.35 | 0.25–7.30 | 0.731 |  |
| Arrhythmia | 28 days | *Overall* | 253,474 | 49 | 18.18 |  |  |  |  |  |  |  |
|  |  | Unvaccinated (ref.) | 252,765 | 14 | 17.08 | 1 |  |  | 1 |  |  |  |
|  |  | Vaccinated with 1^st^ dose, inside risk window | 189,459 | < 5 | 6.89 | 0.40 | 0.05–3.07 | 0.380 | 0.31 | 0.04–2.40 | 0.261 |  |
|  |  | Vaccinated with 1^st^ dose, outside risk window | 189,193 | 29 | 18.52 | 1.08 | 0.57–2.05 | 0.803 | 1.26 | 0.62–2.55 | 0.528 |  |
|  |  | Vaccinated with 2^nd^ dose, inside risk window | 29,146 | < 5 | 44.94 | 2.63 | 0.35–20.01 | 0.350 | 3.75 | 0.47–30.02 | 0.213 |  |
|  |  | Vaccinated with 2^nd^ dose, outside risk window | 28,909 | < 5 | 28.25 | 1.65 | 0.54–5.03 | 0.374 | 1.89 | 0.57–6.22 | 0.294 |  |
| Arthropathy | 42 days | *Overall* | 253,666 | 0 | 0.00 |  |  |  |  |  |  |  |
|  |  | Unvaccinated (ref.) | 252,956 | 0 | 0.00 | 1 |  |  | 1 |  |  |  |
|  |  | Vaccinated with 1^st^ dose, inside risk window | 189,617 | 0 | 0.00 | ND | ND | ND | ND | ND | ND |  |
|  |  | Vaccinated with 1^st^ dose, outside risk window | 189,147 | 0 | 0.00 | ND | ND | ND | ND | ND | ND |  |
|  |  | Vaccinated with 2^nd^ dose, inside risk window | 29,176 | 0 | 0.00 | ND | ND | ND | ND | ND | ND |  |
|  |  | Vaccinated with 2^nd^ dose, outside risk window | 28,843 | 0 | 0.00 | ND | ND | ND | ND | ND | ND |  |
| Cerebrovascular events | 28 days | *Overall* | 253,630 | < 15 | 5.19 |  |  |  |  |  |  |  |
|  |  | Unvaccinated (ref.) | 252,920 | < 5 | 4.88 | 1 |  |  | 1 |  |  |  |
|  |  | Vaccinated with 1^st^ dose, inside risk window | 189,585 | 0 | 0.00 | ND | ND | ND | ND | ND | ND |  |
|  |  | Vaccinated with 1^st^ dose, outside risk window | 189,320 | 10 | 6.38 | 1.31 | 0.41–4.17 | 0.649 | 1.28 | 0.35–4.70 | 0.706 |  |
|  |  | Vaccinated with 2^nd^ dose, inside risk window | 29,168 | 0 | 0.00 | ND | ND | ND | ND | ND | ND |  |
|  |  | Vaccinated with 2^nd^ dose, outside risk window | 28,932 | 0 | 0.00 | ND | ND | ND | ND | ND | ND |  |
| Death (all-cause mortality) | 28 days | *Overall* | 253,666 | 32 | 11.86 |  |  |  |  |  |  |  |
|  |  | Unvaccinated (ref.) | 252,956 | 18 | 21.94 | 1 |  |  | 1 |  |  |  |
|  |  | Vaccinated with 1^st^ dose, inside risk window | 189,617 | < 5 | 6.88 | 0.31 | 0.04–2.35 | 0.259 | 0.43 | 0.05–3.46 | 0.426 |  |
|  |  | Vaccinated with 1^st^ dose, outside risk window | 189,352 | 12 | 7.66 | 0.35 | 0.17–0.72 | 0.005 | 0.31 | 0.15–0.66 | 0.002 |  |
|  |  | Vaccinated with 2^nd^ dose, inside risk window | 29,176 | 0 | 0.00 | ND | ND | ND | ND | ND | ND |  |
|  |  | Vaccinated with 2^nd^ dose, outside risk window | 28,940 | < 5 | 7.05 | 0.32 | 0.04–2.41 | 0.269 | 0.22 | 0.03–1.66 | 0.140 |  |
| Encephalomyelitis and meningitis | 28 days | *Overall* | 253,632 | 5 | 1.85 |  |  |  |  |  |  |  |
|  |  | Unvaccinated (ref.) | 252,922 | < 5 | 2.44 | 1 |  |  | 1 |  |  |  |
|  |  | Vaccinated with 1^st^ dose, inside risk window | 189,592 | 0 | 0.00 | ND | ND | ND | ND | ND | ND |  |
|  |  | Vaccinated with 1^st^ dose, outside risk window | 189,327 | < 5 | 1.91 | 0.78 | 0.13–4.70 | 0.791 | 0.71 | 0.12–4.27 | 0.708 |  |
|  |  | Vaccinated with 2^nd^ dose, inside risk window | 29,169 | 0 | 0.00 | ND | ND | ND | ND | ND | ND |  |
|  |  | Vaccinated with 2^nd^ dose, outside risk window | 28,933 | 0 | 0.00 | ND | ND | ND | ND | ND | ND |  |
| Epilepsy and convulsions | 28 days | *Overall* | 251,005 | < 301 | 111.34 |  |  |  |  |  |  |  |
|  |  | Unvaccinated (ref.) | 250,300 | 92 | 113.45 | 1 |  |  | 1 |  |  |  |
|  |  | Vaccinated with 1^st^ dose, inside risk window | 187,667 | 18 | 125.16 | 1.10 | 0.67–1.83 | 0.703 | 1.28 | 0.75–2.18 | 0.368 |  |
|  |  | Vaccinated with 1^st^ dose, outside risk window | 187,388 | 170 | 109.62 | 0.97 | 0.75–1.25 | 0.790 | 0.91 | 0.70–1.18 | 0.467 |  |
|  |  | Vaccinated with 2^nd^ dose, inside risk window | 28,818 | < 5 | 45.45 | 0.40 | 0.06–2.87 | 0.363 | 0.33 | 0.05–2.37 | 0.269 |  |
|  |  | Vaccinated with 2^nd^ dose, outside risk window | 28,583 | 16 | 114.43 | 1.01 | 0.59–1.72 | 0.975 | 0.92 | 0.53–1.58 | 0.751 |  |
| Facial nerve palsy | 28 days | *Overall* | 253,469 | 37 | 13.73 |  |  |  |  |  |  |  |
|  |  | Unvaccinated (ref.) | 252,759 | 18 | 21.96 | 1 |  |  | 1 |  |  |  |
|  |  | Vaccinated with 1^st^ dose, inside risk window | 189,480 | < 5 | 13.77 | 0.63 | 0.15–2.70 | 0.532 | 0.76 | 0.16–3.57 | 0.731 |  |
|  |  | Vaccinated with 1^st^ dose, outside risk window | 189,213 | 14 | 8.94 | 0.41 | 0.20–0.82 | 0.012 | 0.38 | 0.18–0.78 | 0.008 |  |
|  |  | Vaccinated with 2^nd^ dose, inside risk window | 29,144 | 0 | 0.00 | ND | ND | ND | ND | ND | ND |  |
|  |  | Vaccinated with 2^nd^ dose, outside risk window | 28,908 | < 5 | 21.19 | 0.96 | 0.28–3.28 | 0.954 | 0.73 | 0.21–2.59 | 0.626 |  |
| Guillain-Barré syndrome | 42 days | *Overall* | 253,663 | < 5 | 1.11 |  |  |  |  |  |  |  |
|  |  | Unvaccinated (ref.) | 252,953 | 0 | 0.00 | 1 |  |  | 1 |  |  |  |
|  |  | Vaccinated with 1^st^ dose, inside risk window | 189,614 | 0 | 0.00 | ND | ND | ND | ND | ND | ND |  |
|  |  | Vaccinated with 1^st^ dose, outside risk window | 189,144 | < 5 | 1.34 | ND | ND | ND | ND | ND | ND |  |
|  |  | Vaccinated with 2^nd^ dose, inside risk window | 29,175 | 0 | 0.00 | ND | ND | ND | ND | ND | ND |  |
|  |  | Vaccinated with 2^nd^ dose, outside risk window | 28,842 | < 5 | 7.65 | ND | ND | ND | ND | ND | ND |  |
| IgA vasculitis | 42 days | *Overall* | 253,622 | < 10 | 2.22 |  |  |  |  |  |  |  |
|  |  | Unvaccinated (ref.) | 252,912 | < 5 | 1.22 | 1 |  |  | 1 |  |  |  |
|  |  | Vaccinated with 1^st^ dose, inside risk window | 189,586 | 0 | 0.00 | ND | ND | ND | ND | ND | ND |  |
|  |  | Vaccinated with 1^st^ dose, outside risk window | 189,116 | 5 | 3.35 | 2.74 | 0.32–23.49 | 0.357 | ND | ND | ND |  |
|  |  | Vaccinated with 2^nd^ dose, inside risk window | 29,173 | 0 | 0.00 | ND | ND | ND | ND | ND | ND |  |
|  |  | Vaccinated with 2^nd^ dose, outside risk window | 28,840 | 0 | 0.00 | ND | ND | ND | ND | ND | ND |  |
| Herpes zoster | 28 days | *Overall* | 253,556 | < 44 | 14.84 |  |  |  |  |  |  |  |
|  |  | Unvaccinated (ref.) | 252,846 | 10 | 12.19 | 1 |  |  | 1 |  |  |  |
|  |  | Vaccinated with 1^st^ dose, inside risk window | 189,529 | < 5 | 6.88 | 0.56 | 0.07–4.41 | 0.586 | 1.23 | 0.13–11.20 | 0.857 |  |
|  |  | Vaccinated with 1^st^ dose, outside risk window | 189,263 | 24 | 15.32 | 1.26 | 0.60–2.63 | 0.544 | 1.01 | 0.47–2.16 | 0.976 |  |
|  |  | Vaccinated with 2^nd^ dose, inside risk window | 29,146 | 0 | 0.00 | ND | ND | ND | ND | ND | ND |  |
|  |  | Vaccinated with 2^nd^ dose, outside risk window | 28,911 | 5 | 35.32 | 2.90 | 0.99–8.48 | 0.052 | 1.57 | 0.52–4.75 | 0.427 |  |
| Idiopathic thrombocytopenic | 28 days | *Overall* | 253,601 | 13 | 4.82 |  |  |  |  |  |  |  |
| purpura |  | Unvaccinated (ref.) | 252,891 | < 5 | 3.66 | 1 |  |  | 1 |  |  |  |
|  |  | Vaccinated with 1^st^ dose, inside risk window | 189,572 | < 5 | 13.77 | 3.76 | 0.63–22.51 | 0.147 | 3.06 | 0.42–22.09 | 0.267 |  |
|  |  | Vaccinated with 1^st^ dose, outside risk window | 189,305 | 8 | 5.11 | 1.39 | 0.37–5.26 | 0.623 | 1.96 | 0.40–9.74 | 0.408 |  |
|  |  | Vaccinated with 2^nd^ dose, inside risk window | 29,168 | 0 | 0.00 | ND | ND | ND | ND | ND | ND |  |
|  |  | Vaccinated with 2^nd^ dose, outside risk window | 28,933 | 0 | 0.00 | ND | ND | ND | ND | ND | ND |  |
| Lymphadenopathy | 14 days | *Overall* | 252,508 | < 293 | 108.07 |  |  |  |  |  |  |  |
|  |  | Unvaccinated (ref.) | 251,803 | 74 | 90.65 | 1 |  |  | 1 |  |  |  |
|  |  | Vaccinated with 1^st^ dose, inside risk window | 188,737 | 6 | 82.95 | 0.92 | 0.40–2.10 | 0.834 | 1.12 | 0.47–2.69 | 0.798 |  |
|  |  | Vaccinated with 1^st^ dose, outside risk window | 188,706 | 185 | 113.37 | 1.25 | 0.96–1.64 | 0.104 | 1.15 | 0.87–1.52 | 0.338 |  |
|  |  | Vaccinated with 2^nd^ dose, inside risk window | 28,993 | < 5 | 180.34 | 1.99 | 0.49–8.10 | 0.337 | 1.65 | 0.40–6.78 | 0.487 |  |
|  |  | Vaccinated with 2^nd^ dose, outside risk window | 28,885 | 23 | 151.57 | 1.67 | 1.05–2.67 | 0.031 | 1.53 | 0.94–2.48 | 0.087 |  |
| Multisystem inflammatory | 42 days | *Overall* | 253,650 | 12 | 4.45 |  |  |  |  |  |  |  |
| syndrome in children |  | Unvaccinated (ref.) | 252,940 | 5 | 6.09 | 1 |  |  | 1 |  |  |  |
|  |  | Vaccinated with 1^st^ dose, inside risk window | 189,607 | 0 | 0.00 | ND | ND | ND | ND | ND | ND |  |
|  |  | Vaccinated with 1^st^ dose, outside risk window | 189,137 | 7 | 4.68 | 0.77 | 0.24–2.42 | 0.652 | 1.16 | 0.29–4.55 | 0.834 |  |
|  |  | Vaccinated with 2^nd^ dose, inside risk window | 29,176 | 0 | 0.00 | ND | ND | ND | ND | ND | ND |  |
|  |  | Vaccinated with 2^nd^ dose, outside risk window | 28,843 | 0 | 0.00 | ND | ND | ND | ND | ND | ND |  |
| Myocarditis and pericarditis | 28 days | *Overall* | 253,659 | 10 | 3.71 |  |  |  |  |  |  |  |
|  |  | Unvaccinated (ref.) | 252,949 | < 5 | 3.66 | 1 |  |  | 1 |  |  |  |
|  |  | Vaccinated with 1^st^ dose, inside risk window | 189,611 | 0 | 0.00 | ND | ND | ND | ND | ND | ND |  |
|  |  | Vaccinated with 1^st^ dose, outside risk window | 189,346 | 5 | 3.19 | 0.87 | 0.21–3.65 | 0.852 | 1.30 | 0.25–6.75 | 0.757 |  |
|  |  | Vaccinated with 2^nd^ dose, inside risk window | 29,174 | < 5 | 44.89 | 12.27 | 1.28–117.98 | 0.030 | 37.07 | 2.79–492.94 | 0.006 |  |
|  |  | Vaccinated with 2^nd^ dose, outside risk window | 28,937 | < 5 | 7.06 | 1.93 | 0.20–18.54 | 0.569 | 4.12 | 0.31–54.93 | 0.285 | |
| Venous thromboembolic events | 28 days | *Overall* | 253,650 | 16 | 5.93 |  |  |  |  |  |  | |
|  |  | Unvaccinated (ref.) | 252,940 | < 5 | 2.44 | 1 |  |  | 1 |  |  | |
|  |  | Vaccinated with 1^st^ dose, inside risk window | 189,604 | 0 | 0.00 | ND | ND | ND | ND | ND | ND | |
|  |  | Vaccinated with 1^st^ dose, outside risk window | 189,339 | 13 | 8.29 | 3.41 | 0.77–15.10 | 0.107 | 6.03 | 0.78–46.37 | 0.084 | |
|  |  | Vaccinated with 2^nd^ dose, inside risk window | 29,169 | 0 | 0.00 | ND | ND | ND | ND | ND | ND | |
|  |  | Vaccinated with 2^nd^ dose, outside risk window | 28,933 | < 5 | 7.06 | 2.90 | 0.26–31.96 | 0.385 | 8.94 | 0.52–154.10 | 0.131 | |
| *In 16-17-year-olds (N = 121,179)* |  |  |  |  |  |  |  |  |  |  |  | |
| Acute appendicitis | 14 days | *Overall* | 120,117 | 300 | 227.97 |  |  |  |  |  |  | |
|  |  | Unvaccinated (ref.) | 120,049 | 26 | 122.19 | 1 |  |  | 1 |  |  | |
|  |  | Vaccinated with 1^st^ dose, inside risk window | 106,881 | 7 | 170.88 | 1.40 | 0.61–3.22 | 0.431 | 1.07 | 0.45–2.56 | 0.878 | |
|  |  | Vaccinated with 1^st^ dose, outside risk window | 106,863 | 81 | 261.83 | 2.14 | 1.38–3.33 | 0.001 | 2.38 | 1.50–3.77 | 0.000 | |
|  |  | Vaccinated with 2^nd^ dose, inside risk window | 91,662 | 10 | 284.68 | 2.33 | 1.12–4.83 | 0.023 | 2.81 | 1.30–6.09 | 0.009 | |
|  |  | Vaccinated with 2^nd^ dose, outside risk window | 91,618 | 176 | 245.22 | 2.01 | 1.33–3.03 | 0.001 | 2.19 | 1.38–3.48 | 0.001 | |
| Anaphylactic reaction | 2 days | *Overall* | 121,129 | < 17 | 10.54 |  |  |  |  |  |  | |
|  |  | Unvaccinated (ref.) | 121,060 | < 5 | 9.32 | 1 |  |  | 1 |  |  | |
|  |  | Vaccinated with 1^st^ dose, inside risk window | 107,798 | 0 | 0.00 | ND | ND | ND | ND | ND | ND | |
|  |  | Vaccinated with 1^st^ dose, outside risk window | 107,796 | 5 | 14.37 | 1.54 | 0.30–7.96 | 0.604 | 1.79 | 0.34–9.43 | 0.495 | |
|  |  | Vaccinated with 2^nd^ dose, inside risk window | 92,475 | 0 | 0.00 | ND | ND | ND | ND | ND | ND | |
|  |  | Vaccinated with 2^nd^ dose, outside risk window | 92,474 | 7 | 9.27 | 0.99 | 0.21–4.79 | 0.995 | 0.82 | 0.15–4.55 | 0.823 | |
| Arrhythmia | 28 days | *Overall* | 121,024 | < 36 | 24.11 |  |  |  |  |  |  | |
|  |  | Unvaccinated (ref.) | 120,956 | 6 | 27.98 | 1 |  |  | 1 |  |  | |
|  |  | Vaccinated with 1^st^ dose, inside risk window | 107,700 | < 5 | 12.13 | 0.43 | 0.05–3.60 | 0.438 | 0.33 | 0.04–2.98 | 0.324 | |
|  |  | Vaccinated with 1^st^ dose, outside risk window | 106,724 | 6 | 22.15 | 0.79 | 0.26–2.45 | 0.685 | 1.23 | 0.32–4.66 | 0.763 | |
|  |  | Vaccinated with 2^nd^ dose, inside risk window | 92,396 | 0 | 0.00 | ND | ND | ND | ND | ND | ND | |
|  |  | Vaccinated with 2^nd^ dose, outside risk window | 92,318 | 19 | 27.59 | 0.99 | 0.39–2.47 | 0.976 | 1.63 | 0.47–5.64 | 0.437 | |
| Arthropathy | 42 days | *Overall* | 121,179 | 0 | 0.00 |  |  |  |  |  |  | |
|  |  | Unvaccinated (ref.) | 121,110 | 0 | 0.00 | 1 |  |  | 1 |  |  | |
|  |  | Vaccinated with 1^st^ dose, inside risk window | 107,846 | 0 | 0.00 | ND | ND | ND | ND | ND | ND | |
|  |  | Vaccinated with 1^st^ dose, outside risk window | 105,676 | 0 | 0.00 | ND | ND | ND | ND | ND | ND | |
|  |  | Vaccinated with 2^nd^ dose, inside risk window | 92,518 | 0 | 0.00 | ND | ND | ND | ND | ND | ND | |
|  |  | Vaccinated with 2^nd^ dose, outside risk window | 92,393 | 0 | 0.00 | ND | ND | ND | ND | ND | ND | |
| Cerebrovascular events | 28 days | *Overall* | 121,144 | 11 | 8.28 |  |  |  |  |  |  | |
|  |  | Unvaccinated (ref.) | 121,075 | < 5 | 9.32 | 1 |  |  | 1 |  |  | |
|  |  | Vaccinated with 1^st^ dose, inside risk window | 107,818 | < 5 | 36.34 | 3.90 | 0.65–23.33 | 0.136 | 5.52 | 0.71–42.97 | 0.102 | |
|  |  | Vaccinated with 1^st^ dose, outside risk window | 106,839 | < 5 | 7.37 | 0.79 | 0.11–5.61 | 0.815 | 0.61 | 0.08–4.51 | 0.630 | |
|  |  | Vaccinated with 2^nd^ dose, inside risk window | 92,488 | 0 | 0.00 | ND | ND | ND | ND | ND | ND | |
|  |  | Vaccinated with 2^nd^ dose, outside risk window | 92,410 | < 5 | 5.80 | 0.62 | 0.11–3.40 | 0.584 | 0.56 | 0.09–3.56 | 0.542 | |
| Death (all-cause mortality) | 28 days | *Overall* | 121,179 | < 31 | 21.82 |  |  |  |  |  |  | |
|  |  | Unvaccinated (ref.) | 121,110 | 5 | 23.29 | 1 |  |  | 1 |  |  | |
|  |  | Vaccinated with 1^st^ dose, inside risk window | 107,846 | 0 | 0.00 | ND | ND | ND | ND | ND | ND | |
|  |  | Vaccinated with 1^st^ dose, outside risk window | 106,869 | 8 | 29.48 | 1.27 | 0.41–3.87 | 0.679 | 1.20 | 0.33–4.31 | 0.778 | |
|  |  | Vaccinated with 2^nd^ dose, inside risk window | 92,518 | < 5 | 42.31 | 1.82 | 0.43–7.60 | 0.414 | 1.42 | 0.28–7.17 | 0.668 | |
|  |  | Vaccinated with 2^nd^ dose, outside risk window | 92,440 | 13 | 18.85 | 0.81 | 0.29–2.27 | 0.687 | 1.16 | 0.32–4.23 | 0.818 | |
| Encephalomyelitis and meningitis | 28 days | *Overall* | 121,166 | < 5 | 1.50 |  |  |  |  |  |  | |
|  |  | Unvaccinated (ref.) | 121,097 | < 5 | 4.66 | 1 |  |  | 1 |  |  | |
|  |  | Vaccinated with 1^st^ dose, inside risk window | 107,834 | 0 | 0.00 | ND | ND | ND | ND | ND | ND | |
|  |  | Vaccinated with 1^st^ dose, outside risk window | 106,858 | < 5 | 3.69 | 0.79 | 0.05–12.64 | 0.868 | 0.60 | 0.04–10.25 | 0.726 | |
|  |  | Vaccinated with 2^nd^ dose, inside risk window | 92,508 | 0 | 0.00 | ND | ND | ND | ND | ND | ND | |
|  |  | Vaccinated with 2^nd^ dose, outside risk window | 92,431 | 0 | 0.00 | ND | ND | ND | ND | ND | ND | |
| Epilepsy and convulsions | 28 days | *Overall* | 119,958 | 165 | 125.48 |  |  |  |  |  |  | |
|  |  | Unvaccinated (ref.) | 119,891 | 26 | 122.46 | 1 |  |  | 1 |  |  | |
|  |  | Vaccinated with 1^st^ dose, inside risk window | 106,752 | 6 | 73.40 | 0.60 | 0.25–1.46 | 0.258 | 0.62 | 0.24–1.61 | 0.326 | |
|  |  | Vaccinated with 1^st^ dose, outside risk window | 105,783 | 34 | 126.69 | 1.03 | 0.62–1.72 | 0.896 | 0.92 | 0.53–1.60 | 0.766 | |
|  |  | Vaccinated with 2^nd^ dose, inside risk window | 91,573 | 15 | 213.77 | 1.75 | 0.92–3.30 | 0.086 | 1.36 | 0.68–2.73 | 0.383 | |
|  |  | Vaccinated with 2^nd^ dose, outside risk window | 91,482 | 84 | 123.11 | 1.01 | 0.65–1.56 | 0.981 | 1.00 | 0.60–1.67 | 0.997 | |
| Facial nerve palsy | 28 days | *Overall* | 121,078 | 28 | 21.09 |  |  |  |  |  |  | |
|  |  | Unvaccinated (ref.) | 121,009 | 5 | 23.31 | 1 |  |  | 1 |  |  | |
|  |  | Vaccinated with 1^st^ dose, inside risk window | 107,751 | < 5 | 12.12 | 0.52 | 0.06–4.45 | 0.550 | 0.26 | 0.03–2.29 | 0.225 | |
|  |  | Vaccinated with 1^st^ dose, outside risk window | 106,775 | 6 | 22.14 | 0.95 | 0.29–3.11 | 0.932 | 1.86 | 0.39–8.79 | 0.432 | |
|  |  | Vaccinated with 2^nd^ dose, inside risk window | 92,437 | < 5 | 28.23 | 1.21 | 0.23–6.24 | 0.819 | 2.07 | 0.28–15.52 | 0.480 | |
|  |  | Vaccinated with 2^nd^ dose, outside risk window | 92,357 | 14 | 20.32 | 0.87 | 0.31–2.42 | 0.792 | 3.15 | 0.62–16.12 | 0.168 | |
| Guillain-Barré syndrome | 42 days | *Overall* | 121,172 | < 5 | 1.50 |  |  |  |  |  |  | |
|  |  | Unvaccinated (ref.) | 121,103 | 0 | 0.00 | 1 |  |  | 1 |  |  | |
|  |  | Vaccinated with 1^st^ dose, inside risk window | 107,839 | 0 | 0.00 | ND | ND | ND | ND | ND | ND | |
|  |  | Vaccinated with 1^st^ dose, outside risk window | 105,669 | 0 | 0.00 | ND | ND | ND | ND | ND | ND | |
|  |  | Vaccinated with 2^nd^ dose, inside risk window | 92,512 | 0 | 0.00 | ND | ND | ND | ND | ND | ND | |
|  |  | Vaccinated with 2^nd^ dose, outside risk window | 92,387 | < 5 | 3.06 | ND | ND | ND | ND | ND | ND | |
| IgA vasculitis | 42 days | *Overall* | 121,166 | 6 | 4.51 |  |  |  |  |  |  | |
|  |  | Unvaccinated (ref.) | 121,097 | < 5 | 4.66 | 1 |  |  | 1 |  |  | |
|  |  | Vaccinated with 1^st^ dose, inside risk window | 107,832 | 0 | 0.00 | ND | ND | ND | ND | ND | ND | |
|  |  | Vaccinated with 1^st^ dose, outside risk window | 105,662 | < 5 | 8.67 | 1.86 | 0.17–20.53 | 0.612 | 1.12 | 0.10–12.83 | 0.926 | |
|  |  | Vaccinated with 2^nd^ dose, inside risk window | 92,504 | 0 | 0.00 | ND | ND | ND | ND | ND | ND | |
|  |  | Vaccinated with 2^nd^ dose, outside risk window | 92,379 | < 5 | 4.59 | 0.98 | 0.10–9.46 | 0.989 | 0.78 | 0.06–9.44 | 0.843 | |
| Herpes zoster | 28 days | *Overall* | 121,117 | 21 | 15.81 |  |  |  |  |  |  | |
|  |  | Unvaccinated (ref.) | 121,048 | < 5 | 13.98 | 1 |  |  | 1 |  |  | |
|  |  | Vaccinated with 1^st^ dose, inside risk window | 107,792 | < 5 | 12.12 | 0.87 | 0.09–8.34 | 0.901 | 2.72 | 0.14–52.29 | 0.507 | |
|  |  | Vaccinated with 1^st^ dose, outside risk window | 106,815 | < 5 | 7.38 | 0.53 | 0.09–3.16 | 0.484 | 0.57 | 0.09–3.47 | 0.542 | |
|  |  | Vaccinated with 2^nd^ dose, inside risk window | 92,476 | 0 | 0.00 | ND | ND | ND | ND | ND | ND | |
|  |  | Vaccinated with 2^nd^ dose, outside risk window | 92,398 | 15 | 21.76 | 1.56 | 0.45–5.38 | 0.484 | 0.68 | 0.19–2.41 | 0.545 | |
| Idiopathic thrombocytopenic | 28 days | *Overall* | 121,150 | < 5 | 2.26 |  |  |  |  |  |  | |
| purpura |  | Unvaccinated (ref.) | 121,081 | 0 | 0.00 | 1 |  |  | 1 |  |  | |
|  |  | Vaccinated with 1^st^ dose, inside risk window | 107,824 | 0 | 0.00 | ND | ND | ND | ND | ND | ND | |
|  |  | Vaccinated with 1^st^ dose, outside risk window | 106,848 | 0 | 0.00 | ND | ND | ND | ND | ND | ND | |
|  |  | Vaccinated with 2^nd^ dose, inside risk window | 92,501 | < 5 | 14.11 | ND | ND | ND | ND | ND | ND | |
|  |  | Vaccinated with 2^nd^ dose, outside risk window | 92,422 | < 5 | 2.90 | ND | ND | ND | ND | ND | ND | |
| Lymphadenopathy | 14 days | *Overall* | 120,635 | < 181 | 135.37 |  |  |  |  |  |  | |
|  |  | Unvaccinated (ref.) | 120,566 | 26 | 121.68 | 1 |  |  | 1 |  |  | |
|  |  | Vaccinated with 1^st^ dose, inside risk window | 107,344 | < 5 | 72.92 | 0.60 | 0.18–1.98 | 0.401 | 0.70 | 0.20–2.50 | 0.587 | |
|  |  | Vaccinated with 1^st^ dose, outside risk window | 107,330 | 49 | 157.61 | 1.30 | 0.81–2.08 | 0.286 | 1.16 | 0.71–1.91 | 0.548 | |
|  |  | Vaccinated with 2^nd^ dose, inside risk window | 92,070 | 10 | 283.42 | 2.33 | 1.12–4.83 | 0.023 | 1.90 | 0.87–4.13 | 0.105 | |
|  |  | Vaccinated with 2^nd^ dose, outside risk window | 92,026 | 91 | 126.16 | 1.04 | 0.67–1.60 | 0.871 | 0.95 | 0.58–1.54 | 0.825 | |
| Multisystem inflammatory | 42 days | *Overall* | 121,176 | 8 | 6.02 |  |  |  |  |  |  | |
| syndrome in children |  | Unvaccinated (ref.) | 121,107 | < 5 | 4.66 | 1 |  |  | 1 |  |  | |
|  |  | Vaccinated with 1^st^ dose, inside risk window | 107,843 | < 5 | 24.33 | 5.22 | 0.54–50.23 | 0.152 | 5.99 | 0.48–75.25 | 0.166 | |
|  |  | Vaccinated with 1^st^ dose, outside risk window | 105,670 | < 5 | 4.34 | 0.93 | 0.06–14.89 | 0.960 | 0.74 | 0.05–12.22 | 0.836 | |
|  |  | Vaccinated with 2^nd^ dose, inside risk window | 92,516 | 0 | 0.00 | ND | ND | ND | ND | ND | ND | |
|  |  | Vaccinated with 2^nd^ dose, outside risk window | 92,391 | < 5 | 4.58 | 0.98 | 0.10–9.47 | 0.989 | 0.95 | 0.08–12.00 | 0.968 | |
| Myocarditis and pericarditis | 28 days | *Overall* | 121,169 | 18 | 13.54 |  |  |  |  |  |  | |
|  |  | Unvaccinated (ref.) | 121,100 | < 5 | 9.32 | 1 |  |  | 1 |  |  | |
|  |  | Vaccinated with 1^st^ dose, inside risk window | 107,839 | < 5 | 12.11 | 1.30 | 0.12–14.33 | 0.831 | 1.54 | 0.11–20.77 | 0.746 | |
|  |  | Vaccinated with 1^st^ dose, outside risk window | 106,861 | 7 | 25.80 | 2.77 | 0.58–13.33 | 0.204 | 2.15 | 0.41–11.17 | 0.362 | |
|  |  | Vaccinated with 2^nd^ dose, inside risk window | 92,509 | < 5 | 14.11 | 1.51 | 0.14–16.69 | 0.735 | 1.00 | 0.08–12.12 | 1.000 | |
|  |  | Vaccinated with 2^nd^ dose, outside risk window | 92,430 | 7 | 10.15 | 1.09 | 0.23–5.24 | 0.915 | 1.43 | 0.25–8.33 | 0.688 | |
| Venous thromboembolic events | 28 days | *Overall* | 121,147 | 27 | 20.32 |  |  |  |  |  |  | |
|  |  | Unvaccinated (ref.) | 121,078 | < 5 | 13.98 | 1 |  |  | 1 |  |  | |
|  |  | Vaccinated with 1^st^ dose, inside risk window | 107,816 | < 5 | 24.23 | 1.73 | 0.29–10.37 | 0.547 | 1.39 | 0.19–10.44 | 0.748 | |
|  |  | Vaccinated with 1^st^ dose, outside risk window | 106,837 | 5 | 18.43 | 1.32 | 0.32–5.52 | 0.705 | 1.51 | 0.31–7.41 | 0.609 | |
|  |  | Vaccinated with 2^nd^ dose, inside risk window | 92,488 | < 5 | 14.11 | 1.01 | 0.10–9.70 | 0.994 | 1.31 | 0.11–15.54 | 0.829 | |
|  |  | Vaccinated with 2^nd^ dose, outside risk window | 92,409 | 16 | 23.21 | 1.66 | 0.48–5.70 | 0.421 | 1.36 | 0.31–5.94 | 0.679 | |
| *In 18-19-year-olds (N = 121,584)* |  |  |  |  |  |  |  |  |  |  |  | |
| Acute appendicitis | 14 days | *Overall* | 120,562 | 349 | 250.73 |  |  |  |  |  |  | |
|  |  | Unvaccinated (ref.) | 120,558 | 118 | 267.23 | 1 |  |  | 1 |  |  | |
|  |  | Vaccinated with 1^st^ dose, inside risk window | 110,711 | 11 | 259.25 | 0.97 | 0.52–1.80 | 0.923 | 0.94 | 0.49–1.81 | 0.859 | |
|  |  | Vaccinated with 1^st^ dose, outside risk window | 110,671 | 47 | 273.35 | 1.02 | 0.73–1.43 | 0.895 | 1.09 | 0.72–1.64 | 0.696 | |
|  |  | Vaccinated with 2^nd^ dose, inside risk window | 104,941 | 9 | 223.78 | 0.84 | 0.43–1.65 | 0.608 | 0.83 | 0.40–1.71 | 0.607 | |
|  |  | Vaccinated with 2^nd^ dose, outside risk window | 104,910 | 164 | 235.70 | 0.88 | 0.70–1.12 | 0.298 | 1.02 | 0.71–1.46 | 0.929 | |
| Anaphylactic reaction | 2 days | *Overall* | 121,518 | < 25 | 14.94 |  |  |  |  |  |  | |
|  |  | Unvaccinated (ref.) | 121,514 | 5 | 11.23 | 1 |  |  | 1 |  |  | |
|  |  | Vaccinated with 1^st^ dose, inside risk window | 111,693 | 0 | 0.00 | ND | ND | ND | ND | ND | ND | |
|  |  | Vaccinated with 1^st^ dose, outside risk window | 111,690 | 8 | 38.05 | 3.39 | 1.11–10.36 | 0.032 | 8.49 | 1.91–37.66 | 0.005 | |
|  |  | Vaccinated with 2^nd^ dose, inside risk window | 105,912 | < 5 | 172.43 | 15.36 | 1.79–131.46 | 0.013 | 38.78 | 3.46–434.39 | 0.003 | |
|  |  | Vaccinated with 2^nd^ dose, outside risk window | 105,909 | 7 | 9.49 | 0.84 | 0.27–2.66 | 0.774 | 2.10 | 0.37–12.07 | 0.406 | |
| Arrhythmia | 28 days | *Overall* | 121,337 | < 57 | 38.49 |  |  |  |  |  |  | |
|  |  | Unvaccinated (ref.) | 121,333 | 19 | 42.73 | 1 |  |  | 1 |  |  | |
|  |  | Vaccinated with 1^st^ dose, inside risk window | 111,517 | < 5 | 23.55 | 0.55 | 0.13–2.37 | 0.422 | 0.52 | 0.11–2.50 | 0.418 | |
|  |  | Vaccinated with 1^st^ dose, outside risk window | 101,548 | 8 | 61.02 | 1.43 | 0.63–3.26 | 0.398 | 1.06 | 0.42–2.66 | 0.907 | |
|  |  | Vaccinated with 2^nd^ dose, inside risk window | 105,749 | 0 | 0.00 | ND | ND | ND | ND | ND | ND | |
|  |  | Vaccinated with 2^nd^ dose, outside risk window | 105,684 | 25 | 37.80 | 0.88 | 0.49–1.61 | 0.687 | 0.58 | 0.27–1.26 | 0.169 | |
| Arthropathy | 42 days | *Overall* | 121,569 | 0 | 0.00 |  |  |  |  |  |  | |
|  |  | Unvaccinated (ref.) | 121,565 | 0 | 0.00 | 1 |  |  | 1 |  |  | |
|  |  | Vaccinated with 1^st^ dose, inside risk window | 111,743 | 0 | 0.00 | ND | ND | ND | ND | ND | ND | |
|  |  | Vaccinated with 1^st^ dose, outside risk window | 67,275 | 0 | 0.00 | ND | ND | ND | ND | ND | ND | |
|  |  | Vaccinated with 2^nd^ dose, inside risk window | 105,968 | 0 | 0.00 | ND | ND | ND | ND | ND | ND | |
|  |  | Vaccinated with 2^nd^ dose, outside risk window | 105,857 | 0 | 0.00 | ND | ND | ND | ND | ND | ND | |
| Cerebrovascular events | 28 days | *Overall* | 121,533 | 6 | 4.27 |  |  |  |  |  |  | |
|  |  | Unvaccinated (ref.) | 121,529 | < 5 | 8.98 | 1 |  |  | 1 |  |  | |
|  |  | Vaccinated with 1^st^ dose, inside risk window | 111,706 | 0 | 0.00 | ND | ND | ND | ND | ND | ND | |
|  |  | Vaccinated with 1^st^ dose, outside risk window | 101,722 | 0 | 0.00 | ND | ND | ND | ND | ND | ND | |
|  |  | Vaccinated with 2^nd^ dose, inside risk window | 105,931 | 0 | 0.00 | ND | ND | ND | ND | ND | ND | |
|  |  | Vaccinated with 2^nd^ dose, outside risk window | 105,866 | < 5 | 3.02 | 0.34 | 0.06–1.84 | 0.208 | 1.06 | 0.06–19.60 | 0.971 | |
| Death (all-cause mortality) | 28 days | *Overall* | 121,569 | 48 | 34.14 |  |  |  |  |  |  | |
|  |  | Unvaccinated (ref.) | 121,565 | 18 | 40.41 | 1 |  |  | 1 |  |  | |
|  |  | Vaccinated with 1^st^ dose, inside risk window | 111,743 | 6 | 70.52 | 1.75 | 0.69–4.40 | 0.237 | 1.53 | 0.52–4.50 | 0.439 | |
|  |  | Vaccinated with 1^st^ dose, outside risk window | 101,754 | < 5 | 22.84 | 0.57 | 0.17–1.92 | 0.360 | 0.70 | 0.18–2.73 | 0.610 | |
|  |  | Vaccinated with 2^nd^ dose, inside risk window | 105,968 | < 5 | 24.63 | 0.61 | 0.14–2.63 | 0.507 | 0.59 | 0.12–2.92 | 0.521 | |
|  |  | Vaccinated with 2^nd^ dose, outside risk window | 105,903 | 19 | 28.67 | 0.71 | 0.37–1.35 | 0.297 | 1.30 | 0.46–3.66 | 0.623 | |
| Encephalomyelitis and meningitis | 28 days | *Overall* | 121,551 | 7 | 4.98 |  |  |  |  |  |  | |
|  |  | Unvaccinated (ref.) | 121,547 | < 5 | 2.25 | 1 |  |  | 1 |  |  | |
|  |  | Vaccinated with 1^st^ dose, inside risk window | 111,727 | 0 | 0.00 | ND | ND | ND | ND | ND | ND | |
|  |  | Vaccinated with 1^st^ dose, outside risk window | 101,739 | < 5 | 7.61 | 3.39 | 0.21–54.23 | 0.388 | 1.43 | 0.09–22.99 | 0.799 | |
|  |  | Vaccinated with 2^nd^ dose, inside risk window | 105,952 | < 5 | 12.32 | 5.48 | 0.34–87.69 | 0.229 | 4.67 | 0.22–100.73 | 0.325 | |
|  |  | Vaccinated with 2^nd^ dose, outside risk window | 105,886 | < 5 | 6.04 | 2.69 | 0.30–24.06 | 0.376 | 0.80 | 0.09–7.48 | 0.846 | |
| Epilepsy and convulsions | 28 days | *Overall* | 120,364 | 155 | 111.40 |  |  |  |  |  |  | |
|  |  | Unvaccinated (ref.) | 120,360 | 47 | 106.53 | 1 |  |  | 1 |  |  | |
|  |  | Vaccinated with 1^st^ dose, inside risk window | 110,582 | 5 | 59.38 | 0.56 | 0.22–1.40 | 0.214 | 0.73 | 0.27–1.98 | 0.538 | |
|  |  | Vaccinated with 1^st^ dose, outside risk window | 100,711 | 21 | 161.49 | 1.52 | 0.91–2.54 | 0.113 | 1.85 | 0.97–3.54 | 0.064 | |
|  |  | Vaccinated with 2^nd^ dose, inside risk window | 104,852 | 14 | 174.23 | 1.64 | 0.90–2.97 | 0.106 | 2.20 | 1.05–4.61 | 0.036 | |
|  |  | Vaccinated with 2^nd^ dose, outside risk window | 104,776 | 68 | 103.72 | 0.97 | 0.67–1.41 | 0.888 | 1.08 | 0.60–1.93 | 0.806 | |
| Facial nerve palsy | 28 days | *Overall* | 121,480 | < 30 | 18.51 |  |  |  |  |  |  | |
|  |  | Unvaccinated (ref.) | 121,476 | 11 | 24.71 | 1 |  |  | 1 |  |  | |
|  |  | Vaccinated with 1^st^ dose, inside risk window | 111,654 | < 5 | 11.76 | 0.48 | 0.06–3.69 | 0.478 | 0.70 | 0.07–6.96 | 0.762 | |
|  |  | Vaccinated with 1^st^ dose, outside risk window | 101,671 | 0 | 0.00 | ND | ND | ND | ND | ND | ND | |
|  |  | Vaccinated with 2^nd^ dose, inside risk window | 105,888 | 0 | 0.00 | ND | ND | ND | ND | ND | ND | |
|  |  | Vaccinated with 2^nd^ dose, outside risk window | 105,823 | 14 | 21.14 | 0.86 | 0.39–1.88 | 0.699 | 0.48 | 0.16–1.40 | 0.180 | |
| Guillain-Barré syndrome | 42 days | *Overall* | 121,566 | < 5 | 0.71 |  |  |  |  |  |  | |
|  |  | Unvaccinated (ref.) | 121,562 | 0 | 0.00 | 1 |  |  | 1 |  |  | |
|  |  | Vaccinated with 1^st^ dose, inside risk window | 111,740 | 0 | 0.00 | ND | ND | ND | ND | ND | ND | |
|  |  | Vaccinated with 1^st^ dose, outside risk window | 67,273 | 0 | 0.00 | ND | ND | ND | ND | ND | ND | |
|  |  | Vaccinated with 2^nd^ dose, inside risk window | 105,965 | 0 | 0.00 | ND | ND | ND | ND | ND | ND | |
|  |  | Vaccinated with 2^nd^ dose, outside risk window | 105,854 | < 5 | 1.61 | ND | ND | ND | ND | ND | ND | |
| IgA vasculitis | 42 days | *Overall* | 121,555 | < 5 | 1.42 |  |  |  |  |  |  | |
|  |  | Unvaccinated (ref.) | 121,551 | 0 | 0.00 | 1 |  |  | 1 |  |  | |
|  |  | Vaccinated with 1^st^ dose, inside risk window | 111,731 | 0 | 0.00 | ND | ND | ND | ND | ND | ND | |
|  |  | Vaccinated with 1^st^ dose, outside risk window | 67,270 | 0 | 0.00 | ND | ND | ND | ND | ND | ND | |
|  |  | Vaccinated with 2^nd^ dose, inside risk window | 105,956 | 0 | 0.00 | ND | ND | ND | ND | ND | ND | |
|  |  | Vaccinated with 2^nd^ dose, outside risk window | 105,845 | < 5 | 3.21 | ND | ND | ND | ND | ND | ND | |
| Herpes zoster | 28 days | *Overall* | 121,508 | 19 | 13.52 |  |  |  |  |  |  | |
|  |  | Unvaccinated (ref.) | 121,504 | 5 | 11.23 | 1 |  |  | 1 |  |  | |
|  |  | Vaccinated with 1^st^ dose, inside risk window | 111,680 | 0 | 0.00 | ND | ND | ND | ND | ND | ND | |
|  |  | Vaccinated with 1^st^ dose, outside risk window | 101,697 | < 5 | 22.85 | 2.04 | 0.49–8.52 | 0.330 | 1.70 | 0.31–9.34 | 0.544 | |
|  |  | Vaccinated with 2^nd^ dose, inside risk window | 105,908 | < 5 | 12.32 | 1.10 | 0.13–9.40 | 0.932 | 1.09 | 0.10–12.23 | 0.942 | |
|  |  | Vaccinated with 2^nd^ dose, outside risk window | 105,842 | 10 | 15.10 | 1.35 | 0.46–3.94 | 0.588 | 0.88 | 0.19–3.98 | 0.868 | |
| Idiopathic thrombocytopenic | 28 days | *Overall* | 121,538 | < 10 | 5.69 |  |  |  |  |  |  | |
| purpura |  | Unvaccinated (ref.) | 121,534 | < 5 | 6.74 | 1 |  |  | 1 |  |  | |
|  |  | Vaccinated with 1^st^ dose, inside risk window | 111,711 | 0 | 0.00 | ND | ND | ND | ND | ND | ND | |
|  |  | Vaccinated with 1^st^ dose, outside risk window | 101,728 | 0 | 0.00 | ND | ND | ND | ND | ND | ND | |
|  |  | Vaccinated with 2^nd^ dose, inside risk window | 105,936 | 0 | 0.00 | ND | ND | ND | ND | ND | ND | |
|  |  | Vaccinated with 2^nd^ dose, outside risk window | 105,872 | 5 | 7.55 | 1.12 | 0.27–4.69 | 0.877 | 2.45 | 0.39–15.45 | 0.340 | |
| Lymphadenopathy | 14 days | *Overall* | 120,995 | 182 | 130.15 |  |  |  |  |  |  | |
|  |  | Unvaccinated (ref.) | 120,991 | 52 | 117.29 | 1 |  |  | 1 |  |  | |
|  |  | Vaccinated with 1^st^ dose, inside risk window | 111,165 | 6 | 140.83 | 1.20 | 0.52–2.80 | 0.671 | 1.30 | 0.53–3.16 | 0.568 | |
|  |  | Vaccinated with 1^st^ dose, outside risk window | 111,130 | 15 | 86.87 | 0.74 | 0.42–1.32 | 0.306 | 0.84 | 0.43–1.64 | 0.610 | |
|  |  | Vaccinated with 2^nd^ dose, inside risk window | 105,405 | 10 | 247.56 | 2.11 | 1.07–4.15 | 0.031 | 2.37 | 1.09–5.15 | 0.030 | |
|  |  | Vaccinated with 2^nd^ dose, outside risk window | 105,373 | 99 | 141.57 | 1.21 | 0.86–1.69 | 0.272 | 1.30 | 0.77–2.20 | 0.330 | |
| Multisystem inflammatory | 42 days | *Overall* | 121,563 | < 5 | 0.71 |  |  |  |  |  |  | |
| syndrome in children |  | Unvaccinated (ref.) | 121,559 | 0 | 0.00 | 1 |  |  | 1 |  |  | |
|  |  | Vaccinated with 1^st^ dose, inside risk window | 111,737 | 0 | 0.00 | ND | ND | ND | ND | ND | ND | |
|  |  | Vaccinated with 1^st^ dose, outside risk window | 67,272 | 0 | 0.00 | ND | ND | ND | ND | ND | ND | |
|  |  | Vaccinated with 2^nd^ dose, inside risk window | 105,964 | 0 | 0.00 | ND | ND | ND | ND | ND | ND | |
|  |  | Vaccinated with 2^nd^ dose, outside risk window | 105,853 | < 5 | 1.61 | ND | ND | ND | ND | ND | ND | |
| Myocarditis and pericarditis | 28 days | *Overall* | 121,531 | < 41 | 27.04 |  |  |  |  |  |  | |
|  |  | Unvaccinated (ref.) | 121,527 | 8 | 17.96 | 1 |  |  | 1 |  |  | |
|  |  | Vaccinated with 1^st^ dose, inside risk window | 111,700 | < 5 | 23.51 | 1.31 | 0.28–6.16 | 0.733 | 1.87 | 0.31–11.29 | 0.493 | |
|  |  | Vaccinated with 1^st^ dose, outside risk window | 101,714 | 5 | 38.10 | 2.12 | 0.69–6.48 | 0.187 | 3.91 | 0.81–18.77 | 0.089 | |
|  |  | Vaccinated with 2^nd^ dose, inside risk window | 105,926 | 9 | 110.87 | 6.17 | 2.38–16.00 | < 0.001 | 10.25 | 2.36–44.47 | 0.002 | |
|  |  | Vaccinated with 2^nd^ dose, outside risk window | 105,852 | 14 | 21.14 | 1.18 | 0.49–2.81 | 0.713 | 2.84 | 0.61–13.15 | 0.182 | |
| Venous thromboembolic events | 28 days | *Overall* | 121,513 | 26 | 18.50 |  |  |  |  |  |  | |
|  |  | Unvaccinated (ref.) | 121,509 | 6 | 13.47 | 1 |  |  | 1 |  |  | |
|  |  | Vaccinated with 1^st^ dose, inside risk window | 111,684 | < 5 | 35.28 | 2.62 | 0.65–10.47 | 0.173 | 5.07 | 0.96–26.81 | 0.056 | |
|  |  | Vaccinated with 1^st^ dose, outside risk window | 101,699 | < 5 | 15.23 | 1.13 | 0.23–5.60 | 0.881 | 2.39 | 0.31–18.51 | 0.403 | |
|  |  | Vaccinated with 2^nd^ dose, inside risk window | 105,907 | < 5 | 24.64 | 1.83 | 0.37–9.06 | 0.460 | 3.99 | 0.52–30.36 | 0.181 | |
|  |  | Vaccinated with 2^nd^ dose, outside risk window | 105,840 | 13 | 19.63 | 1.46 | 0.55–3.83 | 0.446 | 2.59 | 0.43–15.41 | 0.296 | |

Abbreviations: CI – confidence interval; IRR – incidence rate ratio; mRNA – messenger RNA; ND – not determined

^1^12–15-year-olds: September 6, 2021; 16–17-year-olds: August 23, 2021; 18–19-year-olds: April 5, 2021

^2^Per 100,000 person-years

^3^Adjustment for sex (male or female), health region (North Norway, Central Norway, West Norway, or South-East Norway), and risk group (no or yes) as baseline covariates and three-month calendar period (April–June 2021, July–September 2021, October–December 2021, January–March 2022, April–June 2022, and July–September 2022) as a time-varying covariate

Supplementary Table 4: SCCS results. Adjusted incidence rate ratios of 17 different outcomes between vaccinated and unvaccinated subjects, with associated 95% confidence intervals, based on self-controlled case series (SCCS) analysis of adolescents in Norway aged 12–19 years at the end of 2021 and unvaccinated against SARS-CoV-2 at the beginning of follow-up.

| Outcome | Risk window | Vaccination status | Number of events | Adjusted analysis^1^ | | |
| --- | --- | --- | --- | --- | --- | --- |
|  |  |  |  | IRR | 95% CI | P value |
| Acute appendicitis | 14 days | *Total* | 1,185 |  |  |  |
|  |  | Unvaccinated (ref.) | 267 | 1 |  |  |
|  |  | Vaccinated with 1^st^ dose, inside risk window | 36 | 1.05 | 0.72–1.54 | 0.79 |
|  |  | Vaccinated with 1^st^ dose, outside risk window | 495 | 1.15 | 0.90–1.45 | 0.26 |
|  |  | Vaccinated with 2^nd^ dose, inside risk window | 21 | 1.02 | 0.64–1.63 | 0.94 |
|  |  | Vaccinated with 2^nd^ dose, outside risk window | 366 | 0.97 | 0.78–1.22 | 0.81 |
| Anaphylactic reaction | 2 days | *Total* | < 61 |  |  |  |
|  |  | Unvaccinated (ref.) | 12 | 1 |  |  |
|  |  | Vaccinated with 1^st^ dose, inside risk window | < 5 | 4.92 | 0.59–41.11 | 0.14 |
|  |  | Vaccinated with 1^st^ dose, outside risk window | 26 | 2.02 | 0.74–5.49 | 0.17 |
|  |  | Vaccinated with 2^nd^ dose, inside risk window | < 5 | 2.02 | 0.89–64.17 | 0.06 |
|  |  | Vaccinated with 2^nd^ dose, outside risk window | 16 | 0.77 | 0.29–2.03 | 0.60 |
| Arrhythmia | 28 days | *Total* | 135 |  |  |  |
|  |  | Unvaccinated (ref.) | 39 | 1 |  |  |
|  |  | Vaccinated with 1^st^ dose, inside risk window | < 5 | 0.48 | 0.16–1.41 | 0.18 |
|  |  | Vaccinated with 1^st^ dose, outside risk window | 43 | 1.01 | 0.53–1.91 | 0.99 |
|  |  | Vaccinated with 2^nd^ dose, inside risk window | < 5 | 0.19 | 0.03–1.45 | 0.11 |
|  |  | Vaccinated with 2^nd^ dose, outside risk window | 48 | 1.30 | 0.71–2.39 | 0.39 |
| Arthropathy | 42 days | *Total* | 0 |  |  |  |
|  |  | Unvaccinated (ref.) | 0 | 1 |  |  |
|  |  | Vaccinated with 1^st^ dose, inside risk window | 0 | ND | ND | ND |
|  |  | Vaccinated with 1^st^ dose, outside risk window | 0 | ND | ND | ND |
|  |  | Vaccinated with 2^nd^ dose, inside risk window | 0 | ND | ND | ND |
|  |  | Vaccinated with 2^nd^ dose, outside risk window | 0 | ND | ND | ND |
| Cerebrovascular events | 28 days | *Total* | < 33 |  |  |  |
|  |  | Unvaccinated (ref.) | 10 | 1 |  |  |
|  |  | Vaccinated with 1^st^ dose, inside risk window | < 5 | 1.22 | 0.26–5.72 | 0.80 |
|  |  | Vaccinated with 1^st^ dose, outside risk window | 12 | 0.80 | 0.22–2.92 | 0.74 |
|  |  | Vaccinated with 2^nd^ dose, inside risk window | 0 | ND | ND | ND |
|  |  | Vaccinated with 2^nd^ dose, outside risk window | 6 | 0.44 | 0.11–1.75 | 0.24 |
| Death (all-cause mortality) | 28 days | *Total* |  |  |  |  |
|  |  | Unvaccinated (ref.) |  | 1 |  |  |
|  |  | Vaccinated with 1^st^ dose, inside risk window |  | ND | ND | ND |
|  |  | Vaccinated with 1^st^ dose, outside risk window |  | ND | ND | ND |
|  |  | Vaccinated with 2^nd^ dose, inside risk window |  | ND | ND | ND |
|  |  | Vaccinated with 2^nd^ dose, outside risk window |  | ND | ND | ND |
| Encephalomyelitis and meningitis | 28 days | *Total* | 14 |  |  |  |
|  |  | Unvaccinated (ref.) | < 5 | 1 |  |  |
|  |  | Vaccinated with 1^st^ dose, inside risk window | 0 | ND | ND | ND |
|  |  | Vaccinated with 1^st^ dose, outside risk window | 5 | ND | ND | ND |
|  |  | Vaccinated with 2^nd^ dose, inside risk window | < 5 | ND | ND | ND |
|  |  | Vaccinated with 2^nd^ dose, outside risk window | < 5 | ND | ND | ND |
| Epilepsy and convulsions | 28 days | *Total* | 617 |  |  |  |
|  |  | Unvaccinated (ref.) | 165 | 1 |  |  |
|  |  | Vaccinated with 1^st^ dose, inside risk window | 29 | 0.95 | 0.59–1.52 | 0.83 |
|  |  | Vaccinated with 1^st^ dose, outside risk window | 225 | 1.08 | 0.76–1.56 | 0.66 |
|  |  | Vaccinated with 2^nd^ dose, inside risk window | 30 | 1.59 | 0.99–2.55 | 0.05 |
|  |  | Vaccinated with 2^nd^ dose, outside risk window | 168 | 1.08 | 0.77–1.52 | 0.66 |
| Facial nerve palsy | 28 days | *Total* | 91 |  |  |  |
|  |  | Unvaccinated (ref.) | 34 | 1 |  |  |
|  |  | Vaccinated with 1^st^ dose, inside risk window | < 5 | 0.89 | 0.27–2.96 | 0.85 |
|  |  | Vaccinated with 1^st^ dose, outside risk window | 20 | 0.96 | 0.37–2.48 | 0.93 |
|  |  | Vaccinated with 2^nd^ dose, inside risk window | < 5 | 0.64 | 0.13–3.04 | 0.57 |
|  |  | Vaccinated with 2^nd^ dose, outside risk window | 31 | 1.11 | 0.49-2.52 | 0.81 |
| Guillain-Barré syndrome | 42 days | *Total* | 6 |  |  |  |
|  |  | Unvaccinated (ref.) | 0 | 1 |  |  |
|  |  | Vaccinated with 1^st^ dose, inside risk window | 0 | ND | ND | ND |
|  |  | Vaccinated with 1^st^ dose, outside risk window | < 5 | ND | ND | ND |
|  |  | Vaccinated with 2^nd^ dose, inside risk window | 0 | ND | ND | ND |
|  |  | Vaccinated with 2^nd^ dose, outside risk window | < 5 | ND | ND | ND |
| IgA vasculitis | 42 days | *Total* | < 17 |  |  |  |
|  |  | Unvaccinated (ref.) | < 5 | 1 |  |  |
|  |  | Vaccinated with 1^st^ dose, inside risk window | 0 | ND | ND | ND |
|  |  | Vaccinated with 1^st^ dose, outside risk window | 7 | 0.63 | 0.07–5.44 | 0.67 |
|  |  | Vaccinated with 2^nd^ dose, inside risk window | 0 | ND | ND | ND |
|  |  | Vaccinated with 2^nd^ dose, outside risk window | 5 | 1.48 | 0.12–18.91 | 0.76 |
| Herpes zoster | 28 days | *Total* | 80 |  |  |  |
|  |  | Unvaccinated (ref.) | 18 | 1 |  |  |
|  |  | Vaccinated with 1^st^ dose, inside risk window | < 5 | 1.56 | 0.25–9.67 | 0.63 |
|  |  | Vaccinated with 1^st^ dose, outside risk window | 29 | 2.26 | 0.56–9.19 | 0.25 |
|  |  | Vaccinated with 2^nd^ dose, inside risk window | < 5 | 1.65 | 0.16–16.80 | 0.67 |
|  |  | Vaccinated with 2^nd^ dose, outside risk window | 30 | 4.12 | 1.10–15.4 | 0.04 |
| Idiopathic thrombocytopenic | 28 days | *Total* | 24 |  |  |  |
| purpura |  | Unvaccinated (ref.) | 6 | 1 |  |  |
|  |  | Vaccinated with 1^st^ dose, inside risk window | < 5 | 0.75 | 0.18–10.66 | 0.75 |
|  |  | Vaccinated with 1^st^ dose, outside risk window | 8 | 0.89 | 0.12–6.78 | 0.91 |
|  |  | Vaccinated with 2^nd^ dose, inside risk window | < 5 | 1.21 | 0.10-13.89 | 0.88 |
|  |  | Vaccinated with 2^nd^ dose, outside risk window | 7 | 1.09 | 0.19–6.41 | 0.92 |
| Lymphadenopathy | 14 days | *Total* | 651 |  |  |  |
|  |  | Unvaccinated (ref.) | 152 | 1 |  |  |
|  |  | Vaccinated with 1^st^ dose, inside risk window | 15 | 0.79 | 0.45–1.40 | 0.43 |
|  |  | Vaccinated with 1^st^ dose, outside risk window | 249 | 1.00 | 0.73–1.38 | 0.98 |
|  |  | Vaccinated with 2^nd^ dose, inside risk window | 22 | 2.04 | 1.24–3.35 | 0.01 |
|  |  | Vaccinated with 2^nd^ dose, outside risk window | 213 | 1.08 | 0.80–1.47 | 0.61 |
| Multisystem inflammatory | 42 days | *Total* | 21 |  |  |  |
| syndrome in children |  | Unvaccinated (ref.) | 6 | 1 |  |  |
|  |  | Vaccinated with 1^st^ dose, inside risk window | < 5 | 1.14 | 0.17–7.52 | 0.89 |
|  |  | Vaccinated with 1^st^ dose, outside risk window | 8 | 0.47 | 0.09–2.62 | 0.39 |
|  |  | Vaccinated with 2^nd^ dose, inside risk window | 0 | NA | NA | NA |
|  |  | Vaccinated with 2^nd^ dose, outside risk window | < 5 | 1.33 | 0.11–16.65 | 0.82 |
| Myocarditis and pericarditis | 28 days | *Total* | < 68 |  |  |  |
|  |  | Unvaccinated (ref.) | 13 | 1 |  |  |
|  |  | Vaccinated with 1^st^ dose, inside risk window | < 5 | 1.20 | 0.30–4.84 | 0.80 |
|  |  | Vaccinated with 1^st^ dose, outside risk window | 17 | 1.60 | 0.53–4.84 | 0.40 |
|  |  | Vaccinated with 2^nd^ dose, inside risk window | 11 | 5.88 | 2.11–16.40 | <0.001 |
|  |  | Vaccinated with 2^nd^ dose, outside risk window | 22 | 1.14 | 0.45–2.93 | 0.78 |
| Venous thromboembolic events | 28 days | *Total* | < 71 |  |  |  |
|  |  | Unvaccinated (ref.) | 11 | 1 |  |  |
|  |  | Vaccinated with 1^st^ dose, inside risk window | 5 | 1.05 | 0.35–3.16 | 0.93 |
|  |  | Vaccinated with 1^st^ dose, outside risk window | 20 | 1.04 | 0.42–2.62 | 0.93 |
|  |  | Vaccinated with 2^nd^ dose, inside risk window | < 5 | 0.81 | 0.22–3.02 | 0.75 |
|  |  | Vaccinated with 2^nd^ dose, outside risk window | 30 | 0.98 | 0.45–2.14 | 0.95 |

^1^Adjustment for seasonality, defined as quarterly time periods (January–March, April–June, July–September, and October–December)

# *Supplementary Table 5: Overview of earlier studies.*

| **Pubmed ID** | | **Title** | **First Author** | **Study Description** | **Age-group** | **Vaccine** | **Grouped into** |
| --- | --- | --- | --- | --- | --- | --- | --- |
|  | | **Studies on other topics or vaccines (n=94)** |  |  |  |  |  |
| 33113210 | | Is there a role for childhood vaccination against COVID-19? | Eberhardt CS | Study summarizing vaccine platforms, and debates practical and ethical consideration for vaccination in children | Children | COVID-19 vaccines | Other |
| 33332292 | | The Advisory Committee on Immunization Practices' Interim Recommendation for Use of Pfizer-BioNTech COVID-19 Vaccine - United States, December 2020 | Oliver SE | Advisory committee on immunizations interim recommendation for use of BNT162b2 in the US | ≥16 | BNT162b2 | Other |
| 33382675 | | The Advisory Committee on Immunization Practices' Interim Recommendation for Use of Moderna COVID-19 Vaccine - United States, December 2020 | Oliver SE | Advisory committee on immunization interim recommendation for use of mRNA-1273 in ≥18 in the US | 18+ | mRNA-1273 | Other |
| 33394522 | | Clinical manifestations and impact on daily life of allergy to polyethylene glycol (PEG) in ten patients | Bruusgaard-Mouritsen MA | Study investigating clinical manifestations, time to diagnosis and impact of PEG allergy diagnosis | 18-64 | - | Other |
| 33612319 | | [In subjects 16 years of age and older, is messenger RNA vaccine BNT162b2 against COVID-19 effective and safe?] | Lanthier L | Letter discussing if BNT162b2 vaccine is safe and effective in subjects 16+ years of age | 16+ | BNT162b2 | Other |
| 33643776 | | Severe allergic reactions after COVID-19 vaccination with the Pfizer/BioNTech vaccine in Great Britain and USA: Position statement of the German Allergy Societies: Medical Association of German Allergologists (AeDA), German Society for Allergology and Clinical Immunology (DGAKI) and Society for Pediatric Allergology and Environmental Medicine (GPA) | Klimek L | Position statement that potential covid-vaccinees must be informed of risk to severe allergic/anaphylactic reactions and questioned on history of these, and vaccine additives must be queried | - | BNT162b2 | Other |
| 33882218 | | Preliminary Findings of mRNA Covid-19 Vaccine Safety in Pregnant Persons | Shimabukuro TT | Study on vaccine safety in pregnant women | 16-54 | mRNA covid vaccines | Other, wrong age-group, Vaccine efficacy |
| 33898162 | | Practical handling of allergic reactions to COVID-19 vaccines: A position paper from German and Austrian Allergy Societies AeDA, DGAKI, GPA and ÖGAI | Klimek L | Manuscript with recommendations for handling allergic reaction to covid vaccines | - | BNT162b2, mRNA-1273 | Other |
| 33956784 | | Safety Monitoring of the Janssen (Johnson & Johnson) COVID-19 Vaccine - United States, March-April 2021 | Shay DK | Manuscript describing the safety monitoring of Janssen vaccine in the US reviewing VAERS and v-safe data | 18+ | Ad26.COV2.S | Other Vaccine, wrong age-group |
| 34028219 | | [THE NEED FOR REDUCING DISPARITIES IN SARS-COV-2 IMMUNIZATION: THE ULTRAORTHODOX AND ARAB POPULATIONS IN ISRAEL] | Ber I | Study reviewing factors for vaccine acceptance, presenting low vaccine uptake in ultraorthodox and arab israeli populations | - | COVID-19 vaccines | Other |
| 34052265 | | Optimizing investigation of suspected allergy to polyethylene glycols | Bruusgaard-Mouritsen MA | Study evaluating skin prick test results to different polyethylene glycols used in mRNA vaccines | 16-63 | - | Other |
| 34237049 | | Use of mRNA COVID-19 Vaccine After Reports of Myocarditis Among Vaccine Recipients: Update from the Advisory Committee on Immunization Practices - United States, June 2021 | Gargano JW | Advisory committee on immunization practices statement on myocarditis after vaccination in the US | 12+ | BNT162b2, mRNA-1273 | Other |
| 34302866 | | Crossing the Rubicon: A fine line between waiting and vaccinating adolescents against COVID-19 | Ladhani SN | Study discussing pros and cons of vaccinating adolescents against covid | - | COVID-19 vaccines in general | Other |
| 34332972 | | Important Insights into Myopericarditis after the Pfizer mRNA COVID-19 Vaccination in Adolescents | Long SS | Editorial describing several studies with adolescent myocarditis cases | 12-17 | BNT162b2 | Review, case report/series, Other |
| 34336774 | | SARS-CoV-2 mRNA Vaccine Attitudes as Expressed in U.S. FDA Public Commentary: Need for a Public-Private Partnership in a Learning Immunization System | Weitzman ER | Study on vaccine attitudes in the public | - | mRNA COVID-19 vaccines | Other |
| 34375696 | | Myocarditis and Pericarditis After COVID-19 mRNA Vaccination: Practical Considerations for Care Providers | Luk A | Commentary providing real-time pragmatic framework for cardiovascular care providers | - | COVID-19 mRNA vaccines | Other |
| 34378087 | | Safety and immunogenicity of an mRNA-lipid nanoparticle vaccine candidate against SARS-CoV-2 : A phase 1 randomized clinical trial | Kremsner PG | Clinical trial of CVnCoV | 18-60 | CVnCoV | Clinical Trial, other vaccine |
| 34384875 | | COVID-19 vaccination in Israel | Muhsen K | Manuscript describing the covid-19 vaccination in israel | 10-90+ | BNT162b2 | Other |
| 34473597 | | Reply letter to "safety of SARS-Cov-2 vaccines administration for adult patients with hereditary fructose intolerance" | Saborido-Fiaño R | Comment/reply letter discussing if vaccines are safe for pediatric patients with hereditary fructose intolerance | Children | BNT162b2, mRNA-1273 | Other |
| 34533570 | | Assessment of Allergic and Anaphylactic Reactions to mRNA COVID-19 Vaccines With Confirmatory Testing in a US Regional Health System | Warren CM | Study characterizing immune mechanisms underlying allergic reactions to covid mRNA vaccines | Mean 40.9, SD 10.3 | mRNA Covid vaccines | Other, case-series, Clinical characteristics |
| 34544112 | | In brief: Myocarditis with the Pfizer/BioNTech and Moderna COVID-19 vaccines | No authors listed | brief letter discussing the data and risk of myocarditis after mRNA vaccination | 12-29 | BNT162b2, mRNA-1273 | Other, Review |
| 34538306 | | COVID-19 in Children: Clinical Manifestations and Pharmacologic Interventions Including Vaccine Trials | Galindo R | Review of clinical manifestations of covid-19 in children and interventions | Children | BNT162b2, mRNA-1273, ChAdOx1, Ad26.COV2.S, NVX-CoV2373 | Review, Other |
| 34540594 | | Why are we vaccinating children against COVID-19? | Kostoff RN | Retracted manuscript examining issues related to covid vaccination in children |  | COVID-19 vaccines | Retracted, Other |
| 34555007 | | Use of Pfizer-BioNTech COVID-19 Vaccine in Persons Aged ≥16 Years: Recommendations of the Advisory Committee on Immunization Practices - United States, September 2021 | Dooling K | Recommendations on use of BNT162b2 in 16+ from advisory committee on immunization practices in the US | ≥16 | BNT162B2 | Other |
| 34591835 | | Safety Monitoring of an Additional Dose of COVID-19 Vaccine - United States, August 12-September 19, 2021 | Hause AM | Study describing safety monitoring of additional dose of covid vaccine using v-safe, a smartphone app | 0-85+ | mRNA-1273, BNT162b2, Ad26.COV2.S | Other, local/systemic reactions, between-group comparison |
| 34620243 | | COVID-19 vaccination intention and vaccine characteristics influencing vaccination acceptance: a global survey of 17 countries | Wong LP | Survey of covid-19 vaccination intention and vaccine characteristics influencing vaccination acceptance | 18+ | COVID-19 vaccines | Other |
| 34707073 | | Does immunosuppressive property of non-steroidal anti-inflammatory drugs (NSAIDs) reduce COVID-19 vaccine-induced systemic side effects? | Kazama I | Survey study on use of NSAIDs to reduce systemic side effects | 18-22 | BNT162b2 | Other, wrong age-group |
| 34735422 | | The Advisory Committee on Immunization Practices' Interim Recommendations for Additional Primary and Booster Doses of COVID-19 Vaccines - United States, 2021 | Mbaeyi S | Advisory committee on immunization practice recommendations for primary and booster doses in the US | 18+ | BNT162b2, mRNA-1273 | Other |
| 34887571 | | Myocarditis after COVID-19 mRNA vaccination: clinical observations and potential mechanisms | Heymans S | Comment describing clinical observations, potential mechanisms of myocarditis, recommending vaccination | - | COVID-19 mRNA | Other, clinical characteristics |
| 34906965 | | Can families believe the accuracy of websites' information regarding COVID-19 vaccines' side effects? | Harvey-Nguyen L | letter on parents views | - | - | Other |
| 34916063 | | Vaccine-Induced Thrombotic Thrombocytopenia Due to Coronavirus Disease 2019 Vaccine From a Deceased Donor: A Case Report | Guditi S | Case report on vaccine-induced thrombotic thrombocytopenia, and clinical outcome on recipients of her organs | 18 | ChAdOx | Case report/series, other vaccine |
| 34916217 | | Myocarditis after vaccination against covid-19 | Gellad WF | Editorial on Husby et al myocarditis study (PMID 34916207) | - | - | Other |
| 34952008 | | mRNA Coronavirus Disease 2019 Vaccine-Associated Myopericarditis in Adolescents: A Survey Study | Kohli U | Survey study of myo/pericarditis in adolescents to assess variability of treatment | 12-18 | mRNA COVID-19 vaccines | Other |
| 34965462 | | Comparing self-reported reactogenicity between adolescents and adults following the use of BNT162b2 (Pfizer-BioNTech) messenger RNA COVID-19 vaccine: a prospective cohort study | Chan EWW | Cohort study on age differences in self-report | 12-17 vs. 18-59 and 60+ | BNT162b2 | Between-group comparison, Other |
| 34990445 | | Receipt of COVID-19 Vaccine During Pregnancy and Preterm or Small-for-Gestational-Age at Birth - Eight Integrated Health Care Organizations, United States, December 15, 2020-July 22, 2021 | Lipkind HS | Study of covid vaccination during pregnancy and preterm or small for gestational age in offspring | mean 32.3 | BNT162b2, mRNA-1273, Ad26.COV2.S | Other, wrong age-group |
| 35012590 | | Reasons in favour of universal vaccination campaign against COVID-19 in the pediatric population | Principi N | Manuscript explaining the reasons for overcoming vaccine hesitancy in pediatric population | 5-17 | COVID-19 vaccines | Other |
| 35062742 | | The Burden of COVID-19 in Children and Its Prevention by Vaccination: A Joint Statement of the Israeli Pediatric Association and the Israeli Society for Pediatric Infectious Diseases | Stein M | Joint statement on the burden of covid-19 in children and its prevention by vaccination | 5-11 | BNT162b2 | Other, Review |
| 35090809 | | Humoral response to SARS-CoV-2 adenovirus vector vaccination (ChAdOx1 nCoV-19 [AZD1222]) in heart transplant recipients aged 18 to 70 years of age | Tanner R | Study on humoral response to ChAdOx vaccination in adult heart transplant recipients | 18-70 (mean 51) | ChAdOx1 | Wrong age-group, clinical characteristics, specific group, Other vaccine |
| 35156705 | | BNT162b2 Vaccine-Associated Myo/Pericarditis in Adolescents: A Stratified Risk-Benefit Analysis | Krug A | Study on BNT162b2-associated myo/pericarditis risk benefit-analysis in adolescents using VAERS data | 12-17 | BNT162b2 | Other, between-group comparison, no comparator |
| 35180029 | | Myocarditis and pericarditis following mRNA COVID-19 vaccination in younger patients: is there a shared thread? | Mormile R | Study postulating possible mechanisms/hypothesis of pathogenesis of vaccine-related myo/pericarditis | - | mRNA COVID-19 vaccines | Other |
| 35201615 | | COVID-19 vaccination in children and adolescents aged 5 years and older undergoing treatment for cancer and non-malignant haematological conditions: Australian and New Zealand Children's Haematology/Oncology Group consensus statement | Furlong E | Consensus statement on covid vaccination in children and adolescents undergoing cancer treatment and non-malignant haemotological conditions | 5+ | BNT162b2, mRNA-1273 | Other, specific group |
| 35266090 | | The benefit of vaccination against COVID-19 outweighs the potential risk of myocarditis and pericarditis | Klamer TA | Study arguing that benefit of vaccination outweighs potential myo/pericarditis risk in adolescents and adults | - | BNT162b2, mRNA-1273 | Other |
| 35298454 | | The Advisory Committee on Immunization Practices' Recommendation for Use of Moderna COVID-19 Vaccine in Adults Aged ≥18 Years and Considerations for Extended Intervals for Administration of Primary Series Doses of mRNA COVID-19 Vaccines - United States, February 2022 | Wallace M | Practice recommendations for use of moderna vaccine in adults in the US, also discusses extended intervals for administration of mRNA COVID-19 vaccines | 18+ | mRNA-1273 | Other |
| 35358170 | | Effectiveness of Homologous and Heterologous COVID-19 Booster Doses Following 1 Ad.26.COV2.S (Janssen [Johnson & Johnson]) Vaccine Dose Against COVID-19-Associated Emergency Department and Urgent Care Encounters and Hospitalizations Among Adults - VISION Network, 10 States, December 2021-March 2022 | Natarajan K | Study on effectiveness of homo- and heterologous booster doses against ER, urgent care and hospitalizations in adults | >18 | Ad26.COV2.S | Vaccine efficacy, Other vaccine |
| 35370016 | | Benefit-risk assessment of COVID-19 vaccine, mRNA (Comirnaty) for age 16-29 years | Funk PR | Study on benefit-risk assessment of BNT162b2 for ages 16-29 | 16-29 | BNT162b2 | Other |
| 35387459 | | Ethical and legal requirements for vaccination against COVID-19 | Franc A | Manuscript discussing ethical and legal requirements for vaccination against covid-19 | - | COVID-19 vaccines | Other |
| 35404496 | | Risk of myopericarditis following COVID-19 mRNA vaccination in a large integrated health system: A comparison of completeness and timeliness of two methods | Sharff KA | Study on different methods and their completeness and timeliness when investigating myocarditis following covid-19 mRNA vaccination | 12-39 | BNT162b2, mRNA-1273 | Other, specific disease |
| 35484992 | | Comment on: COVID-19 vaccine (mRNA BNT162b2) and COVID-19 infection-induced thrombotic thrombocytopenic purpura in adolescents | Sookaromdee P | Comment on case report by Vorster et al. (PMID 35373880) | - | - | Other |
| 35607748 | | Paving the Way Towards Precision Vaccinology: The Paradigm of Myocarditis After Coronavirus Disease 2019 (COVID-19) Vaccination | Lagousi T | Review of myocarditis epidemiology following mRNA vaccination and potential mechanisms | - | SARS-CoV-2 mRNA vaccines | Review, Other |
| 35666513 | | Analysis of Myocarditis Among 252 Million mRNA-1273 Recipients Worldwide | Straus W | Study reviewing myo/pericarditis reports in the Moderna global safety database after mRNA-1273 vaccination - spontaneous reports | 12-15, 16-17, 18-29, 30-39, 40-49, 50-64 ,65-74, 75+ | mRNA-1273 | Other population comparator, historical comparator, single disease |
| 35731908 | | Maternal Vaccination and Risk of Hospitalization for Covid-19 among Infants | Halasa NB | Study on maternal vaccination and risk of covid hospitalization among infants | <6 months | BNT162b2, mRNA-1273 | Wrong age-group, Other, Vaccine efficacy |
| 35795703 | | A Community-Informed Approach to COVID-19 Vaccine Roll-Out in Under-served Areas in Chicago | DiVirgilio L | Study describing covid vaccine roll-out in under-resourced urban area in Chicago | 18+; mean age 47 | BNT162b2, mRNA-1273 | Other |
| 35816282 | | SARS-CoV-2 vaccines in children and adolescents: Can immunization prevent hospitalization? | Liang KH | Review with topic if covid immunization can prevent hospitalization | - | BNT162b2, mRNA-1273 | Other, Review |
| 35833128 | | Myocarditis Following COVID-19 Vaccine Use: Can It Play a Role for Conditioning Immunization Schedules? | Esposito S | Review on myo/pericarditis after covid vaccination | - | COVID-19 vaccines | Other, Review |
| 36030299 | | A Prospective Observational Study on BBV152 Coronavirus Vaccine Use in Adolescents and Comparison with Adults: Interim Results of the First Real-World Safety Analysis | Kaur U | Prospective study on safety of BBV152 vaccine | 15-18, 19+ | BBV152 | Other vaccine |
| 36116762 | | Vaccinating children: The pros and cons | Cohen R | Article on pros and cons of vaccinating children | Children | mRNA vaccines | Other |
| 36130187 | | Associations of Immunogenicity and Reactogenicity After Severe Acute Respiratory Syndrome Coronavirus 2 mRNA-1273 Vaccine in the COVE and TeenCOVE Trials | Siangphoe U | Study investigating the association between immunogenicity and reactogenicity. high Ab titers associated with systemic adverse reactions | 12-100 | mRNA-1273 | Other |
| 36168404 | | Risk factors for SARS-CoV-2 infection after primary vaccination with ChAdOx1 nCoV-19 or BNT162b2 and after booster vaccination with BNT162b2 or mRNA-1273: A population-based cohort study (COVIDENCE UK) | Vivaldi G | Population-based cohort study on breakthrough infections and risk factors of these after ChAdOx or BNT vaccination | 16+ | ChAdOx1, BNT162b2, mRNA-1273 | Other, between-group comparison, wrong age-group |
| 36231528 | | COVID-19 Vaccine in Inherited Metabolic Disorders Patients: A Cross-Sectional Study on Rate of Acceptance, Safety Profile and Effect on Disease | Tummolo A | Cross-sectional study on acceptancy, safety and effect on disease of covid-19 vaccine in patients with inherited metabolic disorders | 12-61 | BNT162b2, mRNA-1273, ChAdOx1 | Other, specific group, wrong age-group |
| 36282106 | | The challenges of the pandemic and the vaccination against covid-19 in pediatric patients with kidney disease | Soeiro EMD | Paper on latest knowledge about vaccination against covid-19 for children with Kidney disease | Children and adolescents | BNT162b2, CoronaVac | Other, specific group, review |
| 36496293 | | Acute macular neuroretinopathy and COVID-19 vaccination: Case report and literature review | Fekri S | Case report and literature review of acute macular neuroretinopathy following covid-19 vaccination. | 18-54 | BBIBP-CorV | other vaccine, case report/series, wrong age-group |
| 36600579 | | COVID-19 vaccine boosters for young adults: a risk benefit assessment and ethical analysis of mandate policies at universities | Bardosh K | Risk-benefit and ethical analysis of appropriateness of booster mandates for 18-29 year olds | 18-29 | BNT162b2, mRNA-1273 | Other |
| 36769279 | | Detection of SARS-CoV-2-Specific Antibodies in Human Breast Milk and Their Neutralizing Capacity after COVID-19 Vaccination: A Systematic Review | Nicolaidou V | Systemic review on Sars-Cov-2 antibodies in breast milk and their neutralizing capacity after vaccination | >18 | BNT162b2, mRNA-1273, ChAdOx1 | Other, clinical characteristics, review |
| 36881800 | | COVID-19 Vaccines for Children: An Update | Rosenberg A | Manuscript recommending COVID-19 vaccination for children and adolescents, opinion/statement | - | COVID-19 vaccines | Review/Other |
| 37012151 | | Role of spontaneous reporting in investigating the relationship between mRNA COVID-19 vaccines and myocarditis: The French perspective | Salvo F | Study on the role of the spontaneous reporting system in detection, assessment, and quantification of vaccine-related myocarditis in France | 18-76 | mRNA vaccines | Wrong age-group, other |
| 37216958 | | Safety, immunogenicity, and efficacy of the mRNA vaccine CS-2034 as a heterologous booster versus homologous booster with BBIBP-CorV in adults aged ≥18 years: a randomised, double-blind, phase 2b trial | Wu JD | Trial on CS-2034 booster vs BBIBP-CorV in adults | 18-59, 60+ | CS-2034 vs BBiBP-CorV | Clinical Trial, Other vaccine |
| 37236995 | | Phase I randomized, observer-blinded, placebo-controlled study of a SARS-CoV-2 mRNA vaccine PTX-COVID19-B | Martin-Orozco N | Report on the pre-clinical development and evaluation of PTX-COVID19-B | - | PTX-XOVID19-B | Clinical trial, Other vaccine |
| 37270340 | | "Acute psychosis induced by mRNA-based COVID-19 vaccine in adolescents: A pediatric case report": Comment | Kleebayoon A | Comment on PMID 36641359 |  | - | Case report/series, Other |
| 37324525 | | Benefit-risk assessment of Covid-19 vaccine, MRNA (MRNA-1273) for males age 18-64 years | Yogurtcu ON | Risk-benefit assessment for mRNA-1273 for males 18-64 years | 18-64 | mRNA-1273 | Other |
| 37360870 | | Benefits v. risks of COVID-19 vaccination: an examination of vaccination policy impact on the occurrence of myocarditis and pericarditis | Carleton BC | Commentary of the policy considerations on the occurrence of myocarditis and pericarditis | - | mRNA COVID-19 vaccines | Other |
| 37410982 | | Timely Second-Dose Completion of mRNA COVID-19 Vaccination at Community-Based and Mobile Vaccine Clinics in Maryland | Parent C | Study on factors associated with timely second-dose completion | 16+ | BNT162b2, mRNA-1273 | Other, Clinical characteristics |
| 37535398 | | Tixagevimab/cilgavimab for preventing COVID-19 during the Omicron surge: retrospective analysis of National Veterans Health Administration electronic data | Young-Xu Y | Target trial emulation on effectiveness of tixagevimab/cilgavimab on preventing covid-19 (omicron) | ≥18 | - | Other, specific group, effectiveness |
| 37599140 | | Simultaneous administration of mRNA COVID-19 bivalent booster and influenza vaccines | Kenigsberg TA | Descriptive study on simultaneous administration of mRNA covid booster and influenza | >6 months | mRNA COVID-19 bivalent booster + influenza | Other |
| 37697268 | | West Australian parents' views on vaccinating their children against COVID-19: a qualitative study | Carlson SJ | Qualitative study on parents views on vaccinating their children | 5-17 | COVID-19 vaccines | Other |
| 37739888 | | A Brighton Collaboration standardized template with key considerations for a benefit/risk assessment for the Novavax COVID-19 Vaccine (NVX-CoV2373), a recombinant spike protein vaccine with Matrix-M adjuvant to prevent disease caused by SARS-CoV-2 viruses | Wilkinson B | Brighton collaboration review on Novavax vaccine | - | NVX-CoV2373 | Other, Other vaccine |
| 37792452 | | Risk Factors for Not Completing a 2-Dose Primary Series of Messenger RNA COVID-19 Vaccination in a Large Health Care System in Southern California: Retrospective Cohort Study | Xu S | Study on risk factors for not completing a 2-dose primary series of mRNA vaccination | 18+ | mRNA COVID-19 vaccines | Other, Wrong age-group |
| 37879379 | | Incidence of Severe Adverse Drug Reactions to Ultrasound Enhancement Agents in a Contemporary Echocardiography Practice | Ali MT | Study on adverse drug reaction to ultrasound enhancement agents Definity and Lumason |  | mRNA-1273, BNT162b2 | Other |
| 38111106 | | Safety and immunogenicity of primary vaccination with a SARS-CoV-2 mRNA vaccine (SYS6006) in Chinese participants aged 18 years or more: Two randomized, observer-blinded, placebo-controlled and dose-escalation phase 1 clinical trials | Chen GL | Clinical trial of SYS6006 vaccine | 18-59, 60+ | SYS6006 | Clinical Trial, Other vaccine |
| 38141632 | | Immunogenicity and safety of a booster dose of a self-amplifying RNA COVID-19 vaccine (ARCT-154) versus BNT162b2 mRNA COVID-19 vaccine: a double-blind, multicentre, randomised, controlled, phase 3, non-inferiority trial | Oda Y | Non-inferiority trial of ARCT-154 booster dose, also immunogenicity and safety | ≥18 | ARCT-154 | Clinical Trial, wrong age-group, local/systemic reactions, other |
| 38169007 | | Vaccination against COVID-19 - risks and benefits in children | Munro APS | Review of potential benefits and costs of vaccinating children | Children | COVID-19 vaccines | Other |
| 38287068 | | Phase II prefusion non-stabilised Covid-19 mRNA vaccine randomised study | Puthanakit T | Clinical trial on ChulaCov19 vaccine | 18-59 | ChulaCov19 | Clinical trial, Other vaccine, local/systemic reactions |
| 38584057 | | Immunogenicity and safety of Sinovac-CoronaVac booster vaccinations in 12-17- year-olds with clinically significant reactions from Pfizer-BNT162b2 vaccination | Chong CY | Study on 12–17-year-olds receiving Sinovac due to reaction to BNT162b2 | 12-17 | Sinovac-CoronaVac booster | Other vaccine, Specific group |
| 38641494 | | COVID-19 vaccine uptake among children and adolescents in Norway: A comprehensive registry-based cohort study of over 800,000 individuals | Orangzeb S | Study on vaccine uptake in Norway | <18 | mRNA vaccine | Other |
| 38744844 | | Safety, immunogenicity and efficacy of the self-amplifying mRNA ARCT-154 COVID-19 vaccine: pooled phase 1, 2, 3a and 3b randomized, controlled trials | Hồ NT | Clinical trial of ARCT-154 vaccine | 18+ | ARCT-154 | Clinical trial, other |
| 38761778 | | Safety and immunogenicity of COReNAPCIN, a SARS-CoV-2 mRNA vaccine, as a fourth heterologous booster in healthy Iranian adults: A double-blind, randomized, placebo-controlled, phase 1 clinical trial with a six-month follow-up | Salehi M | Clinical Trial COReNPACIN booster | 18-50 | COReNAPCIN | Clinical Trial, other vaccine |
| 38783823 | | Global and regional burden of vaccine-associated facial paralysis, 1967-2023: Findings from the WHO international pharmacovigilance database | Jeong YD | Global estimates of facial paralysis from the World Health Organization international pharmacovigilance database | any | 16 vaccines | No comparator, between-group comparison, Other |
| 38844841 | | Genome-wide association study of BNT162b2 vaccine-related myocarditis identifies potential predisposing functional areas in Hong Kong adolescents | She CH | GWAS of BNT162b2-vaccine related myocarditis in adolescents | 12-17 | BNT162b2 | Other |
| 38860446 | | The safety, immunogenicity, and efficacy of heterologous boosting with a SARS-CoV-2 mRNA vaccine (SYS6006) in Chinese participants aged 18 years or more: a randomized, open-label, active-controlled phase 3 trial | Zhou C | Trial of heterologous boosting with SYS6006, a SARS-CoV-2 mRNA vaccine | 18+ | SYS6006 | Clinical Trial, other vaccine |
| 38859751 | | Global estimates on the reports of vaccine-associated myocarditis and pericarditis from 1969 to 2023: Findings with critical reanalysis from the WHO pharmacovigilance database | Lee S | Global estimates of myo/pericarditis from World Health Organization international pharmacovigilance database (VigiBase?) | any | 19 vaccines | No comparator, between-group comparison, Other |
| 39171515 | | Global burden of vaccine-associated rheumatic diseases and their related vaccines, 1967-2023: A comprehensive analysis of the international pharmacovigilance database | Oh J | Study reporting global estimates of vaccine-associated rheumatic diseases (vs. all other medicinal products) from VigiBase | any | all vaccines, for COVID-19 grouped into mRNA, Ad5-vectored and Inactivated whole-virus | Other |
| 39352150 | | Adverse event following immunisation of adsorbed-inactivated Coronavac (Sinovac) and ChAdOx1 nCOV-19 (Astra Zeneca) of COVID-19 vaccines | Simatupang A | Study using self-reported questionnaires to compare CoronaVac and ChAdOx1 | not given | CoronaVac, ChAdOx1 | between-group comparison, other vaccine, local/systemic reactions |
| 39397784 | | A novel orf virus vector-based COVID-19 booster vaccine shows cross-neutralizing activity in the absence of anti-vector neutralizing immunity | Klinkardt U | Trial of novel virus vector vaccine Prime-2-CoV_Beta | 18-55 and 65-85 | Prime-2-CoV_Beta | Clinical Trial, Other vaccine, wrong age-group |
| 39412841 | | COVID-19 Vaccine Preferences in General Populations in Canada, Germany, the United Kingdom, and the United States: Discrete Choice Experiment | Salisbury D | Study reporting results of discrete choice experiment on vaccine preferences | ≥ 18 | 11 hypothetical vaccine profiles | Other |
| 39668765 | | Nuvaxovid NVX-CoV2373 vaccine safety profile: real-world data evidence after 100,000 doses, Australia, 2022 to 2023 | Clothier HJ | AEFIs spontaneously reported for Nuvaxovid in SAFEVAC | 6-97 (report in 10-year intervalls) | NVX-CoV2373 | Other vaccine, Wrong age-group, clinical characteristics |
|  | | **Studies on vaccine efficacy (n=23)** |  |  |  |  |  |
| 34049688 | | Efficacy of COVID-19 vaccines: From clinical trials to real life | Deplanque D | Study summarizing vaccine effectiveness of COVID-19 vaccines | - | COVID-19 vaccines | Vaccine efficacy, Review |
| 34417165 | | Effectiveness of BNT162b2 and mRNA-1273 covid-19 vaccines against symptomatic SARS-CoV-2 infection and severe covid-19 outcomes in Ontario, Canada: test negative design study | Chung H | Vaccine effectiveness test-negative study on BNT162b2 and mRNA-1273 vs. symptomatic covid and severe covid outcomes | 16+ | BNT162b2, mRNA-1273 | Wrong age-group, vaccine efficacy, between-group comparison |
| 34437524 | | Sustained Effectiveness of Pfizer-BioNTech and Moderna Vaccines Against COVID-19 Associated Hospitalizations Among Adults - United States, March-July 2021 | Tenforde MW | Effectiveness study of mRNA-1273 and BNT162b2 vaccines against COVID-19 associated hospitalizations | ≥18 | BNT162b2, mRNA-1273 | Wrong age-group, vaccine efficacy |
| 34493859 | | Effectiveness of the BNT162b2 mRNA COVID-19 vaccine in pregnancy | Dagan N | Effectiveness study of BNT162b2 in pregnancy | 16+ | BNT162b2 | Vaccine efficacy |
| 34551225 | | Efficacy of the mRNA-1273 SARS-CoV-2 Vaccine at Completion of Blinded Phase | El Sahly HM | Clinical trial of mRNA-1273 efficacy in preventing Covid-19 illness and severe disease | 18-65 | mRNA-1273 | Clinical Trial, vaccine efficacy, local/systemic effects, wrong age-group |
| 34555004 | | Comparative Effectiveness of Moderna, Pfizer-BioNTech, and Janssen (Johnson & Johnson) Vaccines in Preventing COVID-19 Hospitalizations Among Adults Without Immunocompromising Conditions - United States, March-August 2021 | Self WH | Study comparing mRNA-1273, BNT and Janssen effectiveness against covid hospitalization in adults without immunocompromised conditions | ≥18 | BNT162b2, mRNA-1273, Ad26.COV2.S | Vaccine efficacy, Wrong age-group |
| 34735425 | | Laboratory-Confirmed COVID-19 Among Adults Hospitalized with COVID-19-Like Illness with Infection-Induced or mRNA Vaccine-Induced SARS-CoV-2 Immunity - Nine States, January-September 2021 | Bozio CH | Study investigating hospitalizations in adults with covid-19 like illness comparing odds of laboratory-confirmed covid between unvaccinated with previous infections vs fully vaccinated but no previous infection | ≥18 | BNT162b2, mRNA-1273 | Vaccine efficacy, wrong age-group |
| 34734975 | | Association Between mRNA Vaccination and COVID-19 Hospitalization and Disease Severity | Tenforde MW | Case-control study on mRNA vaccines, and their association with covid hospitalization, mechanical ventilation and death | ≥18, median 59, IQR 45-69 | BNT162b2, mRNA-1273 | Vaccine efficacy, wrong age-group |
| 34974072 | | Pfizer-BioNTech vaccine effectiveness against Sars-Cov-2 infection: Findings from a large observational study in Israel | Saciuk Y | Retrospective cohort study of vaccine effectiveness | 16+ | BNT162b2 | Vaccine efficacy |
| 35012777 | | Safety and effectiveness of BNT162b2 mRNA Covid-19 vaccine in adolescents | June Choe Y | Study on VE and safety of BNT162b2 in adolescents, but only shows reported rate | 16-18 | BNT162b2 | No comparator, vaccine efficacy |
| 35025852 | | Effectiveness of BNT162b2 (Pfizer-BioNTech) mRNA Vaccination Against Multisystem Inflammatory Syndrome in Children Among Persons Aged 12-18 Years - United States, July-December 2021 | Zambrano LD | Test-negative case control study on vaccine effectiveness of BNT162b2 vs MIS-C in 12-18-year-olds | 12-18 | BNT162b2 | Vaccine efficacy |
| 35239634 | | Effectiveness of COVID-19 Pfizer-BioNTech BNT162b2 mRNA Vaccination in Preventing COVID-19-Associated Emergency Department and Urgent Care Encounters and Hospitalizations Among Nonimmunocompromised Children and Adolescents Aged 5-17 Years - VISION Network, 10 States, April 2021-January 2022 | Klein NP | Effectiveness study on BNT162b2 vs. covid-associated emergency department and urgent care encounters, and hospitalizations | 5-17 | BNT162b2 | Vaccine efficacy |
| 35298453 | | Effectiveness of 2-Dose BNT162b2 (Pfizer BioNTech) mRNA Vaccine in Preventing SARS-CoV-2 Infection Among Children Aged 5-11 Years and Adolescents Aged 12-15 Years - PROTECT Cohort, July 2021-February 2022 | Fowlkes AL | Study on effectiveness of 2-dose BNT vs infection in 5-11- & 12–15-year-olds | 5-15 | BNT162b2 | Vaccine efficacy |
| 35410884 | | Risk of adverse events after covid-19 in Danish children and adolescents and effectiveness of BNT162b2 in adolescents: cohort study | Kildegaard H | Cohort study of adverse events after SARS-CoV-2 infection in children and adolescents and effectiveness of BNT162b2 | <18 | BNT162b2 | Vaccine efficacy, Covid cohort |
| 35974428 | | Effectiveness of Coronavirus Disease 2019 Vaccines Against Hospitalization and Death in Canada: A Multiprovincial, Test-Negative Design Study | Nasreen S | Study on vaccine effectiveness against hospitalizations and death | ≥18 | BNT162b2, mRNA-1273, ChAdOx1 | Vaccine efficacy |
| 35976000 | | Loss of Pfizer (BNT162b2) Vaccine-Induced Antibody Responses against the SARS-CoV-2 Omicron Variant in Adolescents and Adults | Gupta SL | Study on weak antibody response in adolescents and adults to omicron (efficacy study) | 11-16, 27-55 | BNT162b2 | Clinical characteristics, vaccine efficacy |
| 36264830 | | Effectiveness of Monovalent mRNA Vaccines Against COVID-19-Associated Hospitalization Among Immunocompetent Adults During BA.1/BA.2 and BA.4/BA.5 Predominant Periods of SARS-CoV-2 Omicron Variant in the United States - IVY Network, 18 States, December 26, 2021-August 31, 2022 | Surie D | Case control study on effectiveness of monovalent mRNA vaccines vs. COVID-19 associated hospitalization among immunocompetent adults during Omicron in US | ≥18 | mRNA | Vaccine efficacy, wrong age-group, specific group |
| 36415904 | | Protection of Two and Three mRNA Vaccine Doses Against Severe Outcomes Among Adults Hospitalized With COVID-19-VISION Network, August 2021 to March 2022 | DeSilva MB | Study assessing protection of 2/3 mRNA vaccine doses against severe outcomes in adults hospitalized with covid | ≥18 | COVID-19 mRNA vaccines | Vaccine effectiveness, wrong age-group |
| 37104244 | | Effectiveness of Monovalent mRNA COVID-19 Vaccination in Preventing COVID-19-Associated Invasive Mechanical Ventilation and Death Among Immunocompetent Adults During the Omicron Variant Period - IVY Network, 19 U.S. States, February 1, 2022-January 31, 2023 | DeCuir J | Case control study on effectiveness of mRNA vaccines in preventing mechanical ventilation and death among immunocompromised adults | ≥18 | mRNA | Vaccine effectiveness, specific group |
| 37227984 | | Estimates of Bivalent mRNA Vaccine Durability in Preventing COVID-19-Associated Hospitalization and Critical Illness Among Adults with and Without Immunocompromising Conditions - VISION Network, September 2022-April 2023 | Link-Gelles R | Study on vaccine effectiveness of bivalent booster in adults with and without immunocompromising conditions | ≥18 | bivalent mRNA booster | Specific group, vaccine effectiveness, wrong age-group |
| 37877845 | | COVID-19 vaccine effectiveness in children by age groups. A population-based study in Galicia, Spain | Mallah N | Population-based test-negative study on vaccine effectiveness in children and adolescents | 5-17 | BNT162b2, mRNA-1273 | Vaccine effectiveness |
| 38635481 | | Durability of Original Monovalent mRNA Vaccine Effectiveness Against COVID-19 Omicron-Associated Hospitalization in Children and Adolescents - United States, 2021-2023 | Zambrano LD | Case control study on vaccine effectiveness of monovalent BNT162b2 mRNA vaccine on omicron hospitalization | 5-18 | mRNA | Vaccine effectiveness |
| 39552125 | | Sex differences in response to COVID-19 mRNA vaccines in Italian population | Ferroni E | Italian nationwide retrospective cohort study of vaccine effectiveness by sex | 12+ | mRNA | Vaccine effectiveness |
|  | **Descriptive (demographic or clinical characteristics) studies (n=74)** |  |  |  |  |  |  |
| 34044428 | | BNT162b2 vaccine induces neutralizing antibodies and poly-specific T cells in humans | Sahin U | Clinical trial of BNT162b2 on antibodies and T cell response | 19-55 | BNT162b2 | Clinical Trial, Clinical characterisitcs, wrong age-group, local/systemic reactions |
| 34241676 | | Poor humoral and T-cell response to two-dose SARS-CoV-2 messenger RNA vaccine BNT162b2 in cardiothoracic transplant recipients | Schramm R | Study on humoral and T-cell response to 2-dose BNT vaccination in cardiothoracic transplant recipients | 18-60+ | BNT162b2 | Clinical characteristics, specific group, wrong age-group |
| 34282971 | | Myocarditis Associated with mRNA COVID-19 Vaccination | Starekova J | Study describing cardiac MRI findings in patients with myocarditis detected shortly after covid mRNA vaccination | 17-38 | BNT162b2, mRNA-1273 | Wrong age-group, clinical characteristics, case series |
| 34339728 | | Myopericarditis after messenger RNA Coronavirus Disease 2019 Vaccination in Adolescents 12 to 18 Years of Age | Das BB | Study characterizing clinical course and outcomes of children who developed myocarditis after BNT162b2 vaccination | 12-18 | BNT162b2 | Clinical characteristics, case series |
| 34381004 | | Serological Response to the BNT162b2 COVID-19 mRNA Vaccine in Adolescent and Young Adult Kidney Transplant Recipients | Haskin O | Study on serological response to BNT162b2 in adolescents and young adults Kidney transplant recipients | 13.5-26.8 | BNT162b2 | Clinical characteristics, specific group, local/systemic reactions, no comparator |
| 34389692 | | COVID-19 Vaccination-Associated Myocarditis in Adolescents | Jain SS | Study characterizing clinical presentation, prognosis and myocardial tissue change in patients with covid vaccination associated myocarditis | <21 | mRNA COVID-19 vaccination | Clinical characteristics |
| 34402230 | | Epidemiology and Clinical Features of Myocarditis/Pericarditis before the Introduction of mRNA COVID-19 Vaccine in Korean Children: a Multicenter Study | Park H | Study describing frequency, clinical characteristics, etiology and outcome of myo/pericarditis in children between 2010-2019 | 11 days - 17.8 | - | Clinical characteristics, no comparator |
| 34492260 | | Antibody responses to the SARS-CoV-2 vaccine in individuals with various inborn errors of immunity | Delmonte OM | Study on safety and antibody responses to covid vaccine in individuals with inborn error of immunity | 16-71 | BNT162b2, mRNA-1273, Ad26.COV2.S | Clinical characteristics, specific group, local/systemic reactions |
| 34614328 | | Myocarditis after BNT162b2 mRNA Vaccine against Covid-19 in Israel | Mevorach D | Study retrospectively reviewing data on myocarditis, comparing incidence after vaccination with expected | 16-19, 20-24, 25-29, 30-39, 40-49, 50+ | BNT162b2 | Clinical characteristics, historical comparator |
| 34614329 | | Myocarditis after Covid-19 Vaccination in a Large Health Care Organization | Witberg G | Study searching for myocarditis in patients recently receiving BNT162b2, estimate incidence and clinical characteristics | 21-35 | BNT162b2 | Wrong age-group, Clinical characteristics, case report/series, no comparator |
| 34645698 | | COVID-19 Vaccine Type and Humoral Immune Response in Patients Receiving Dialysis | Garcia P | Study on humoral immune response after covid vaccination in patients receiving dialysis | 18-80+ | BNT162b2, mRNA1273, Ad26.COV2.S | Clinical characteristics, wrong age-group, specific group |
| 34671350 | | Humoral and Cellular Response Following Vaccination With the BNT162b2 mRNA COVID-19 Vaccine in Patients Affected by Primary Immunodeficiencies | Amodio D | Study on humoral and cellular response after BNT162b2 vaccination in patients with primary immunodeficinecy | 16-59 | BNT162b2 | Clinical characteristics, wrong age-group, specific group, local/systemic reactions |
| 34704459 | | Cardiac MRI Findings of Myocarditis After COVID-19 mRNA Vaccination in Adolescents | Chelala L | Study on MRI findings of myocarditis after mRNA vaccination in adolescents | 16-19 | BNT162b2, mRNA-1273 | Clinical characteristics, case series |
| 34844930 | | Immunogenicity of the COVID-19 mRNA vaccine in adolescents with juvenile idiopathic arthritis on treatment with TNF inhibitors | Dimopoulou D | Letter on humoral response/immunogenicity of covid mRNA vaccine in adolescents with JIA treated with TNF inhibitors | 16-21 | BNT162b2 | Clinical characteristics, specific group |
| 34860360 | | Features of Inflammatory Heart Reactions Following mRNA COVID-19 Vaccination at a Global Level | Chouchana L | Vigibase study on demographic features of inflammatory heart reactions following mRNA vaccination, comparing older vs younger and sexes | 12-17 and 18-29, compared to 30+ | BNT162b2, mRNA-1273 | Between-group comparison, clinical characteristics |
| 34865500 | | Clinically Suspected Myocarditis Temporally Related to COVID-19 Vaccination in Adolescents and Young Adults: Suspected Myocarditis After COVID-19 Vaccination | Truong DT | case series of 139 adolescents/young adults with suspected myocarditis, describing MRI, ECG and laboratory data | 12-20 | BNT162b2, mRNA-1273, Ad26.COV2.S | Case report/series, clinical characteristics |
| 34889875 | | Population-based Incidence of Myopericarditis After COVID-19 Vaccination in Danish Adolescents | Nygaard U | Prospective multicenter study identifying myopericarditis after BNT162b2 vaccination in Danish adolescents | 12-17 | BNT162b2 | No comparator, clinical characteristics, case report/series |
| 35076665 | | Myocarditis Cases Reported After mRNA-Based COVID-19 Vaccination in the US From December 2020 to August 2021 | Oster ME | Descriptive study of reports of myo/pericarditis to VAERS | 12+ (median 21, IQR 16-31) | BNT162b2, mRNA-1273 | Clinical characteristics |
| 35123676 | | COVID-19 vaccine-induced antibody responses in immunosuppressed patients with inflammatory bowel disease (VIP): a multicentre, prospective, case-control study | Alexander JL | Study on antibody responses induced by covid vaccination in immunosuppressed patients with IBD | >18 | BNT162b2, mRNA-1273, ChAdOx1 | Wrong age-group, clinical characteristics, specific group |
| 35166587 | | Myocardial Injury Pattern at MRI in COVID-19 Vaccine-Associated Myocarditis | Fronza M | Study using MRI describing myocardial injury pattern in myocarditis after covid vaccination | mean age 31 | BNT162b2, mRNA-1273 | Clinical characteristics, between-group comparison |
| 35199166 | | Robust neutralizing antibody response to SARS-CoV-2 mRNA vaccination in adolescents and young adults with childhood-onset rheumatic diseases | Yeo JG | Study investigating neutralizing antibodies (immunogenicity) in adolescents and young adults with childhood-onset rheumatic diseases | median 16.9, IQR 14.7-19.5 | BNT162b2, mRNA-1273 | Clinical characteristics, specific group |
| 35224605 | | Pericarditis and myocarditis after COVID-19 mRNA vaccination in a nationwide setting | Yap J | Study describing the incidence of peri/myocarditis by age and sex after mRNA vaccination, compared to background incidence | 12-59 | BNT162b2 | Historical comparator, descriptive, case report/series |
| 35320390 | | CMR Imaging 6 Months After Myocarditis Associated with the BNT162b2 mRNA COVID-19 Vaccine | Amir G | Study on early and 6-month follow-up of patients with myocarditis after BNT162b2 vaccination | 15-19 | BNT162b2 | Clinical characteristics |
| 35351530 | | Persistent Cardiac Magnetic Resonance Imaging Findings in a Cohort of Adolescents with Post-Coronavirus Disease 2019 mRNA Vaccine Myopericarditis | Schauer J | Study describing the evolution of cardiac magnetic resonance imaging of 16 adolescents with myopericarditis after 2nd dose BNT | 12-17 | BNT162b2 | Clinical characteristics |
| 35359754 | | Extension and Severity of Self-Reported Side Effects of Seven COVID-19 Vaccines in Mexican Population | Camacho Moll ME | Study comparing self-reported side effects from 7 different vaccines | 18+ | BNT, mRNA-1273, Ad26.COV2.S, sputnik, ChAdOx, CoronaVac, Ad5-ncov | Wrong age-group, between-group comparison, no comparator, clinical characteristics, local/systemic reactions |
| 35472554 | | Immune Responses to SARS-CoV-2 Vaccination in Young Patients with Anti-CD19 Chimeric Antigen Receptor T Cell-Induced B Cell Aplasia | Jarisch A | Study on immune responses to covid vaccination in young patients with anti-CD19 CAR T-cell induced B cell aplasia | >12 | mRNA COVID-19 vaccines | Clinical characteristics |
| 35482094 | | Follow-up cardiac magnetic resonance in children with vaccine-associated myocarditis | Hadley SM | Study on follow-up data on cardiac magnetic resonance in children with vaccine-associated myocarditis | 12-18 | BNT162b2 | Clinical characteristics |
| 35510786 | | Antibody response to three SARS-CoV-2 mRNA vaccines in adolescent solid organ transplant recipients | Qin CX | Study on antibody response to mRNA vaccines in adolescent solid organ transplant recipients | 12-18 | BNT162b2, mRNA-1273 | Clinical characteristics, specific group |
| 35720281 | | mRNA or ChAd0x1 COVID-19 Vaccination of Adolescents Induces Robust Antibody and Cellular Responses With Continued Recognition of Omicron Following mRNA-1273 | Dowell AC | Study on antibody and cellular responses in adolescents with severe neuro-disabilities who were vaccinated with ChAdOx or mRNA vaccines | 12-16 | BNT162b2, mRNA-1273, ChAdOx1 | Clinical characteristics, specific group |
| 35738253 | | Perinatally Human Immunodeficiency Virus-Infected Adolescents and Young Adults Demonstrate Distinct BNT162b2 Messenger RNA Coronavirus Disease 2019 Vaccine Immunogenicity | Morrocchi E | Study on immunogenicity of COVID-19 mRNA vaccine in perinatally HIV-infected adolescents and young adults | HIV+ 18-35, healthy controls 24-59 | BNT162b2 | Clinical characteristics, specific group |
| 35759003 | | Antibody response to 2- and 3-dose SARS-CoV-2 mRNA vaccination in pediatric and adolescent kidney transplant recipients | Crane C | Study on antibody responses in pediatric/adolescent kidney transplant recipients | median 18, IQR 15-20 | BNT162b2, mRNA-1273 | Clinical characteristics, specific group |
| 35901198 | | Longitudinal Immune Response to 3 Doses of Messenger RNA Vaccine Against Coronavirus Disease 2019 (COVID-19) in Pediatric Patients Receiving Chemotherapy for Cancer | Lehrnbecher T | Study on immune response after 3 doses mRNA in cancer patients | 13-18 | BNT162b2 | Clinical characteristics, specific group |
| 35944800 | | Natural History of Myocardial Injury After COVID-19 Vaccine-Associated Myocarditis | Mustafa Alhussein M | Study describing natural history of myocarditis after mRNA vaccination | 18-39 | mRNA COVID-19 vaccine | Clinical characteristics, wrong age-group |
| 35992746 | | SARS-CoV2 mRNA Vaccine-Specific B-, T- and Humoral Responses in Adolescents After Kidney Transplantation | Sattler A | Study on covid mRNA vaccine-specific B, T and humoral responses in adolescents after kidney transplantation | 12.5-17.5 | BNT162b2 | Specific group, clinical characteristics |
| 36006288 | | Cardiovascular Manifestation of the BNT162b2 mRNA COVID-19 Vaccine in Adolescents | Mansanguan S | Study investigating cardiovascular manifestation after covid vaccination describing demographics and characteristics (symptoms, vital signs, ECG, cardic biomarkers, lab values). | 13-18 | BNT162b2 | Clinical characteristics |
| 36006062 | | Myocarditis Following COVID-19 Vaccination: Cardiac Imaging Findings in 118 Studies | Keshavarz P | Review of cardiac imaging findings of myocarditis after covid vaccination | 12-80 | BNT162b2, mRNA-1273, ChAdOx1, Ad26.COV2.S, COVAXIN, unknown mRNA | Review, clinical characteristics |
| 36053607 | | Myocarditis and myopericarditis cases following COVID-19 mRNA vaccines administered to 12-17-year olds in Victoria, Australia | Cheng DR | Description and clinical review of SAFEVIC reports of myo/pericarditis in 12–17-year-olds | 12-17 | mRNA COVID-19 vaccine | Clinical characteristics, between-group comparison |
| 36152650 | | Outcomes at least 90 days since onset of myocarditis after mRNA COVID-19 vaccination in adolescents and young adults in the USA: a follow-up surveillance study | Kracalik I | Study on outcomes up to 90 days since onset of myocarditis after vaccination in adolescents | 12-29 | mRNA COVID-19 vaccination | Clinical characteristics, Wrong age-group |
| 36298483 | | Impact of Anti-TNFα Treatment on the Humoral Response to the BNT162b2 mRNA COVID-19 Vaccine in Pediatric Inflammatory Bowel Disease Patients | Kashiwagi K | Study investigating the impact of anti-TNFalpha treatment on humoral response to BNT162b2 in pediatric IBD patients | <18 | BNT162b2 | Clinical characteristics, specific group |
| 36310037 | | Immunogenicity of SARS-CoV-2 vaccination in adolescents with cardiac disease | Hayashi H | Study of immunogenicity (anti‐spike antibodies, neutralizing activities, and interferon‐gamma production) in adolescents with cardiac disease |  |  | Specific group, clinical characteristics |
| 36327162 | | Safety Monitoring of Bivalent COVID-19 mRNA Vaccine Booster Doses Among Persons Aged ≥12 Years - United States, August 31-October 23, 2022 | Hause AM | Study describing spontaneous reports from VAERS and v-safe after bivalent booster mRNA vaccination | 12-101 | BNT162b2, mRNA-1273 | Clinical characteristics, no comparison |
| 36576105 | | Admission and follow-up cardiac magnetic resonance imaging findings in BNT162b2 Vaccine-Related myocarditis in adolescents | Özen S | Study on myocarditis (characteristics and follow-up) in adolescents with BNT162b2-vaccine related myocarditis. MRI | 14-17 | BNT162b2 | Clinical characteristics |
| 36597886 | | Circulating Spike Protein Detected in Post-COVID-19 mRNA Vaccine Myocarditis | Yonker LM | Immunoprofiling study of individuals who developed postvaccine myocarditis vs control subjects | 12-21 | BNT162b2, mRNA-1273 | Clinical characteristics |
| 36638405 | | Higher Troponin Levels on Admission are associated With Persistent Cardiac Magnetic Resonance Lesions in Children Developing Myocarditis After mRNA-Based COVID-19 Vaccination | Manno EC | Study investigating clinical data, laboratory values at admission, discharge and follow-up of patients admitted to the emergency department with myo/peri after mRNA vaccination | 12-17 | BNT162b2, mRNA-1273 | Clinical characteristics |
| 36650738 | | Subclinical myocardial assessment after BNT162b2 messenger RNA COVID-19 vaccination in adolescents with chronic heart disease: a speckle-tracking echocardiography study | Başkan S | Echocardiography study on Myocarditis | 12-18 | mRNA | Clinical characteristics |
| 36804307 | | Risk of myocarditis and pericarditis following coronavirus disease 2019 messenger RNA Vaccination-A nationwide study | Su WJ | Nationwide study on myo/pericarditis using spontaneous reports from Taiwan vaccine adverse events reporting system | 12-17, 18-24, ...,70,79, 80+ | BNT162b2, mRNA-1273 | Clinical characteristics, between-group comparison |
| 36825020 | | Immunogenicity of SARS-CoV-2 mRNA intramuscular vaccination in patients with muscular disorders | Kasai R | Study on the immunogenicity of mRNA intramuscular vaccination in patients with muscular disorders | 12-64 | BNT162b2 | Clinical characteristics, specific group, wrong age-group |
| 36841638 | | Acute Myocarditis and Pericarditis After mRNA COVID-19 Vaccinations-A Single-Centre Retrospective Analysis | Wassif M | Retrospective review of myo/pericarditis cases at a single hospital, and a case series of the ten cases found after COVID-19 mRNA vaccination | mean age 33+-9 | BNT162b2 | Clinical characteristics, case report/series, wrong age-group |
| 36841835 | | Incidence determinants and serological correlates of reactive symptoms following SARS-CoV-2 vaccination | Holt H | Study investigating associations between reactive symptoms and serological responses to vaccination | 16-70+ | BNT162b2, mRNA-1273, ChAdOx1 | Clinical characteristics, wrong age-group, local/systemic reactions |
| 37112661 | | Myocarditis and Pericarditis following COVID-19 Vaccination in Thailand | Mahasing C | Study investigating characteristics of myo/pericarditis and factors associated with myo/peri after vaccination in Thailand. historical controls | 5-11, 12-17, 18-20, 21-10, 41-60, 61-80, 80+ | ChAdOx, BNT, BBIBP, CoronaVac, mRNA-1273 | Between-group comparison, no comparator, clinical characteristics |
| 37264895 | | COVID-19 vaccination-related myocarditis: a Korean nationwide study | Cho JY | Nationwide cohort study reporting incidence men vs women, mRNA vs other vaccines, clinical outcomes after vaccine-related myocarditis | 12-17, 12-70+ | BNT162b2, mRNA-1273, ChAdOx1, Ad26.COV2.S | Clinical characteristics, between-group comparison, specific disease |
| 37271705 | | Medical outcomes of children with neurodevelopmental disorders after SARS-CoV-2 vaccination: A six-month follow-up study | Wang LJ | Study on medical outcomes of children with neurodevelopmental disorders after covid vaccination. | ≤12 | BNT162b2, mRNA-1273 | Clinical characteristics, specific group |
| 37294336 | | Longitudinal Assessment of Left Ventricular Function in Patients with Myopericarditis After mRNA COVID-19 Vaccination | Nv B | Longitudinal assessment of left ventricular function after vaccine myopericarditis | median 16 | BNT162b2, mRNA-1273 | Clinical characteristics, specific group |
| 37295197 | | Incidence and clinical characteristics of adverse neurological events and stroke-like syndrome associated with immune stress-related response after COVID-19 vaccination in 2021 from Thailand | Sirisuk W | Study using spontaneous reports on Incidence and clinical characteristics of adverse neurological events and stroke-like syndrome after adult COVID-19 vaccination. | >18 | CoronaVac, ChAdOX1, BNT162b2 | Wrong age-group, clinical characteristics |
| 37302919 | | BNT162b2 immunization-related myocarditis in adolescents and consequent hospitalization: Report from a medical center | Yen CW | Report on BNT-related myocarditis and hospitalization in adolescents | 12-18 | BNT162b2 | Clinical characteristics, no comparator/between-group comparison |
| 37616171 | | Imaging Acute and Chronic Cardiac Complications of COVID-19 and after COVID-19 Vaccination | Sánchez Tijmes F | Study on imaging findings in patients with vaccine-related myocarditis | - | mRNA COVID-19 vaccination | Clinical characteristics |
| 37872323 | | Emergency department presentations for chest complaints after mRNA COVID-19 vaccinations in children and adolescents | Parr M | Study on emergency department presentations for chest complaints | <18 | BNT162b2, mRNA-1273 | Clinical characteristics |
| 37882061 | | Echocardiographic function evaluation in adolescents following BNT162b2 Pfizer-BioNTech mRNA vaccination: A preliminary prospective study | Hsu WF | Study of serial echocardiographic examinations pre/post BNT162b2 vaccination | 12-15 | BNT162b2 | Clinical characteristics |
| 38100626 | | Autoantibodies against angiotensin-converting enzyme 2 (ACE2) after COVID-19 infection or vaccination | Tsoi JYH | Study measuring anti-ACE2 autoantibodies following covid infection, vaccination or in those with post-mRNA vaccine myopericarditis | median ages 50-62, myocarditis group mean age 14 | CoronaVac, BNT162b2 | Clinical characteristics |
| 38154991 | | Long term follow up and outcomes of Covid-19 vaccine associated myocarditis in Victoria, Australia: A clinical surveillance study | Shenton P | Study on long term follow-up and outcomes of vaccine-associated myocarditis using questionnaires | 10-90 | BNT162b2, mRNA-1273, ChAdOx1 | Clinical characterisitcs, wrong age-group, specific disease |
| 38365084 | | Assessing the temporal and cause-effect relationship between myocarditis and mRNA COVID-19 vaccines. A retrospective observational study | Bianchi FP | Retrospective study assessing causality of the myocarditis-mRNA vaccination association | 5-17, 18-39, 40-64, 65+; myocarditis <40 | BNT162b2, mRNA-1273 | clinical characteristics, between-group comparison, wrong age-group, single disease |
| 38521068 | | The central role of natural killer cells in mediating acute myocarditis after mRNA COVID-19 vaccination | Tsang HW | Study on NK cells in mediating myocarditis following mRNA vaccination | 12-17 | BNT162b2 | Clinical characteristics |
| 38910092 | | Revaccination outcomes among adolescents and adults with suspected hypersensitivity reactions following COVID-19 vaccination: A Canadian immunization research network study | Fitzpatrick T | Study on revaccination outcomes in individuals referred for suspected or diagnosed hypersensitivity | 12-65+ (median 31.5) | BNT162b2, mRNA-1273, ChAdOx1 | specific group, Clinical characteristics |
| 38940313 | | The effect of COVID-19 vaccination on multiple sclerosis activity as reflected by MRI | Ganelin-Cohen E | Study on safety of theBNT162b2 mRNA vaccine in multiple sclerosis (MS) patients measured by MRI | mean 16.4 and 34.9 (early/late MS onset group, respectively) | BNT162b2 | specific group, clinical characteristics |
| 39003104 | | Short- and long-term outcomes of cardiac adverse events following COVID-19 immunization managed in a Canadian pediatric center | Pham-Huy A | Single center retrospective case series of short and long term outcomes after a cardiac AEFI | 12-17 | BNT162b2 | Clinical characteristics, case series |
| 39093271 | | COVID-19 Vaccination-Related Pericarditis: A Korean Nationwide Study | Lee N | Nationwide report of confirmed cases of covid-19 vaccine related pericarditis by expert adjudication committee | All | BNT162b2, mRNA-1273, ChAdOx1, Ad26.COV2.S | Clinical characteristics, specific disease, no comparator |
| 39232036 | | Influence of mRNA Covid-19 vaccine dosing interval on the risk of myocarditis | Le Vu S | Matched case-control study assessing dosing interval and risk of myocarditis | 12+ | BNT162b2, mRNA-1273 | Clinical characteristics, between-group comparison |
| 39326653 | | Antibody response to SARS-CoV-2 mRNA vaccination in Danish adults exposed to perfluoroalkyl substances (PFASs): The ENFORCE study | Timmermann A | Study investigating if serum PFAS associates with antibody response after COVID-19 vaccination | 50-69 | BNT162b2 | Immune response, wrong age-group |
| 39420039 | | Nationwide Cohort observational study on the safety and efficacy of COVID-19 vaccination in patients with Moyamoya disease | Byoun HS | Study on incidence of stroke and mortality in patients with Moyamoya disease, with COVID-19 infection and vaccination as risk factors, also brief vaccine efficacy analysis. | 20-60+ | ChAdOX1, BNT162b2, mRNA-1273, Ad26.COV2.S, Novavax | Wrong age-group, specific disease, between-group comparison, clinical characteristics, specific group |
| 39489283 | | Healthcare utilization among COVID-19 mRNA vaccine-associated myocarditis cases: a matched retrospective cohort study | Naveed Z | Study evaluating all-cause healthcare utilization among COVID-19 mRNA vaccine-associated myocarditis cases | 12+ | BNT162b2, mRNA-1273 | Clinical characteristics, Wrong age-group, between-group comparison |
| 39625796 | | [Adverse events from the Pfizer-BioNTech® COVID-19vaccine in children 5-17 years old] | Hernández Morales MDR | Study describing spontaneous reports of adverse events possibly attributed to BNT162b2 vaccination | 5-17 | BNT162b2 | No comparator, clinical characteristics, local symptoms/reactions |
| 39655683 | | Clinical phenotype of COVID-19 vaccine-associated myocarditis in Victoria, 2021-22: a cross-sectional study | Smith J | Study describing the clinical phenotype of COVID-19 vaccine associated myocarditis | 10-76 | BNT162b2, mRNA-1273, ChAdOx1 | Clinical characteristics, wrong age-group, specific disease |
| 39716863 | | Epidemiological Characteristics and Outcome of Myocarditis and Pericarditis Temporally Associated With BNT162b2 COVID-19 Vaccine in Adolescents: Korean National Surveillance | Ahn B | Retrospective cohort on epidemiological characteristics and outcome of vaccine-related myo/pericarditis | 12-19 | BNT162b2 | Clinical characteristics, No comparator |
| 39776066 | | SARS-CoV-2 Activated Peripheral Blood Mononuclear Cells (PBMCs) Do Not Provoke Adverse Effects in Trophoblast Spheroids | Ayuk HS | Investigation of SARS-COV-2 activated PBMCs | 22-44 | BNT162b2, mRNA-1273, ChAdOx1 | Clinical characteristics |
|  | **Studies with wrong age-groups (n=78)** |  |  |  |  |  |  |
| 33301246 | | Safety and Efficacy of the BNT162b2 mRNA Covid-19 Vaccine | Polack FP | Clinical trial of BNT162b2 in ages 16+ | 16-55, 55+ | BNT162b2 | Clinical trial, wrong age-group, local/systemic reactions |
| 33378609 | | Efficacy and Safety of the mRNA-1273 SARS-CoV-2 Vaccine | Baden LR | Clinical trial of mRNA-1273 | 18+ | mRNA-1273 | Clinical trial, wrong age-group, local/systemic reactions |
| 33444297 | | Allergic Reactions Including Anaphylaxis After Receipt of the First Dose of Pfizer-BioNTech COVID-19 Vaccine - United States, December 14-23, 2020 | CDC COVID-19 Response Team | Manuscript on spontaneous reports of allergic reactions including anaphylaxis after first dose BNT162b2 using VAERS data | 27-60 | BNT162b2 | Wrong age-group |
| 33707061 | | A preliminary report of a randomized controlled phase 2 trial of the safety and immunogenicity of mRNA-1273 SARS-CoV-2 vaccine | Chu L | Clinical trial on safety and immunogenicity of mRNA-1273 | 18+ | mRNA-1273 | wrong age-group, Clinical Trial, local/systemic reactions |
| 33888900 | | Safety and immunogenicity of the SARS-CoV-2 BNT162b1 mRNA vaccine in younger and older Chinese adults: a randomized, placebo-controlled, double-blind phase 1 study | Li J | Clinical trial on safety and immunogenicity of BNT162b2 in Chinese | 18-55 and 65-85 | BNT162b2 | Clinical Trial, wrong age-group, local/systemic reactions |
| 34093567 | | COVID-19 mRNA Vaccines Are Generally Safe in the Short Term: A Vaccine Vigilance Real-World Study Says | Chen G | Study reporting VAERS data on AEFIs after covid vaccination in December 2020 | <18-65+ | BNT162b2, mRNA-1273 | Wrong age-group, between-group comparison |
| 34127481 | | Immunogenicity and safety of the BNT162b2 mRNA COVID-19 vaccine in adult patients with autoimmune inflammatory rheumatic diseases and in the general population: a multicentre study | Furer V | Immunogenicity and safety of BNT162b2 in adult patients with autoimmune inflammatory diseases and general population | 18+ | BNT162b2 | Wrong age-group, specific group |
| 34225791 | | Safety of SARS-CoV-2 vaccines: a systematic review and meta-analysis of randomized controlled trials | Chen M | Systematic review and meta-analysis of safety data from Randomized Clinical Trials | ≤55, >55 | BBIBP-CorV, CoronaVac, BNT162b2, other | Review, wrong age-group |
| 34347278 | | Cumulative Adverse Event Reporting of Anaphylaxis After mRNA COVID-19 Vaccine (Pfizer-BioNTech) Injections in Japan: The First-Month Report | Iguchi T | Study reporting on spontaneous reports on anaphylaxis and anaphylactoid symptoms after the first month of covid vaccination in Japan | health care workers, age 22-56, controls 16+ | BNT162b2 | Wrong age-group, between-group comparison, historical comparator, specific disease |
| 34365034 | | A comprehensive analysis of the efficacy and safety of COVID-19 vaccines | Cai C | Comprehensive meta-analysis of published trials and VAERS data on COVID-19 vaccine efficacy and safety | 18+ | BNT162b2, mRNA-1273, ChAdOx1, Ad26.COV2.S, CoronaVac, NVX-CoV2373, Ad5-nCov, BBIBP-CorV, Gam-COVID-Vac | Review, wrong age-group |
| 34398511 | | Safety of administration of BNT162b2 mRNA (Pfizer-BioNTech) COVID-19 vaccine in youths and young adults with a history of acute lymphoblastic leukemia and allergy to PEG-asparaginase | Mark C | Study on safety of BNT162b2 in youths and young adults with history of acute lymphoblastic leukemia and PEG-asparaginase allergy | 12-29 | BNT162b2 | Specific group, wrong age-group |
| 34411532 | | Bell's palsy following vaccination with mRNA (BNT162b2) and inactivated (CoronaVac) SARS-CoV-2 vaccines: a case series and nested case-control study | Wan EYF | Case series and nested case-control study of bells palsy following BNT162b2 and CoronaVac vaccination | 16-110 (median 60) | BNT162b2, CoronaVAC | Single disease, wrong age-group |
| 34432976 | | Safety of the BNT162b2 mRNA Covid-19 Vaccine in a Nationwide Setting | Barda N | National cohort used for target trial emulation on BNT162b2 safety in 16+ | 16-39, 40+ | BNT162b2 | Wrong age-group |
| 34446426 | | Risk of thrombocytopenia and thromboembolism after covid-19 vaccination and SARS-CoV-2 positive testing: self-controlled case series study | Hippisley-Cox J | SCCS study on risk of thrombocytopenia and thromboembolism after SARS-CoV-2 infection and vaccination | 16+; <50 and >50 analysis | ChAdOx1, BNT 162b2 | Wrong age-group |
| 34477808 | | Surveillance for Adverse Events After COVID-19 mRNA Vaccination | Klein NP | Interim analysis of safety surveillance data from vaccine safety datalink | 12->75, 20% <18 but only report whole population | BNT162b2, mRNA-1273 | Wrong age-group |
| 34492394 | | Facial nerve palsy following the administration of COVID-19 mRNA vaccines: analysis of a self-reporting database | Sato K | Disproportionality analysis of Facial nerve palsy from VAERS - comparing BNT162b2 and mRNA-1273 to historic influenza vaccination | 37-70 | BNT162b2, mRNA-1273 | Wrong age-group, between-group comparison |
| 34525277 | | Safety and Efficacy of the BNT162b2 mRNA Covid-19 Vaccine through 6 Months | Thomas SJ | Clinical trial of BNT162b2 vaccine safety and efficacy | 16+ (and 12-15 but no adverse events analysis) | BNT162b2 | Clinical Trial, wrong age-group, local/systemic symptoms |
| 34605853 | | Acute Myocarditis Following COVID-19 mRNA Vaccination in Adults Aged 18 Years or Older | Simone A | Cohort study investigating incidence and clinical outcomes of myocarditis in adults following mRNA vaccination | 18+ | BNT162b2, mRNA-1273 | Wrong age-group |
| 34709227 | | [Acute myocarditis in a young adult two days after Pfizer vaccination] | Facetti S | Case report of a adult myopericarditis case after 2nd dose BNT162b2 vaccination | 20 | BNT162b2 | Case report/series, wrong age |
| 34718945 | | Immunogenicity and Safety of COVID-19 mRNA Vaccine in STAT1 GOF Patients | Bloomfield M | Study on safety and immunogenicity of BNT162b2 in STAT1 GOF patients | 18-50 | BNT162b2 | Specific group, wrong-age group |
| 34734240 | | Myocarditis Following Coronavirus Disease 2019 mRNA Vaccine: A Case Series and Incidence Rate Determination | Perez Y | A case series of myocarditis after covid mRNA vaccination | 22-17 | BNT162b2, mRNA-1273 | Case report/series, wrong age-group |
| 34738774 | | A snapshot global survey on side effects of COVID-19 vaccines among healthcare professionals and armed forces with a focus on headache | Perrotta A | Study using side effect survey focusing on headache on armed forces and health care personnel | 18+ | BNT162b2, ChAdOx1 | Wrong age-group, specific outcome, local/systemic reactions |
| 34757289 | | A prospective multicenter study assessing humoral immunogenicity and safety of the mRNA SARS-CoV-2 vaccines in Greek patients with systemic autoimmune and autoinflammatory rheumatic diseases | Tzioufas AG | Study on humoral immunogenicity and safety of mRNA vaccines in patients with systemic autoimmune and autoinflammatory rheumatic diseases | 16-91 | BNT162b2, mRNA-1273 | Wrong age-group, specific group |
| 34907393 | | Risks of myocarditis, pericarditis, and cardiac arrhythmias associated with COVID-19 vaccination or SARS-CoV-2 infection | Patone M | SCCS cohort study of myo/pericardits and arrythmias using both COVID-19 vaccination and infection as exposures | 16+; 16-29, 29-39, 40+ | BNT162b2, mRNA-1273, ChAdOx1 | Wrong age-group |
| 34916207 | | SARS-CoV-2 vaccination and myocarditis or myopericarditis: population based cohort study | Husby A | Cohort study on myopericarditis after vaccination | 12-39, 40-59, 60+ | BNT162b2, mRNA-1273, ChAdOx1, Ad26.COV2.S | Wrong age-group |
| 34930152 | | Reactivation of BCG vaccination scars after vaccination with mRNA-Covid-vaccines: two case reports | Mohamed L | Case reports of BCG vaccination scar reactivation after mRNA covid vaccination | 49-53 | BNT162b2, mRNA-1273 | Case report/series, specific disease, wrong age-group |
| 35038274 | | Case Series of Thrombosis With Thrombocytopenia Syndrome After COVID-19 Vaccination-United States, December 2020 to August 2021 | See I | Case series of thrombosis with thrombocytopenia syndrome after covid-19 vaccination | 18-65+ | BNT162b2, mRNA-1273, Ad26.COV2.S | Case report/series, wrong age-group |
| 35131133 | | Efficacy and safety of the BNT162b2 mRNA COVID-19 vaccine in participants with a history of cancer: subgroup analysis of a global phase 3 randomized clinical trial | Thomas SJ | Trial of BNT162b2 in participants with a history of cancer | mean 62 | BNT162b2 | Clinical Trial, specific group, wrong age-group, local/systemic reactions |
| 35186864 | | Frequency and Associations of Adverse Reactions of COVID-19 Vaccines Reported to Pharmacovigilance Systems in the European Union and the United States | Montano D | Study presenting overview and relative incidence of adverse reactions reported to pharmacovigilance systems | 18-64, 65+ | BNT162b2, mRNA-1273, ChAdOx1, Ad26.COV2.S | Wrong age-group, between-group comparison |
| 35282400 | | Cardiogenic shock temporally associated with COVID-19 vaccination after prior COVID-19 infection: A case report | Jean-Marie EM | Case report of MIS-A associated with BNT162b2 vaccination | 21 | BNT162b2 | Case report/series, wrong age-group |
| 35297971 | | Incidence of Cerebral Venous Thrombosis Following SARS-CoV-2 Infection vs mRNA SARS-CoV-2 Vaccination in Singapore | Tu TM | study comparing incidence rates and clinical characteristics of cerebral venous thrombosis after SARS-CoV-2 infection or mRNA vaccines | 0-102 (median 34) | BNT162b2, mRNA-1273 | Wrong age-group, between-group comparison |
| 35317093 | | Transient Myopericarditis Following Vaccination for COVID-19 | Gill J | Case report of transient myocarditis in a 44-year-old healthy male patient | 44 | mRNA-1273 | Case report/series, wrong age-group |
| 35449353 | | Myocarditis following COVID-19 vaccination in adolescents and adults: a cumulative experience of 2021 | Ilonze OJ | Review of myocarditis following covid-19 vaccination in adolescents and adults | mean age 27 | BNT162b2, mRNA-1273 | Review, wrong age-group |
| 35647664 | | 4BNT162b2 mRNA COVID-19 vaccine and semen: What do we know? | Olana S | Study on BNT162b2 vaccination and spermatozoa parameters | 18-45 | BNT162b2 | Wrong age-group, specific disease |
| 35655235 | | Acute cardiac side effects after COVID-19 mRNA vaccination: a case series | Freise NF | Case series on acute cardiac side effects after covid mRNa vaccination | 13-56 | BNT162b2, mRNA-1273 | Case report/series, wrong age-group |
| 35691322 | | Risk of myocarditis and pericarditis after the COVID-19 mRNA vaccination in the USA: a cohort study in claims databases | Wong HL | Cohort study comparing rates in claims databases of myocarditis in 18–64-year-olds after mRNA vaccination with historical rates | 18-64 | BNT162b2, mRNA-1273 | Wrong age-group |
| 35750537 | | Myocarditis and/or pericarditis risk after mRNA COVID-19 vaccination: A Canadian head to head comparison of BNT162b2 and mRNA-1273 vaccines | Abraham N | Study comparing myo/pericarditis risk after mRNA-1273 and BNT162b2 using reporting rates from surveillance system | 18-39 | BNT162b2, mRNA-1273 | Wrong age-group, between-group comparison, single disease |
| 35750980 | | A Cross-Sectional Study of Untoward Reactions Following Homologous and Heterologous COVID-19 Booster Immunizations in Recipients Seventeen Years of Age and Older | Tamburro M | Cross-sectional study on mRNA booster safety in 17+ using interviews | 17+ | BNT162b2, mRNA-1273 | wrong age-group, between-group comparison, local/systemic reactions |
| 35830976 | | Incidence, risk factors, natural history, and hypothesised mechanisms of myocarditis and pericarditis following covid-19 vaccination: living evidence syntheses and review | Pillay J | Living evidence syntheses and review on incidence, risk factors, mechanism and natural history of myo/pericarditis following COVID-19 vaccination | 0-40+ | BNT162b2, mRNA-1273 | Review, wrong age-group |
| 35943891 | | Seizures following COVID-19 vaccination in Mexico: A nationwide observational study | Núñez I | Nationwide retrospective descriptive study on incidence of seizures on vaccinees in Mexico | median 36 IQR 25-49 | BNT162b2, mRNA-1273, Ad5‐nCoV | Wrong age-group, between-group comparison, comparing with administered doses |
| 35976470 | | Anaphylaxis to SARS-CoV-2 Vaccines in the Setting of a Nationwide Passive Epidemiological Surveillance Program | Toledo-Salinas C | Mexican nationwide observational study using spontaneous adverse event reports of anaphylaxis, describing incidence and characteristics of adult patients | 18+ | BNT162b2, mRNA-1273, ChAdOx1 nCov-19, rAd26-rAd5, Ad5-nCov, CoronaVac, Ad26.COV2-S | Wrong age-group, specific disease, between-group comparison |
| 35983236 | | Acute Myopericarditis after the Second Dose of mRNA COVID-19 Vaccine Mimicking Acute Coronary Syndrome | Pantsios C | Case report of 50-year-old man with myocarditis after second dose mRNA vaccine | 50 | BNT162b2 | case report/series, wrong age-group |
| 36183231 | | Self-reported adverse events within the seven days following the Spikevax® (Moderna) vaccination | Guerra-Estévez D | Study using telephone survey to collect self-reported adverse events after mRNA-1273 | 18-76 | mRNA1273 | Wrong age-group, local systemic reactions |
| 36288813 | | Comparative risk of thrombosis with thrombocytopenia syndrome or thromboembolic events associated with different covid-19 vaccines: international network cohort study from five European countries and the US | Li X | Meta-analysis comparing risk of thrombosis with thrombocytopenia with different covid-19 vaccines (data from France, Germany, the Netherlands, Spain, UK, US) | 18+ | BNT162b2, mRNA-1273, ChAdOx1, Ad26.COV2.S | Between-group comparison, wrong age-group |
| 36357091 | | Comparative Risk of Myocarditis/Pericarditis Following Second Doses of BNT162b2 and mRNA-1273 Coronavirus Vaccines | Naveed Z | Study comparing the risk of myo/pericarditis following second dose of BNT162b2 and mRNA-1273 | 18+ | BNT162b2, mRNA-1273 | Wrong age--group, between-group comparison |
| 36575896 | | Skin manifestations following anti-COVID-19 vaccination: A multicentricstudy from Turkey | Oguz Topal I | Study asking patients at dermatology clinic of AEFIs with questionnaires | 18-91 | Sinovac, BNT162b2 | wrong age-group, specific group, specific outcome |
| 37070591 | | [Reactogenicity Study of mRNA Vaccines Against COVID-19] | Inglés Torruella J | Study on reactogenicity in health care personnel using questionnaires, analyzing differences between vaccines | 17-40, >40 | BNT162b2, mRNA-1273 | between-group comparison, wrong age-group |
| 37183320 | | Self-reported adverse events after 2 doses of COVID-19 vaccine in Korea | Kwon Y | Study on self-reported adverse events comparing between doses, homo/heterologous regime, sex and age group | 20-60+ | BNT162b2, mRNA-1273, ChAdOx1 | Wrong age-group, local/systemic reactions |
| 37272559 | | A Nationwide Survey of mRNA COVID-19 Vaccinee's Experiences on Adverse Events and Its Associated Factors | Yoon D | Study using web-based surveys to investigate self-reported AEFIs | 18-49 | BNT162b2, mRNA-1273 | Wrong age-group, grouped outcomes, local/systemic reaction, between-group comparisons |
| 37391313 | | Immediate adverse reactions following COVID-19 vaccination among 16-65-year-old Danish citizens | Torp Hansen K | Study on 20 self-reported immediate adverse reactions | 16-65, has 17-25 | BNT162b2, mRNA-1273, ChAdOx1 | Wrong age-group, local/systemic reactions |
| 37439770 | | Immunogenicity and reactogenicity of heterologous COVID-19 vaccination in pregnant women | Chayachinda C | Open-labeled non-inferiority trial evaluated immunogenicity and reactogenicity of heterologous and homologous COVID-19 vaccination schedules in pregnant Thai women | 18-45 | BNT162b2, ChAdOx1, CoronaVac | Clinical trial, specific group, wrong age-group |
| 37582470 | | Immunogenicity and Safety of Booster SARS-CoV-2 mRNA Vaccine Dose in Allogeneic Hematopoietic Stem Cell Transplantation Recipients | Mittal A | Study on immunogenicity and safety in allogeneic hematopoietic stem cell transplantation recipients | median 59.5 | BNT162b2, mRNA-1273 | Specific group, wrong age--group |
| 37788935 | | Risk of Guillain-Barré Syndrome Following COVID-19 Vaccines: A Nationwide Self-Controlled Case Series Study | Le Vu S | SCCS study investigating Guillain-Barrè Syndrome risk after COVID-19 vaccination | 12-49, 50+ | BNT162b2, mRNA-1273, ChAdOx1, Ad26.COV2.S | Wrong age-group, Single disease |
| 37891048 | | Adverse events of acute nephrotoxicity reported to EudraVigilance and VAERS after COVID-19 vaccination | Anastassopoulou C | EudraVigilance/VAERS reporting rates study on acute kidney injury and renal failure | 18-64, 65+ | BNT162b2, mRNA-1273, ChAdOx1, Ad26.COV2.S | Wrong age-group, no comparator |
| 37921538 | | Adverse events after first and second doses of COVID-19 vaccination in England: a national vaccine surveillance platform self-controlled case series study | Tsang RS | SCCS using data from national sentinel network, historical comparator | 16+ (mean 52 for BNT162b2) | BNT162b2, mRNA-1273, ChAdOx1 | Wrong age-group, grouped AESIs |
| 38016322 | | Interim results from a phase I randomized, placebo-controlled trial of novel SARS-CoV-2 beta variant receptor-binding domain recombinant protein and mRNA vaccines as a 4th dose booster | Nolan TM | Interim results from clinical trial of two booster vaccines in adults | 18-64 | protein-RBD, mRNA-RBD | Clinical Trial, wrong age-group |
| 38101473 | | Comparative safety analysis of mRNA and adenoviral vector COVID-19 vaccines: a nationwide cohort study using an emulated target trial approach | Choi MJ | Target trial emulation study using National Health Insurance Service database, comparing ChAdOx1 vs mRNA-1273/BNT162b2 in adults | 18-59, 60-74 | ChAdOx1, BNT162b2, mRNA-1273 | Wrong age-group, between-group comparison |
| 38319332 | | Neonatal Outcomes After COVID-19 Vaccination in Pregnancy | Norman M | Study on vaccination in Pregnancy | Mothers <25->40, and neonates | BNT162b2, mRNA-1273 | Wrong age-group |
| 38408705 | | The Risk of Optic Neuritis following mRNA Coronavirus Disease 2019 Vaccination Compared to Coronavirus Disease 2019 Infection and Other Vaccinations | Shukla P | Study on risk of optic neuritis after mRNA COVID-19 vaccination | mean age 45.5 | mRNA COVID-19 vaccines | Wrong age-group, specific disease |
| 38460680 | | A randomized double-blinded trial to assess recurrence of systemic allergic reactions following COVID-19 mRNA vaccination | Khalid MB | Trial on recurrence of systemic allergic reactions after mRNA vaccination | 16-69 | BNT162b2 | Clinical Trial, wrong age-group |
| 38552793 | | Intradermal delivery of the third dose of the mRNA-1273 SARS-CoV-2 vaccine: safety and immunogenicity of a fractional booster dose | Roozen GVT | Trial of mRNA-1273 Booster in COVID-19 naive adults | 18-30 | mRNA-1273 | Clinical Trial, wrong age-group |
| 38602888 | | Assessment of Risk for Sudden Cardiac Death Among Adolescents and Young Adults After Receipt of COVID-19 Vaccine - Oregon, June 2021-December 2022 | Liko J | Spontaneous report assessment of sudden cardiac death following covid-19 vaccine in adolescents and young adults | 16-30 | mRNA COVID-19 vaccines | Wrong age-group, no comparator, specific outcome |
| 38718486 | | Herpesviral Keratitis Following COVID-19 Vaccination: Analysis of NHIS Database in Korea | Lee TE | Nationwide cohort study using propensity score matching investigating risk of new-onset and relapse of Herpesviral keratitis following COVID-19 vaccination | <19->70 | mRNA and non-mRNA | Wrong age-group |
| 38757631 | | Association between mRNA COVID-19 vaccine boosters and mortality in Japan: The VENUS study | Mimura W | Nested case control of mortality after mRNA booster vaccination | 18-64, 65+ | BNT162b2, mRNA-1273 | Wrong age-group |
| 38907283 | | A propensity score approach and a partitioned approach for the self-controlled case series design to evaluate safety of a 2-dose vaccine series: application to myocarditis/pericarditis following mRNA COVID-19 vaccination | Xu S | Study proposing and testing propensity score SCCS and partitioned SCCS using simulated data, and analyzing myocarditis/pericarditis as a real-world example | 12-39 | BNT162b2, mRNA-1273 | Wrong age-group |
| 38944652 | | COVID-19 vaccination and major cardiovascular and haematological adverse events in Abu Dhabi: retrospective cohort study | Pimentel MAF | Study investigating myocarditis/pericarditis, non-haemorrhagic and haemorrhagic stroke, acute myocardial infarction, pulmonary embolism, venous thromboembolism, and disseminated intravascular coagulation after vaccination | 12-65 | BNT162b2, BBIBP-CorV | Wrong age-group, specific AEs |
| 38991137 | | COVID-19 mRNA vaccination responses in individuals with sickle cell disease: an ASH RC Sickle Cell Research Network Study | Anderson AR | Clinical trial of BNT162b2 vaccination reporting safety and immune responses in individuals with sickle cell disease | adult and pediatric participants | BNT162b2 | Clinical Trial, specific group, wrong age-group |
| 39039113 | | Long-term risk of autoimmune diseases after mRNA-based SARS-CoV2 vaccination in a Korean, nationwide, population-based cohort study | Jung SW | Nationwide cohort study investigating if development of autoimmune connective tissue diseases is associated with mRNA vaccination | <40->60 | BNT162b2, mRNA-1273 | Wrong age-group, historical comparator |
| 39052718 | | Immunogenicity and Safety of Heterologous Omicron BA.1 and Bivalent SARS-CoV-2 Recombinant Spike Protein Booster Vaccines: A Phase 3 Randomized Clinical Trial | Bennett C | Clinical trial reporting safety and immunogenicity of booster vaccines, comparing NVX-VOC2515 vs. NVX-COV2373 vs. bivalent mixture | 18-64 | NVX-CoV2515, NVX-CoV2373 | Clinical Trial, wrong age-group |
| 39068268 | | Acute Appendicitis After COVID-19 Vaccines in Italy: A Self-Controlled Case Series Study | Morciano C | SCCS study investigating acute appendicitis following COVID-19 vaccination using regional health databases | 12-39, 40-59, 60+ | BNT162b2, mRNA-1273, ChAdOx1, Ad26.COV2.S | Wrong age, specific disease |
| 39085208 | | Cohort study of cardiovascular safety of different COVID-19 vaccination doses among 46 million adults in England | Ip S | Cohort study of cardiovascular safety in adults | 18-90+ | BNT162b2, mRNA-1273, ChAdOx1 | Wrong age-group |
| 39103148 | | SARS-CoV-2 mRNA vaccine-related myocarditis and pericarditis: An analysis of the Japanese Adverse Drug Event Report database | Takada K | Study counting myocarditis and pericarditis adverse event reports after mRNA vaccination presenting reporting odds ratios | 12-71+ | BNT162b2, mRNA-1273 | Wrong age-group, no comparator |
| 39209823 | | Long-term safety and effectiveness of mRNA-1273 vaccine in adults: COVE trial open-label and booster phases | Baden LR | Clinical trial of mRNA-1273 long term safety and booster in adults | 18-64, 65+ | mRNA-1273 | Clinical Trial, wrong age-group |
| 39226788 | | Risk of heavy menstrual bleeding following COVID-19 vaccination: A nationwide case-control study | Botton J | Nationwide case control study on risk of heavy menstrual bleeding | 15-50 | BNT162b2, mRNA-1273 | Wrong age-group, specific disease |
| 39378869 | | Risk for Facial Palsy after COVID-19 Vaccination, South Korea, 2021-2022 | Yoon D | SCCS study investigating facial palsy following COVID-19 vaccination using national registers | 18+ | BNT162b2, mRNA-1273, ChAdOx1, Ad26.COV2.S | Wrong age-group, specific disease |
| 39413493 | | Participant-reported neurological events following immunization in the Canadian National Vaccine Safety Network-COVID-19 vaccine (CANVAS-COVID) study | Top KA | Study using online survey-based surveys to collect self-reported spontaneous reports of AEFIs | 11+ | BNT162b2, mRNA-1273, ChAdOx1 | Specific symptoms, wrong age-group |
| 39447252 | | Safety of mRNA COVID-19 vaccines among persons 15- years and above in Ghana: A cohort event monitoring study | Darko DM | A cohort event monitoring study in vaccination centers in Ghana | 15+ | BNT162b2, mRNA-1273 | Wrong age-group, local/systemic reactions, between-group comparison |
| 39550007 | | The Platform Trial In COVID-19 priming and BOOsting (PICOBOO): The immunogenicity, reactogenicity, and safety of licensed COVID-19 vaccinations administered as a second booster in BNT162b2 primed individuals aged 18-<50 and 50-<70 years old | McLeod C | Trial of second booster, comparing BNT162b2, mRNA-1273 and NVX-CoV2373 | 18-70 | BNT162b2 | Clinical trial, wrong age-group |
|  | | **Studies without comparable comparator (n=26)** |  |  |  |  |  |
| 34710075 | | COVID-19 Vaccination and Non-COVID-19 Mortality Risk - Seven Integrated Health Care Organizations, United States, December 14, 2020-July 31, 2021 | Xu S | Study using Vaccine Safety Datalink data investigating COVID-19 vaccination vs. non-COVID-19 mortality, comparing covid vaccinees with people vaccinated with influenza last two years | 12-17, 18-44, 4-64, 65-74, 75-84, 85+ | BNT162b2, mRNA-1273, Ad26.COV2.S | Single disease, Between-group comparison |
| 34849667 | | Myocarditis and pericarditis in adolescents after first and second doses of mRNA COVID-19 vaccines | Foltran D | Vigibase study reporting rates of myo/pericarditis in adolescents receiving mRNA vaccine comparing dose 2 vs 1, boys vs girls, BNT vs mRNA-1273 | 12-17 | BNT162b2, mRNA-1273 | Between-group comparison |
| 34921101 | | Age-Stratified Risk of Cerebral Venous Sinus Thrombosis After SARS-CoV-2 Vaccination | Krzywicka K | EudraVigilance database study on risk of cerebral venous sinus thrombosis with and without thrombocytopenia. Denominator is vaccine doses |  | ChAdOx1 nCov-19, Ad26.COV2.S, BNT162b2, mRNA-1273 | No comparator, specific disease |
| 35073155 | | Carditis After COVID-19 Vaccination With a Messenger RNA Vaccine and an Inactivated Virus Vaccine : A Case-Control Study | Lai FTT | Case control study of carditis after mRNA or Sinovac, comparing hospitalized for carditis vs hospitalized for other diagnoses | 12+; 12-17, 18+, mean age 57, only 143 <17 | BNT162b2, CoronaVac | Specific disease, between-group comparison |
| 35749115 | | Epidemiology of Myocarditis and Pericarditis Following mRNA Vaccination by Vaccine Product, Schedule, and Interdose Interval Among Adolescents and Adults in Ontario, Canada | Buchan SA | Population-based cohort study on myo/pericarditis using spontaneous reports, comparing vaccines, ages, intervals and schedules | 12-81 | BNT162b2, mRNA-1273 | Between-group comparison |
| 35924406 | | BNT162b2 mRNA Vaccination Against Coronavirus Disease 2019 is Associated With a Decreased Likelihood of Multisystem Inflammatory Syndrome in Children Aged 5-18 Years-United States, July 2021 - April 2022 | Zambrano LD | Case control study of hospitalized children comparing being fully vaccinated vs unvaccinated in MIS-C patients | 5-18 | BNT162b2 | Between-group comparison |
| 36152928 | | Safety of and antibody response to the BNT162b2 COVID-19 vaccine in adolescents and young adults with underlying disease | Shoji K | Study on safety and antibody response to BNT162b2 vaccine in adolescents and young adults with underlying disease | 12-25; 12-15, 16-25 | BNT162B2 | Specific group, between-group comparison, local/systemic reaction |
| 36277554 | | Evaluation of Short-Term Symptoms Associated With COVID-19 Vaccines Used Among Adolescents in Saudi Arabia | Alrowdhan FK | Retrospective cross-sectional study using self-reported questionnaires | 12-18 | BNT162b2, mRNA-1273 | Local/systemic reactions, no comparator |
| 36379531 | | Evaluate the side effect associated with COVID-19 vaccine on adolescents in Riyadh, Saudi Arabia: A cross-section study | Aldali JA | Cross-sectional study using questionnaires on symptoms and menstrual disorders following COVID-19 mRNA vaccination in adolescents | 12-17 | BNT162b2, mRNA-1273 | specific disease, between-group comparison, local/systemic reactions |
| 36848096 | | Myocarditis or Pericarditis Events After BNT162b2 Vaccination in Individuals Aged 12 to 17 Years in Ontario, Canada | Buchan SA | Study using a population-based cohort to investigate incidence of myocarditis and pericarditis after BNT162b2 vaccination | 12-17 | BNT162b2 | specific disease, between-group comparison, historical comparator |
| 36872145 | | Anaphylaxis rates following mRNA COVID-19 vaccination in children and adolescents: Analysis of data reported to EudraVigilance | Maltezou HC | Study estimating rates of anaphylaxis after mRNA vaccination in children and adolescents in spontaneous reports from EudraVigilance | 0-17 | BNT162b2, mRNA-1273 | no comparator, specific disease |
| 37213095 | Safety of the BNT162b2 COVID-19 Vaccine in Children Aged 5 to 17 Years | Hu M | Study using claims from three commercial US claims databases for potential AEFIs, comparing rates with historical data to detect potential signals in real-time | 5-17 | BNT162b2 | Historical comparator |  |
| 37091730 | | Myocarditis/pericarditis following vaccination with BNT162b2, CoronaVac, and ChAdOx1 among adolescent and adult in Malaysia | Ab Rahman N | Population-based cohort, presenting cumulative incidence and observed/expected rates of myocarditis/pericarditis using historical comparator | >12-60+, has own 12-17 | BNT162b2, CoronaVac, ChAdOx1 | historical comparator, specific disease |
| 37226552 | | Safety of heterologous ChAdOx1-S/BNT162b2 primary schedule versus homologous BNT162b2 vaccination: Insights from an Italian post-marketing study, 2021 | Fortunato F | Study using active surveillance data comparing ChadOx/BNT162b2 vs BNT162b2/BNT162b2 schedules | 18-60 | BNT162b2, ChAdOx1 | between-group comparison, local/systemic reactions |
| 37377545 | | A comparative analysis on serious adverse events reported for COVID-19 vaccines in adolescents and young adults | Cappelletti-Montano B | Study doing a comparative analysis on serious adverse events, covid vs influenza, HPV, monkeypox vaccines | 12-17, 18-49 | COVID-19, Influenza, HPV, Monkeypox | no comparator, between-group comparison, grouped outcome |
| 37400660 | | Urological complications after COVID 19 vaccine according to age, sex and manufacturer | Shim SR | Study using VAERS data to investigate urological complications post-vaccination, only report cumulative incidence rate | <18, 18-64, >64 | BNT162b2, mRNA-1273, Ad26.COV2.S | no comparator, specific group of diseases |
| 37556109 | | Adverse Events Following the BNT162b2 mRNA COVID-19 Vaccine (Pfizer-BioNTech) in Aotearoa New Zealand | Walton M | Cohort study reporting observed/expected (historical) rates using national health records | >5-80+: has ≤19 as a category | BNT162b2 | Historical comparator, cohort study |
| 37568373 | | Myocarditis and Pericarditis Post-mRNA COVID-19 Vaccination: Insights from a Pharmacovigilance Perspective | Alami A | VAERS retrospective pharmacovigilance analysing spontaneous reports of myo/peri comparing age, sex, and vaccine dose, comparing mRNA vaccines to all other vaccines | 12-17, 18-24 | BNT162b2, mRNA-1273 | Between-group comparison |
| 38442719 | | Adverse Events Following COVID-19 Vaccination in Adolescents: Insights From Pharmacovigilance Study of VigiBase | Kim DH | Disproportionality study using VigiBase comparing adverse events in adolescents after covid-19 vaccines vs other vaccines | 12-17 | All vaccines in VigiBase | Between-group comparison |
| 38615382 | | Comparative safety profile of bivalent and original COVID-19 mRNA vaccines regarding myocarditis/pericarditis: A pharmacovigilance study | Chen C | Study using VAERS spontaneous reports to compare monovalent vs bivalent mRNA vaccination, reporting on clinical characteristics, demographics and myo/pericarditis risk | 0.5-65+ | BNT162b2, mRNA-1273 | Between-group comparison |
| 38656578 | | Safety of Ancestral Monovalent BNT162b2, mRNA-1273, and NVX-CoV2373 COVID-19 Vaccines in US Children Aged 6 Months to 17 Years | Hu M | Study using a cohort from claims databases, evaluating if statistical signals for health outcomes were detected by comparing to a historical cohort | 0.5-17 | BNT162b2, mRNA-1273, NVX-CoV2373 | Historical comparator |
| 38724677 | | Multisystemic inflammatory syndrome in children and the BNT162b2 vaccine: a nationwide cohort study | Schwartz N | Nationwide cohort study presenting the MIS-C risk difference between vaccinated and unvaccinated patients | 0.5-18 | BNT162b2 | Between-group comparison, specific disease |
| 38988090 | | Comparative safety of monovalent and bivalent mRNA COVID-19 booster vaccines in adolescents aged 12 to 17 years in the Republic of Korea | Ko M | Study on spontaneous reports of AEFIs from doses administered to adolescents comparing bivalent vs monovalent booster vaccines | 12-17 | BNT162b2 | Between-group comparison |
| 39427003 | | Global burden of vaccine-associated Guillain-Barré syndrome over 170 countries from 1967 to 2023 | Jeong YD | Study using spontaneous reports from VigiBase comparing different vaccines on Guillain-Barrè Syndrome risk | 0-65+ | All vaccines in VigiBase 1967-2023 | Between-group comparison, specific disease |
| 39444354 | | The impact of COVID-19 status and vaccine type following the first dose on acute heart disease: A nationwide retrospective cohort study in South Korea | Yun C | Cohort study on acute heart diseases using data from the National Health Insurance Service COVID-19 database, comparing age groups and vaccine types | 10-70+ | mRNA (BNT162b2 and mRNA-1273) and non-mRNA (AZD1222, JNJ-78436735 (Ad26.COV2.S), NVX-CoV2373) | Between-group comparison, specific disease |
| 39527343 | | PaedVacCOVID - safety of the BNT162b2 vaccine against the SARS-CoV-2 in children with and without comorbidities aged 5 to 11 years | Holzwarth S | Prospective cohort study on vaccine safety using questionnaires comparing comorbid vs healthy | 5-11 | BNT162b2 | Between-group comparison, local/systemic reactions |
|  | **Case report or case series (n=41)** |  |  |  |  |  |  |
| 34166671 | | Recurrence of Acute Myocarditis Temporally Associated with Receipt of the mRNA Coronavirus Disease 2019 (COVID-19) Vaccine in a Male Adolescent | Minocha PK | Case report of 17-year-old male with recurrent acute myocarditis after 2nd dose BNT162b2 | 17 | BNT162b2 | Case report/series |
| 34180390 | | Self-limited myocarditis presenting with chest pain and ST segment elevation in adolescents after vaccination with the BNT162b2 mRNA vaccine | Park J | Case report of two adolescent males with myocarditis after BNT162b2 vaccination | 15.5 | BNT162b2 | Case report/series |
| 34228985 | | Myopericarditis After the Pfizer Messenger Ribonucleic Acid Coronavirus Disease Vaccine in Adolescents | Schauer J | Case series of myopericarditis after 2nd dose BNT162b2 vaccination | 12-17 | BNT162b2 | Case report/series |
| 34374740 | | Association of Myocarditis With BNT162b2 Messenger RNA COVID-19 Vaccine in a Case Series of Children | Dionne A | Case series of myocarditis associated with BNT162b2 in children | 12-18 | BNT162b2 | Case report/series |
| 34756746 | | STEMI Mimic: Focal Myocarditis in an Adolescent Patient After mRNA COVID-19 Vaccine | Azir M | Case report of 17-year-old male who developed focal myocarditis after BNT162b2 vaccination | 17 | BNT162b2 | Case report/series |
| 34955521 | | Multisystem Inflammatory Syndrome in a Previously Vaccinated Adolescent Female With Sickle Cell Disease | DeJong J | Case report of MIS-C in adolescent female with sickle cell disease |  | BNT162b2 | Case report/series |
| 34996671 | | Cardiac Tamponade After COVID-19 Vaccination | Hryniewicki AT | Case report of 18-year-old man with acute pericarditis | 18 | BNT162b2 | Case report/series |
| 35082004 | | A paediatric case of myopericarditis post-COVID-19 mRNA vaccine | Türe M | Case report of myocarditis in 14-year-old male after 2nd dose BNT162b2 vaccine | 14 | BNT162b2 | Case report/series |
| 35157759 | | Autopsy Histopathologic Cardiac Findings in 2 Adolescents Following the Second COVID-19 Vaccine Dose | Gill JR | Case report/study describing autopsy histopathology findings in two adolescents after 2nd mRNA vaccine dose with possible myocarditis | - | BNT162b2 | Case report/series |
| 35220345 | | Acute Vulvar Aphthous Ulceration After COVID-19 Vaccination: 3 Cases | Wijaya M | Case series of 3 cases of acute vulvar aphthous ulceration after COVID-19 vaccination |  | BNT162b2, ChAdOx1 | Case report/series |
| 35256374 | | Acute-onset dacryoadenitis following immunisation with mRNA COVID-19 vaccine | Murphy T | Case report of acute onset dacryoadenitis after mRNA covid vaccine | 14 | BNT162b2 | Case report/series |
| 35354564 | | Multisystem inflammatory syndrome in children (MIS-C) possibly secondary to COVID-19 mRNA vaccination | Wangu Z | Case report of MIS-C after 2nd dose BNT162b2 | - | BNT162b2 | Case report/series |
| 35373880 | | COVID-19 vaccine (mRNA BNT162b2) and COVID-19 infection-induced thrombotic thrombocytopenic purpura in adolescents | Vorster L | Case report on three adolescents with thrombotic thrombocytopenic purpura | 15-19 | BNT162b2 | Case report/series |
| 35385678 | | Two adolescent cases of acute tubulointerstitial nephritis after second dose of COVID-19 mRNA vaccine | Choi JH | Case report of two cases of acute tubulointerstitial nephritis following 2nd dose mRNA vaccination | 12-17 | BNT162b2 | Case report/series |
| 35389949 | | Delayed-onset Anaphylaxis After mRNA-Based COVID-19 Vaccination in an Adolescent Male | Shrestha P | Case report on delayed anaphylaxis in 16-year-old male after BNT162b2 | 16 | BNT162b2 | Case report/series |
| 35470603 | | A Case of Myocarditis Presenting With a Hyperechoic Nodule After the First Dose of COVID-19 mRNA Vaccine | Park S | Case report of myocarditis with hyperechoic nodule after BNT16b2 vaccination | 17 | BNT162b2 | Case report/series |
| 35561428 | | COVID-19 mRNA vaccine-associated encephalopathy, myocarditis, and thrombocytopenia with excellent response to methylprednisolone: A case report | Asaduzzaman M | Case report on mRNA vaccine associated encephalopathy, myocarditis and thrombocytopenia | 15 | BNT162b2 | Case report/series |
| 35698236 | | Chilblain-like lesions onset during SARS-CoV-2 infection in a COVID-19-vaccinated adolescent: case report and review of literature | Paparella R | Case report and literature review of chilblain-like lesions during covid infection in BNT162b2 vaccinated adolescent | 14 | BNT162b2 | Case report/series |
| 35729514 | | Sibling cases of gross hematuria and newly diagnosed IgA nephropathy following SARS-CoV-2 vaccination | Uchiyama Y | Case report on siblings with gross hematuria and IgA nephropathy following covid vaccination | 15-18 | BNT162b2 | Case report/series |
| 35854121 | | A child with crescentic glomerulonephritis following SARS-CoV-2 mRNA (Pfizer-BioNTech) vaccination | Kim S | Case report of a 16-year-old with crescentic glomerulonephritis after BNT162b2 vaccination | 16 | BNT162b2 | Case report/series |
| 35914312 | | Acute Rejection Following COVID-19 Vaccination in Penetrating Keratoplasty in a Young Male - A Case Report and Review of Literature | Marziali E | Case report and literature review of acute rejection in penetrating keratoplasty following BNT162b2 vaccination | 15 | BNT162b2 | Case report/series |
| 35962744 | | Clinical and immunological data from chronic urticaria onset after mRNA SARS-CoV-2 vaccines | Pescosolido E | Case series of 32 patients developing chronic urticaria within days after mRNA vaccination, shows clinical and immunological data on these | 16-66 | BNT162b2, mRNA-1273 | Case report/series |
| 35991822 | | Rhabdomyolysis after BNT162b2 mRNA Covid-19 vaccine in an adolescent male | Sutcu M | Case report of Rhabdomyolysis after BNT162b2 vaccination | 16 | BNT162b2 | Case report/series |
| 36107725 | | Severe refractory warm autoimmune haemolytic anaemia after the SARS-CoV-2 Pfizer-BioNTech vaccine (BNT162b2 mRNA) managed with emergency splenectomy and complement inhibition with eculizumab | Jackson EM | Case report on male teen with liver transplant and haemolytic anemia presenting with severe refractory warm autoimmune haemolytic anemia after BNT162b2 |  | BNT162b2 | Case report/series |
| 36228169 | | Case Report: Retinal Vasculitis in Two Adolescents After COVID-19 Vaccination | Mohamed S | Case report on two adolescents with retinal vasculitis following COVID-19 vaccination |  |  | Case report/series |
| 36379527 | | Acute myocarditis after COVID-19 vaccination | Saadi SM | Case report on 18-year-old male with myocarditis after mRNA-1273 | 18 | mRNA-1273 | Case report/series |
| 36409362 | | Severe rhabdomyolysis secondary to COVID-19 mRNA vaccine in a teenager | Pucchio A | Case report of rhabdomyolysis in 16-year-old male following BNT162b2 vaccination | 16 | BNT162b2 | Case report/series |
| 36442910 | | Post-COVID mRNA vaccine myocarditis in children: report of two cases | Shamekh A | Case report of myocarditis/pericarditis in two children following BNT162b2 vaccination |  |  | Case report/series |
| 36482489 | | Optic disc hemorrhage in a young female following mRNA coronavirus disease 2019 vaccination: a case report | Tsuda K | Case report of optic disc hemorrhage in a young female after 2nd dose BNT162b2 vaccination | 18 | BNT162b2 | Case report/series |
| 36483824 | | Multiple cranial nerve palsies with small angle exotropia following COVID-19 mRNA vaccination in an adolescent: A case report | Lee H | Case report on cranial nerve palsies with small angle exotropia following mRNA vaccination in adolescent | 14 | BNT162b2 | Case report/series |
| 36584585 | | Complex regional pain syndrome after mRNA-based COVID-19 vaccination | Horisawa S | Case report of 17-year-old girl with complex regional pain syndrome following BNT162b2 vaccination | 17 | BNT162b2 | Case report/series |
| 36641359 | | Acute psychosis induced by mRNA-based COVID-19 vaccine in adolescents: A pediatric case report | Lien YL | Case report on acute psychosis following BNT162b2 vaccine | 15 | BNT162b2 | Case report/series |
| 36736453 | | Probable association between mRNA COVID-19 vaccine and opsoclonus-myoclonus-ataxia syndrome | Deniz A | Case report on opsoclonus-myoclonus-ataxia syndrome after BNT162b2 vaccination | 15 | BNT162b2 | Case report/series |
| 36990036 | | A case of fatal multi-organ inflammation following COVID-19 vaccination | Nushida H | Case report of fatal multi-organ inflammation in 14-year-old girl | 14 | BNT162b2 | Case report/series |
| 36999413 | | Myocarditis and Pericarditis Related to mRNA COVID-19 Vaccination: A Case Report | María PLE | Case report of myo/pericarditis related to mRNA COVID-19 vaccination | 15 | BNT162b2, mRNA-1273 | Case report/series |
| 37069805 | | Acute vulvar ulcer as a possible adverse event of gene-based COVID-19 vaccines: A review of 14 cases | Kanetani H | Case review of 14 cases of acute vulvar ulcer | mean 16.9, SD +/- 5 | BNT162b2, ChAdOx1 | Case report/series |
| 37256729 | | Bivalent mRNA COVID-19 Vaccine-Related Pericarditis on 18 F-FDG PET/CT | Wu YC | Case report of suspected pericarditis after second booster dose of bivalent mRNA vaccine | 13 | booster bivalent COVID-19 mRNA vaccine | Case report/series |
| 37260797 | | Herpes Zoster Virus Reactivation in a 16 Year Old Female Post COVID-19 Vaccine. Case report and Review of the Literature | Alharbi S | Case report and literature review of herpes Zoster reactivation following vaccination | 16 | BNT162b2 | Case report/series |
| 37303596 | | Relapsing myocarditis following initial recovery of post COVID-19 vaccination in two adolescent males - Case reports | Amodio D | Case reports on two cases with myocarditis after 2nd dose BNT162b2 | 15,16 | BNT162b2 | Case report/series |
| 37484868 | | Myocarditis following COVID-19 mRNA vaccinations: Twin and sibling case series | Shenton P | Case series on twins and siblings of myocarditis after mRNA vaccination, presenting 6 young males | 13-17 | BNT162b2, mRNA-1273 | Case report/series, specific group |
| 38164230 | | Graves' disease post-COVID-19 m-RNA vaccine in pediatric age group | Al-Jahhafi AS | Case report of Graves Disease | 17 | BNT162b2 | Case report/series |
|  | **Studies in specific populations (n=24)** |  |  |  |  |  |  |
| 34233234 | | The BNT162b2 mRNA COVID-19 vaccine in adolescents and young adults with cancer: A monocentric experience | Revon-Riviere G | Study on safety and efficacy of BNT162b2 in adolescents and young adults with cancer | 16-21 | BNT162b2 | Specific group |
| 34492161 | | Safety and tolerability of the COVID-19 messenger RNA vaccine in adolescents with juvenile idiopathic arthritis treated with tumor necrosis factor inhibitors | Dimopoulou D | Study on safety and tolerability of BNT162b2 in adolescents with juvenile idiopathic arthritis treated with TNF inhibitors | 16-21 | BNT162b2 | Specific group, local/systemic reactions |
| 34978376 | | Early experience of COVID-19 vaccine-related adverse events among adolescents and young adults with rheumatic diseases: A single-center study | Haslak F | Study on early experiences with safety in adolescents and young adults with rheumatic diseases | median 15, range 12-21 | BNT162b2, CoronaVac | Specific group |
| 35179569 | | Safety and immunogenicity of BNT162b2 mRNA COVID-19 vaccine in adolescents with rheumatic diseases treated with immunomodulatory medications | Heshin-Bekenstein M | Study investigating the safety and immunogenicity of BNT162b2 in adolescents and young adults with AIIRDs (juvenile-onset autoimmune inflammatory rheumatic diseases) | 12-18, 18-21 | BNT162b2 | Specific group |
| 35891273 | | Evaluation of Safety and Immunogenicity of BNT162B2 mRNA COVID-19 Vaccine in IBD Pediatric Population with Distinct Immune Suppressive Regimens | Cotugno N | Study on safety and immunogenicity of BNT162b2 in IBD pediatric population with distinct immune suppression | 12-20 in IBD, 24-43 in controls | BNT162b2 | Specific group |
| 35960521 | | Tolerability of COVID-19 Infection and Messenger RNA Vaccination Among Patients With a History of Kawasaki Disease | Beckley M | Study on tolerability of covid infection and mRNA vaccination in patients with Kawasaki disease | 5-21 | BNT162b2, mRNA-1273 | Specific group |
| 35964130 | | Humoral and cellular immune response to mRNA SARS-CoV-2 BNT162b2 vaccine in adolescents with rheumatic diseases | Udaondo C | Study on safety and efficacy of BNT162b2 in adolescents with rheumatic diseases | 12-18 | BNT162b2 | Specific group |
| 36016153 | | Neutralizing Antibody Response, Safety, and Efficacy of mRNA COVID-19 Vaccines in Pediatric Patients with Inflammatory Bowel Disease: A Prospective Multicenter Case-Control Study | Lee KJ | Study on serological response, safety and efficacy of BNT162b2 vaccine in children with IBD | 12-18 | BNT162b2 | Specific group |
| 36203563 | | Safety and immunogenicity of 3 doses of BNT162b2 and CoronaVac in children and adults with inborn errors of immunity | Leung D | Clinical trial on safety and immunogenicity of 3rd dose BNT and CoronaVac in children and adults with inborn errors of immunity | 5-51 (median 17) | BNT162b2, CoronaVac | Clinical Trial, specific group |
| 36331234 | | Immunogenicity and safety of SARS-CoV-2 vaccine with immunosuppressive agents | Kamei K | Study on immunogenicity and safety of mRNA vaccines in children and adolescents on immunosuppressants | median 18.1 | mRNA vaccine | Specific group |
| 36966598 | | Humoral Immune Response Following SARS-CoV-2 mRNA Vaccination and Infection in Pediatric-Onset Multiple Sclerosis | Breu M | Study on vaccine response in pediatric-onset Multiple Sclerosis patients | median 17.43 | BNT162b2, mRNA-1273 | Specific group, vaccine response, local/systemic reactions |
| 36969290 | | Safety of the COVID-19 vaccination in children with juvenile idiopathic arthritis-A observational study from two pediatric rheumatology centres in Poland | Opoka-Winiarska V | Study on safety of covid vaccination on children with Juvenile Idiopathic Arthritis | 5.4-17.2 | BNT162b2 | Specific group |
| 36987787 | | Adverse Events Associated with COVID-19 Vaccination in Adolescents with Endocrinological Disorders: A Cross-Sectional Study | Erbaş İM | Cross-sectional study on adverse events in adolescent patients with endocrinological disorders | 12-18 (median 15.5) | BNT162b2, CoronaVac | Specific group, local/systemic reactions |
| 37026809 | | Coronavirus disease 2019 vaccine in pediatric post-kidney transplantation | Ajlan AA | Study investigating the safety and efficacy of BNT162b2 in pediatric kidney transplant patients | 12-16 | BNT162b2 | specific group, local/systemic reactions |
| 37112731 | | Safety and Immunogenicity Following the Second and Third Doses of the BNT162b2 mRNA COVID-19 Vaccine in Adolescents with Juvenile-Onset Autoimmune Inflammatory Rheumatic Diseases: A Prospective Multicentre Study | Heshin-Bekenstein M | Study on long-term safety and immunogenicity after BNT162b2 vaccination in adolescents with Juvenile-onset autoimmune inflammatory rheumatic diseases | 12-18 | BNT162b2 | Specific group |
| 37157992 | | Hesitancy, reactogenicity and immunogenicity of the mRNA and whole-virus inactivated Covid-19 vaccines in pediatric neuromuscular diseases | Yu MKL | Study on safety of BNT162b2 and CoronaVac in patients with neuromuscular disease, also investigates vaccine hesitancy | 2-21 | BNT162b2, CoronaVac | Specific group, local/systemic |
| 37435175 | | Immunogenicity, safety and clinical outcomes of the SARS-CoV-2 BNT162b2 vaccine in adolescents with type 1 diabetes | Emeksiz HC | Study on immunogenicity, reactogenicity and clinical outcomes of BNT162b2 in adolescents with type 1 diabetes | mean age 14.7 | BNT162b2 | Specific group |
| 37679040 | | mRNA COVID-19 Vaccination Does Not Exacerbate Symptoms or Trigger Neural Antibody Responses in Multiple Sclerosis | Blanco Y | Study on patients with Multiple Sclerosis, measuring antibodies and exacerbation | mean 44.1 | BNT162b2, mRNA-1273 | Specific group, local/systemic reactions |
| 37880380 | | Safety of BNT162b2 mRNA COVID-19 vaccine in children with chronic kidney disease: a national population study from South Korea | Choe YJ | Study to assess the rates of AEFIs following BNT162b2 vaccination in children with chronic kidney disease | 12-17 | BNT162b2 | Specific group |
| 38632665 | | Efficacy and safety of BNT162b2 mRNA vaccine in a cohort of 90 transfusion dependent thalassemia patients | Marziali M | Study on efficacy and safety of BNT162b2 in transfusion dependent thalassemia patients | 3-72, median 40 | BNT162b2 | Specific group |
| 38821068 | | COVID-19 vaccination among adolescents and young adults with chronic kidney conditions: a single-center experience | Baltu D | Single-center AEFI using questionnaires on safety in people with chronic kidney conditions | Mean 16.9 | BNT162b2, CoronaVac | Specific group |
| 38858672 | | Uptake, effectiveness and safety of COVID-19 vaccines in individuals at clinical risk due to immunosuppressive drug therapy or transplantation procedures: a population-based cohort study in England | Chen DT | Population based cohort reporting COVID-19 vaccine uptake, effectiveness and safety in immunocompromised individuals | 12+ (mean age 43.2) | BNT162b2, mRNA-1273, ChAdOx1 | Specific group |
| 38915282 | | Cardiovascular Safety of COVID-19 Vaccination in Patients With Cancer: A Self-Controlled Case Series Study in Korea | Ryu JH | SCCS study on cardiovascular safety in cancer patients | 12-65+ (mean 70.4) | BNT162b2, mRNA-1273, ChAdOx1, Ad26.COV2.S | Specific group |
| 39387432 | | Safety and efficacy of COVID-19 vaccines in children and adolescents with cancer | Kurucu N | Safety and vaccine efficacy in children and adolescents with cancer | mean 16.9 | BNT162b2, CoronaVac | Specific group, local/systemic reactions |
|  | | **Reviews and meta-analyses (n=37)** |  |  |  |  |  |
| 33907509 | | Review of Covid-19 vaccine clinical trials - A puzzle with missing pieces | Kwok HF | Review of covid-19 vaccine clinical trials and data available | 18-55, 16-55, 55+ | BNT162b2, mRNA-1273, ChAdOx1, Ad26.COV2.S, NVX-CoV2373, CoronaVac | Review |
| 34100150 | | Tozinameran (BNT162b2) Vaccine: The Journey from Preclinical Research to Clinical Trials and Authorization | Khehra N | Review on the BNT162b2 vaccine from preclinical research to clinical trials and authorization |  | BNT162b1, BNT162b2 | Review |
| 34281357 | | Myocarditis With COVID-19 mRNA Vaccines | Bozkurt B | Narrative review of myocarditis after mRNA vaccines, summarizing literature, discussing mechanism, management, research priorities and benefit-risk | - | BNT162b2, mRNA-1273 | Review |
| 34707602 | | Efficacy, Immunogenicity and Safety of COVID-19 Vaccines: A Systematic Review and Meta-Analysis | Sharif N | Systematic review and meta-analysis of efficacy, immunogenicity and safety of covid-19 vaccines | 16-80+ | BNT162b2, mRNA-1273, Ad5-nCov, Ad26.COV2.S, BBIBP-CorV, CoronaVac, NVX-CoV2373, MF59-adjuvanted spike glycopreotein-clamp | Review |
| 34739716 | | COVID-19 Vaccination in Pregnancy, Paediatrics, Immunocompromised Patients, and Persons with History of Allergy or Prior SARS-CoV-2 Infection: Overview of Current Recommendations and Pre- and Post-Marketing Evidence for Vaccine Efficacy and Safety | Luxi N | Narrative review of recommendations, vaccine efficacy and safety of COVID-19 vaccination in pregnancy, pediatrics, immunocompromised, allergic and prior infected individuals | - | BNT162b2, mRNA-1273, ChAdOx1, Ad26.COV2.S | Review |
| 34808708 | | A review of COVID-19 vaccination and the reported cardiac manifestations | Ho JSY | Review of cardiac adverse events | - | BNT162b2, mRNA-1273, ChAdOx1, Ad26.COV2.S, CoronaVac, BBIBP-CorV, Gam-COVID-Vac | Review |
| 34862617 | | Clinical characteristics and prognostic factors of myocarditis associated with the mRNA COVID-19 vaccine | Woo W | Review of case reports and series of myocarditis after mRNA vaccination presenting clinical characteristics and prognostic factors | median 17 (14-70) | BNT162b2, mRNA-1273 | Review |
| 34960761 | | Cardiomyopathy Associated with Anti-SARS-CoV-2 Vaccination: What Do We Know? | Parra-Lucares A | Review of cardiomyopathy associated with COVID-19 vaccination | 16 | BNT162b2 | Review |
| 35238384 | | Myocarditis post-SARS-CoV-2 vaccination: a systematic review | Goyal M | Systematic review of myo(peri)carditis after COVID-19 vaccination focusing on clinical features, laboratory findings, treatment modalities and outcomes | >12 | BNT162b2, Ad26.COV2. S, ChAdOx1, COVAXIN, mRNA-1273 | Review |
| 35244020 | | [COVID-19 among children and adolescents] | Schelde AB | Review on covid-19 infection and vaccination (safety and effectiveness) in children and adolescents | 0-17, 5-17 | BNT162b2, mRNA-1273 | Review |
| 35289493 | | COVID-19 mRNA vaccines and myopericarditis | Gnanenthiran SR | Review on myopericarditis after mRNA vaccination incidence, risk factors, manifestation, diagnosis, management, prognosis, mechanisms, and Australian vaccine recommendations |  | BNT162b2, mRNA-1273 | Review |
| 35421376 | | Myopericarditis following COVID-19 vaccination and non-COVID-19 vaccination: a systematic review and meta-analysis | Ling RR | Systematic review and meta-analysis of myopericarditis following vaccination (covid and non) | <30, ≥30 | mRNA and non-mRNA | Review |
| 35493393 | | Safety, Immunogenicity, and Efficacy of COVID-19 Vaccines in Adolescents, Children, and Infants: A Systematic Review and Meta-Analysis | Du Y | Systematic review and meta-analysis of safety, immunogenicity and efficacy of covid-19 vaccines in adolescents | 0-17 | BNT162b2, mRNA-1273, ChAdOx1, Ad26.COV2.S, NVX-CoV2373, CoronaVac | Review, local/systemic reactions |
| 35705969 | | Safety and efficacy of COVID-19 vaccines in children and adolescents: A systematic review of randomized controlled trials | Tian F | Systematic review of RCTs of safety and efficacy of covid vaccines in children and adolescents | <18 | BNT162b2, mRNA-1273, CoronaVac, BBIBP-CorV, Ad5-nCov, ZyCov-D | Review, local/systemic reactions |
| 35716417 | | Immunologic response, Efficacy, and Safety of Vaccines against COVID-19 Infection in Healthy and immunosuppressed Children and Adolescents Aged 2 - 21 years old: A Systematic Review and Meta-analysis | Sadeghi S | Systematic review and meta-analysis of immunologic response, efficacy and safety of covid vaccines in healthy and immunosuppressed 2-21 year olds | <21 | BNT162b2, mRNA-1273, CoronaVac, BBIBP-CorV, Ad5-nCoV-S, Ad26.COV2.S, ZyCoV-D | Review |
| 35796029 | | Comparing reactogenicity of COVID-19 vaccines: a systematic review and meta-analysis | Sutton N | Systematic review and meta-analysis comparing reactogenicity of 20 different COVID-19 vaccines | 12+ | BNT162b2, mRNA-1273, ChAdOx1, Ad26.COV2.S, CoronaVac, NVX-CoV2373, Ad5-nCov, BBIBP-CorV, Gam-COVID-Vac, BBV152, Inactivated vaccine IMBCAMS, QazCovid-in, WIBP, CVnCoV, EpiVacCorona, MVC-COV1901, SCB-20199, Sf9, V-01, ZF2001 | Review, local/systemic reactions |
| 35851461 | | Myocarditis Following COVID-19 Vaccination | Marschner CA | Review on myocarditis incidence, risk factors, clinical presentation, imaging findings, mechanisms, treatment and prognosis of myocarditis following COVID-19 mRNA vaccination |  | BNT162b2, mRNA-1273 | Review |
| 36159608 | | COVID-19 vaccination and myocarditis: A review of current literature | Dhaduk K | Review on myocarditis after COVID-19 vaccination, describing incidence, clinical presentation, possible mechanism, management, outcomes and risk assessment | - | BNT162b2, mRNA-1273 | Review |
| 36291403 | | Intrinsic Kidney Pathology in Children and Adolescents Following COVID-19 Vaccination: A Systematic Review | Wu HHL | Systemic review of case reports/series on intrinsic kidney pathology in children and adolescents following covid vaccination | 12-17 | BNT162b2, "Brand not noted" | Review |
| 36367429 | | Safety of COVID-19 Pfizer-BioNtech (BNT162b2) mRNA vaccination in adolescents aged 12-17 years: A systematic review and meta-analysis | Katoto PDMC | Systematic review and meta-analysis on safety of BNT162b2 in adolescents 12-17 | 12-18 | BNT162b2 | Review |
| 36469338 | | Myopericarditis After COVID-19 mRNA Vaccination Among Adolescents and Young Adults: A Systematic Review and Meta-analysis | Yasuhara J | Systematic review and meta-analysis of incidence, clinical features and outcomes of Myopericarditis after mRNA vaccination among adolescent/young adults | 12-20 | BNT162b2, mRNA-1273 | Review |
| 36473651 | | Efficacy and safety of COVID-19 vaccines | Graña C | Cochrane review on trials on efficacy and safety of 12 different COVID-19 vaccines | no age restriction | BNT162b2, mRNA‐1273, ChAdOx1, Ad26.COV2.S, BBIBP‐CorV, BBV152, NVX‐CoV2373, CoronaVac, WIBP-CorV, FINLAY-FR-2, Gam-COVID-Vac, CVnCoV | Review |
| 36484136 | | Effectiveness of mRNA, protein subunit vaccine and viral vectors vaccines against SARS-CoV-2 in people over 18 years old: a systematic review | Sandoval C | Systematic review on effectiveness of mRNA, vector, protein subunit vaccines in adults | 18+ | BNT162b1, BNT162b2, mRNA-1273, ChAdOx1, ZF2001 | Review |
| 36594165 | | Development of myocarditis and pericarditis after COVID-19 vaccination in children and adolescents: A systematic review | Fatima M | Systematic review of myocarditis and pericarditis after COVID-19 vaccination in children and adolescents | 0-19 | BNT162b2, mRNA-1273 | Review |
| 36723827 | | Immunogenicity, effectiveness, and safety of COVID-19 vaccines among children and adolescents aged 2-18 years: an updated systematic review and meta-analysis | Gao P | Systematic review and meta-analysis of immunogenicity, effectiveness, and safety of COVID-19 vaccines in 2-18-year-olds | 2-18 | BNT162b2, mRNA-1273, CoronaVac, BBIBP-CorV, BBV152, PastoCoVac, Ad5-nCoV-S, ChAdOx1-S/nCoV-19, Ad26.COV2.S, ZyCoV-D | Review |
| 36847285 | | A Review of the Data Supporting Use of COVID-19 Vaccinations in the Pediatric Population | Stultz JS | Review of the effectiveness and safety of covid-19 vaccinations in pediatric population |  |  | Review |
| 36851090 | | COVID-19 Vaccination in Pediatrics: Was It Valuable and Successful? | Raslan MA | Review attempting to answer if pediatric COVID-19 vaccination was valuable/successful | 0.5-19 | mRNA, inactivated, adenoviral-based | Review |
| 36863817 | | Myocarditis Following COVID-19 Vaccination | Marschner CA | Review on incidence, risk factors, clinical presentation, imaging findings, proposed pathophysiologic mechanisms, treatment, and prognosis of myocarditis following COVID-19 vaccination | - | BNT162b2, mRNA-1273 | Review |
| 37078534 | | Immunogenicity and reactogenicity of COVID-19 Pfizer-BioNTech (Bnt162b2) mRNA vaccination in immunocompromised adolescents and young adults: a systematic review and meta-analyses | Katoto PD | Systemic review and meta-analysis of immunogenicity and reactogenicity BNT162b2 in immunocompromised adolescents and young adults | 12-24.3 | BNT162b2 | Review, local/systemic reactions |
| 37097556 | | Narrative Review of the Evolution of COVID-19 Vaccination Recommendations in Countries in Latin America, Africa and the Middle East, and Asia | Spinardi J | Narrative Review of vaccination recommendations and coverage in Latin America, Africa, Middle East and Asia | Adults, adolescents and children | BNT162b2, mRNA-1273, CoronaVac, ChAdOx1, Ad26.COV2.S, BBIBP-CorV, Ad5-nCoV, Gam-COVID-Vac, NVX-CoV2373 | Review |
| 37167355 | | Vaccination-Associated Myocarditis and Myocardial Injury | Altman NL | Review of clinical features and pathology of Myocarditis and vaccine-associated myocarditis | - | COVID-19 vaccines and non-COVID19 vaccines | Review |
| 37246067 | | Incidence of myopericarditis after mRNA COVID-19 vaccination: A meta-analysis with focus on adolescents aged 12-17 years | Guo BQ | Meta-analysis of myopericarditis after mRNA, focused on 12-17, reporting only pooled incidence | 12-17 | BNT162b2, mRNA-1273 | Meta-analysis |
| 37248893 | | Postural orthostatic tachycardia syndrome-like symptoms following COVID-19 vaccination: An overview of clinical literature | Tv P | Study summarizing evidence that has been reported on Postutal Orthostatic Tachiacardia syndrom following COVID-19 vaccination | 17-52 | BNT162b2, mRNA-1273, ChAdOx1 | Review, specific disease |
| 37487674 | | Considerations for vaccinating children against COVID-19 | Hart JD | Review of COVID-19 with a focus on low-income and middle-income countries, summarizing safety, efficacy and effectiveness in children and adolescents | Children and adolescents | COVID-19 vaccines | Review |
| 37550719 | | The protective effect of COVID-19 vaccines on developing multisystem inflammatory syndrome in children (MIS-C): a systematic literature review and meta-analysis | Hamad Saied M | Review and meta-analysis on COVID-19 vaccine effectiveness on MIS-C, and briefly on risk of MIS-C after vaccination | 5-20 | BNT162b2, mRNA-1273, Ad26.COV2.S | Review |
| 38010097 | | Development of mRNA nano-vaccines for COVID-19 prevention and its biochemical interactions with various disease conditions and age groups | Baig MMFA | Review with brief history of COVID 19, development of mRNA vaccines and adverse events in young adults, adolescents, pregnant women, elderly, allergic or infarction background | - | BNT162b2, mRNA-1273 | Review |
| 38882914 | | Efficacy, Effectiveness, and Safety of COVID-19 Vaccine Compared to Placebo in Preventing COVID-19 Infection among 12-17 Years Old: A Systematic Review | Gregorio GEV | Systematic review on efficacy, effectiveness and safety of COVID-19 vaccine in 12–17-year-olds | 12-17 | BNT162b2, mRNA-1273, ChAdOx1, BBIBP-CorV, CoronaVac | Review, local/systemic reactions |
|  | **Studies reporting local and systemic reactions (n=4)** |  |  |  |  |  |  |
| 34351881 | | COVID-19 Vaccine Safety in Adolescents Aged 12-17 Years - United States, December 14, 2020-July 16, 2021 | Hause AM | Study using adverse events report from VAERS (and a small analysis on v-safe) on BNT162b2 safety of adolescents 12-17 | 12-17 | BNT162b2 | Local/systemic reactions |
| 36217268 | | Adverse events after administration of the first and second doses of messenger RNA-based COVID-19 vaccines in Japanese subjects aged 12-18 years | Ogawa T | Early prospective study on AEs in Japanese 12-18-year-olds reporting only local and systemic reactions | 12-18 | BNT162b2, mRNA-1273 | Local/systemic reactions |
| 39484062 | | Safety of BNT162b2 COVID-19 Vaccine in Adolescent Patients of UP-PGH | Hao RJC | Retrospective cohort study assessing short-term safety of BNT162b2 using diaries | 12-17 | BNT162b2 | Local/systemic reactions |
| 39536790 | | Active Surveillance for Safety Monitoring of XBB.1.5-Containing COVID-19 mRNA Vaccines in Korea | Park B | Study reporting adverse events following XBB15 vaccination using surveys, reporting only local/systemic reactions | <19 (n = 31), 19-64, 65+ | XBB15, Influenza | Local/systemic reactions |
|  | **Clinical trials (n=9)** |  |  |  |  |  |  |
| 34379915 | | Evaluation of mRNA-1273 SARS-CoV-2 Vaccine in Adolescents | Ali K | Clinical trial reporting safety, efficacy and non-inferiority (compared to adults) of mRNA-1273 in adolescents | 12-17 | mRNA-1273 | Clinical trial |
| 35544369 | | Evaluation of mRNA-1273 Covid-19 Vaccine in Children 6 to 11 Years of Age | Creech CB | Clinical trial reporting safety, immunogenicity and efficacy of mRNA-1273 in 6–11-year-old children | 6-11 | mRNA-1273 | Clinical trial |
| 36260859 | | Evaluation of mRNA-1273 Vaccine in Children 6 Months to 5 Years of Age | Anderson EJ | Clinical trial reporting safety, immunogenicity and efficacy of mRNA-1273 vaccine in children <6 years | 0.5-5 | mRNA-1273 | Clinical Trial |
| 37331429 | | Reactogenicity, immunogenicity and breakthrough infections following heterologous or fractional second dose COVID-19 vaccination in adolescents (Com-COV3): A randomised controlled trial | Kelly E | Trial on Heterologous and fractional dose COVID-19 vaccine schedules in adolescents, comparing BNT162b2 vs. NVX-COV2373 | 12-16 | BNT162b2, NVX-CoV2373 | Clinical Trial |
| 39091673 | | Safety and durability of mRNA-1273-induced SARS-CoV-2 immune responses in adolescents: results from the phase 2/3 TeenCOVE trial | Figueroa AL | Study reporting data from clinical trial on safety and durability of mRNA-1273 immune responses in adolescents | 12-17 | mRNA-1273 | Clinical Trial |
| 39158584 | | Safety and Immunogenicity of an mRNA-1273 Booster in Children | Berthaud V | Clinical trial reporting safety and immunogenicity of mRNA-1273 booster in children | 0.5-5, 6-11 | mRNA-1273 | Clinical Trial |
| 39332418 | | Safety and immunogenicity of a single-dose omicron-containing COVID-19 vaccination in adolescents: an open-label, single-arm, phase 2/3 trial | Figueroa AL | Clinical Trial reporting safety, immunogenicity and inferred effectiveness of mRNA-1273 booster in adolescents | 12-17 | mRNA-1273 | Clinical Trial |
| 39504023 | | Safety of Simultaneous vs Sequential mRNA COVID-19 and Inactivated Influenza Vaccines: A Randomized Clinical Trial | Walter EB | Clinical trial on simultaneous administration of covid and influenza vaccines | 5+ (mean 33.4) | mRNA and influenza | Clinical Trial |
| 39836458 | | Safety and immunogenicity of an mRNA-1273 vaccine booster in adolescents | Figueroa AL | Clinical trial reporting safety and immunogenicity of mRNA-1273 booster in adolescents | 12-17 | mRNA-1273 | Clinical Trial |
|  | **Studies investigating one outcome (n=8)** |  |  |  |  |  |  |
| 35900992 | | Postmarketing active surveillance of myocarditis and pericarditis following vaccination with COVID-19 mRNA vaccines in persons aged 12 to 39 years in Italy: A multi-database, self-controlled case series study | Massari M | SCCS study on national data on myo/pericarditis in 12–39-year-olds | 12-39, 12-17, 18-29, 30-39 | BNT162b2, mRNA-1273 | Specific disease |
| 35993236 | | Risk of Myocarditis After Sequential Doses of COVID-19 Vaccine and SARS-CoV-2 Infection by Age and Sex | Patone M | SCCS study on national cohort investigating risk of myocarditis after COVID-19 vaccination | 13+ | BNT162b2, mRNA-1273, ChAdOx1 | Cohort study, Specific disease |
| 36871101 | | The effects of Covid-19 mRNA vaccine on adolescence gynecological well-being | Mohr-Sasson A | Cohort study in adolescents on effect of mRNA vaccine on gynecological well-being | 12-16 | BNT162b2 | Specific symptoms, Specific disease |
| 36973247 | | Risk of death following COVID-19 vaccination or positive SARS-CoV-2 test in young people in England | Nafilyan V | SCCS study on risk of death following COVID-19 vaccination or infection using data from a national cohort | 12-29 (12-17 separate) | mRNA (BNT162b2, mRNA-1273) and non-mRNA (ChAdOx1, unknown) | Specific outcome |
| 37285378 | | Risk of myocarditis and pericarditis after a COVID-19 mRNA vaccine booster and after COVID-19 in those with and without prior SARS-CoV-2 infection: A self-controlled case series analysis in England | Stowe J | SCCS study on myocarditis and pericarditis after COVID-19 mRNA vaccine booster and infection | 16-39, 40+, and 12-15, 16-24 | BNT162b2, mRNA-1273, ChAdOx1 | Specific disease |
| 38365960 | | Booster vaccination with SARS-CoV-2 mRNA vaccines and myocarditis in adolescents and young adults: a Nordic cohort study | Hviid A | Multinational cohort on booster vaccination with COVID-19 mRNA vaccines and myocarditis in adolescents and young | 12-39, 12-15, 16-24, 25-39 | BNT162b2, mRNA-1273 | Cohort on myocarditis, Specific disease |
| 39657695 | | The Impact of COVID-19 Vaccination on Thyroid Disease in 7 Million Adult and 0.2 Million Adolescent Vaccine Recipients | Bea S | SCCS study investigating incident and exacerbation of thyroid diseases after vaccination in adults and adolescents | 12+ | BNT162b2, mRNA-1273, ChAdOx1 | Specific disease |
| 39659558 | | mRNA COVID-19 vaccine safety among children and adolescents: a Canadian National Vaccine Safety Network cohort study | Soe P | Cohort study using self-reported surveys investigating myocarditis/pericarditis, anaphylaxis and health events (local reactions, consulted health case, emergency department visit, hospitalization) | 0.5-19 | BNT162b2, mRNA-1273 | Specific disease |
|  | **Similar, large cohort studies (n=3)** |  |  |  |  |  |  |
| 35254219 | | Adverse events of special interest following the use of BNT162b2 in adolescents: a population-based retrospective cohort study | Lai FTT | National population cohort study in adolescents investigating several AEFIs | 12-18 | BNT162b2 | Cohort study on AEFIs |
| 36802397 | | Safety and effectiveness of monovalent COVID-19 mRNA vaccination and risk factors for hospitalisation caused by the omicron variant in 0.8 million adolescents: A nationwide cohort study in Sweden | Nordström P | Study on safety and effectiveness of non-booster mRNA vaccination (with no time-window after vaccination), and risk factors for hospitalization, caused by omicron | 11-19 | BNT162b2, mRNA-1273 | Cohort study on AEFIs |
| 38802362 | | Safety outcomes following COVID-19 vaccination and infection in 5.1 million children in England | Copland E | SCCS study on several safety outcomes in adolescents using national cohort data, also present results from matched cohort study | 5-17 | BNT162b2, mRNA-1273, ChAdOx1 | Cohort study on AEFIs |
| 38130887 | | Nationwide safety surveillance of COVID-19 mRNA vaccines following primary series and first booster vaccination in Singapore | Dorajoo SR | Observed/expected and SCCS study on several potential AEFIs following mRNA vaccination in the Singaporean population | 5-70+ (12-17 by sex in supplement) | BNT162b2, mRNA-1273 | Cohort study on AEFIs |
| 36189425 | | BNT162b2 COVID-19 vaccination uptake, safety, effectiveness and waning in children and young people aged 12–17 years in Scotland | Rudan I | SCCS study on AEFIs following BNT162b2 vaccination in Scotland, reporting on type 1 diabetes, Vasculitis and inflammatory conditions, Epilepsy, chronic fatigue and myocarditis | 12-17 | BNT162b2 | Cohort study on AEFIs |

Abbreviations/explanations: WHO – World Health Organization; VigiBase – The WHOs pharmacovigilance database; SD – standard deviation; PEG – PolyEthylene Glycols; PFASs – perfluoroalkyl substances; IMBCAMS – inactivated vaccine from Institute of Medical Biology, Chinese Academy of Medical Sciences. VAERS – Vaccine Adverse Event Reporting Systems, the US national early warning system to detect possible safety problems in vaccines.

# *Supplementary Table 6: Preexisting risk conditions codes used for adjustment.*

| **Risk condition** | **ICD-10 codes** |
| --- | --- |
| Cerebral palsy | G80-G83 |
| Other Neurological/muscular disorders | G70-G73, Q00-Q04, Q06, Q07 |
| Downs | Q90 |
| Other Chromosomal conditions | Q91-Q93, Q95-Q99 |
| Cancer | C |
| Transplantation and Immune disorders | D80-D84, Z94 |
| Asthma | J45, R96* |
| Other Cardial/Pulmonary disease | Q20-28, Q30-Q34, I40-I43, I50, J44, J47, E84 |
| Diabetes mellitus | E10-E14 |
| Rheumatological conditions | M05-M09 |
| Inflammatory bowel disease | K50, K51, K52.3 |
| Celiac disease | K90.0 |
| Liver disorders | K75.4, K73, K74, K75.8, K76.0, K83.0 |
| Kidney disorders | N00-N08, N11-N19 |

*Supplementary Table 6 shows the International Statistical Classification of Diseases and Related Health Problems, Tenth Revision (ICD-10) codes defining the preexisting risk conditions used for adjustment. The preexisting risk conditions were defined by having two or more registrations of the relevant codes by 1^st^ January 2021. The code given includes all lower levels – e.g cancer includes all ICD-10 codes starting with C (i.e C00-C97).*

* International Classification of Primary Care, Second Edition (ICPC-2) Code
